# Design, synthesis and structure-activity relationship study of wollamide B; a new potential anti TB agent

**DOI:** 10.1371/journal.pone.0176088

**Published:** 2017-04-19

**Authors:** Henok Asfaw, Katja Laqua, Anna Maria Walkowska, Fraser Cunningham, Maria Santos Martinez-Martinez, Juan Carlos Cuevas-Zurita, Lluís Ballell-Pages, Peter Imming

**Affiliations:** 1 Institut für Pharmazie, Martin-Luther-Universität Halle-Wittenberg, Halle (Saale), Germany; 2 Diseases of the Developing World, Medicines Development Campus, GlaxoSmithKline, Tres Cantos, Madrid, Spain; Helsingin Yliopisto, FINLAND

## Abstract

Wollamide B is a cationic antimycobacterial cyclohexapeptide that exhibits activity against *Mycobacterium bovis (M*. *bovis)* (IC_50_ of 3.1 μM). Aiming to define its structural activity relationship (SAR), optimizing potency and pharmacokinetic properties, libraries of analogues were synthesized following a standard Fmoc-based solid phase peptide synthesis approach. The antimycobacterial activities of wollamide B and all the synthesized analogues were tested against *Mycobacterium tuberculosis* (*Mtb*) H37Rv. Parallely, *in vitro* drug metabolism and pharmacokinetic (ADME) profiling was done for the synthesized compounds to evaluate their drug likeness. Among the 25 synthesized wollamides five of them showed potent activities with MICs ≤ 3.1 μM and found to be nontoxic against human HepG2 cells up to 100 μM. The results of the *in vitro* ADME profiling revealed the remarkable plasma stability and very good aqueous solubility of the class in general while the metabolic stability was found to be moderate to low. Of particular note, compounds **7c** (MIC = 1.1 μM) and **13c** (0.6 μM) that exhibited good balance of antimycobacterial activity vs. optimal pharmacokinetic properties could be used as a new lead for further development.

## Introduction

Though enormous success has been achieved in progressively reducing tuberculosis (TB) associated mortality in the past two decades, the magnitude of the problem still remains significant. In 2014 alone 9.6 million people fell ill because of TB, out of which 1.5 million people lost their lives, an amount equivalent to the total population of Estonia [1]. This made TB worldwide, along with HIV, a leading cause of death among infectious diseases. The problem is even becoming more worrisome with rapid emergence of multidrug resistant and extensively drug resistant (MDR/XDR) TB. On the other hand, the development of new anti TB drugs has been almost nonexistent, with only one new drug marketed in the past 40 years [2]. The gap clearly signifies the importance of developing new anti-tubercular agents to properly address the problem we are now facing. One of the potential classes of compounds to become anti TB drugs is antimicrobial peptides (AMP).

AMPs occur naturally, in all forms of life, as a part of innate immunity or serving as a first line defense by exhibiting direct killing of a pathogen [3]. Recently, they have attracted increasing attention in the area of new antimicrobial drug development against drug resistant microbes including *Mtb*. The features that make them attractive drug candidates include their: proven spectrum of action against a wide range of pathogens, selective affinity to prokaryotic negatively charged cell envelopes, rapid cell killing action against the pathogen, and low immunogenicity. They often kill bacteria by interacting non-specifically with the bacterial cell wall and membrane, a mode of action that is less vulnerable for development of drug resistance [4–7].

The most common structural features of biologically active AMPs include cationicity with a net charge of at least +2 at physiological pH and amphiphilicity, while amino acid sequence and chain length vary considerably among them [8].

Several AMP classes that exhibited activities against various mycobacterial species have been reported in the past few decades. Their source, structural features, antimycobacterial potency, possible mechanism of action and drug likeness were extensively reviewed recently [9–15]. Based on the peptide size, antimycobacterial peptides are broadly classified as large (50–100 aa), intermediate (25–50 aa), low (10–24 aa) and ultra-small (2–10 aa).[16] Most of the peptides that were investigated for their antimycobacterial activities belong to high and intermediate size peptides and are obtained from a mammalian host immune system or from bacteria. To mention some of them: human neutrophile peptide [17], defensin [18], hepcidin [19]. NK-lysin [20], granulysin [21], human host defense ribonuclases (RNase) [22], Lysosomal ubiquitin derived peptide [23], lacticin 3147 [24] and E50-52 [25]. In spite of their promising antimycobacterial activity, these classes of compounds have common limitations that include difficulty of isolation from their source, high production cost, not being amenable to structural modification, extensive enzymatic degradation and possible immunogenicity.

Small and ultra-small cyclic antimycobacterial peptides could be good alternatives to larger ones as they basically contain the same structural features while avoiding most of the limitations mentioned [26, 27]. Several small and ultra-small cyclic peptides were reported to exert antimycobacterial activities, including griselmycins [28], depsidomycin [29], hytramycin [30], brunsvicamides [31], pyridomycin [32], hirsutellide A [33] and the wollamides [34].

**Fig 1:**
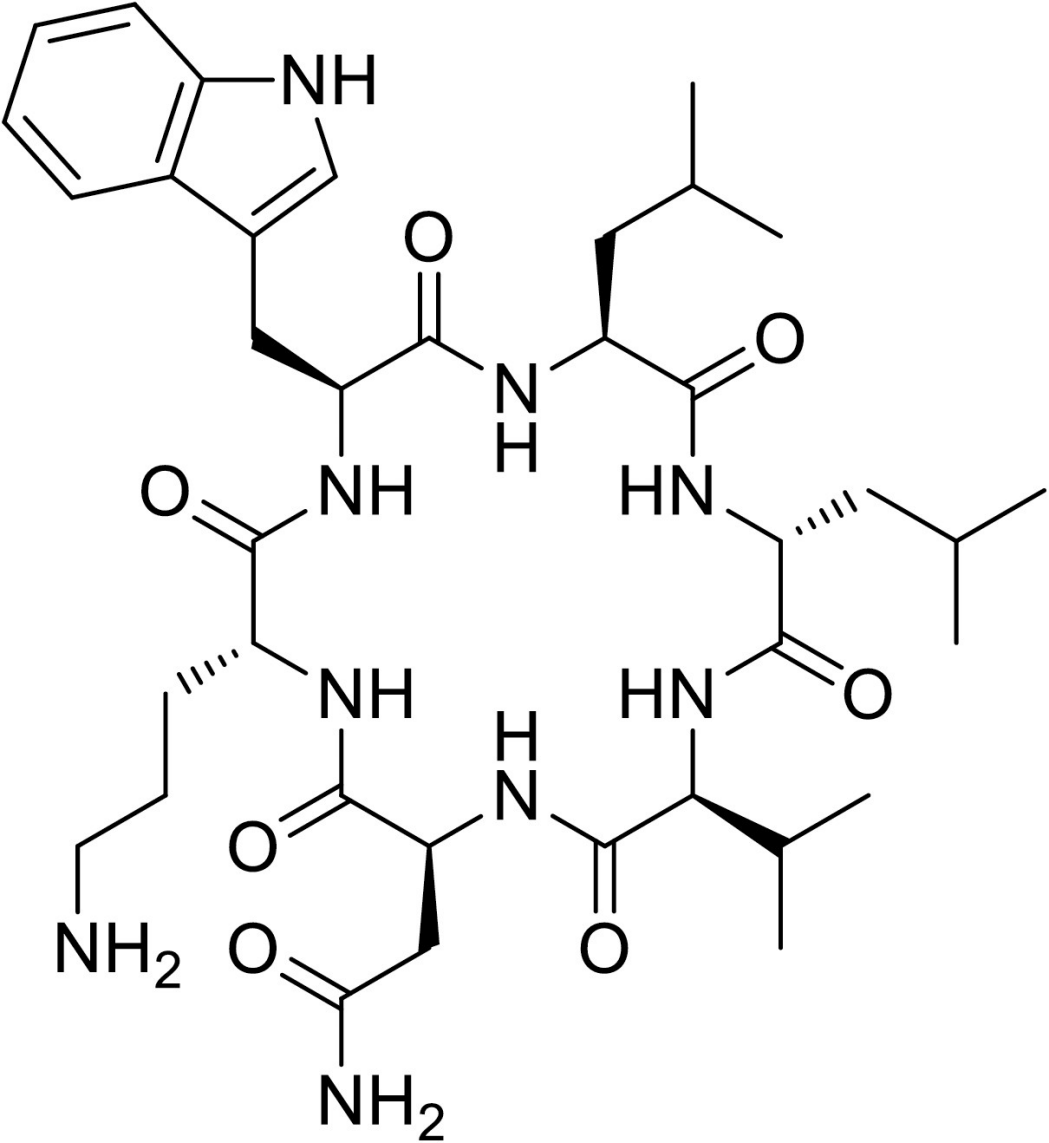
Structure of wollamide B.

Wollamide B (Fig 1) is a representative of a newer class of cyclic peptides that was isolated from an Australian soil derived *Streptomyces nov*. sp. (MST-115088). The compound had an IC_50_ of 3.1 μM against *M*. *bovis*. In addition, it was found to reduce the intracellular mycobacterial survival in murine bone marrow-derived macrophages [34]. Its activity against the common disease causing pathogen, *Mtb*, was not reported.

Structurally, wollamide B represents an ultra-small antimycobacterial cyclic hexapeptide consisted of two uncommon D-amino acids (D-Orn, D-Leu) and four L-amino acids (Fig 1). The presence of the basic amino acid ornithine and clusters of lipophilic amino acids imparts the typical cationicity and amphiphilicity to the molecule, *v*.*s*. Desotamides, structurally related to wollamide B but lacking the basic amino acid ornithine, were found to be inactive against mycobacteria indicating the importance of this amino acid for activity. On the basis of these data, a detailed study is warranted and will be reported here.

The present work is aimed at defining the SAR of wollamide B by designing, synthesizing and testing their *in-vitro* antimycobacterial activities and optimizing its structure for better potency and pharmacokinetic properties.

## Result and discussion

### Synthesis of wollamide B

Before initiating the synthesis of a library of wollamide B analogues for SAR study, we synthesized the natural compound itself and evaluated its antimycobacterial activities against *Mtb* H37Rv.

**Fig 2:**
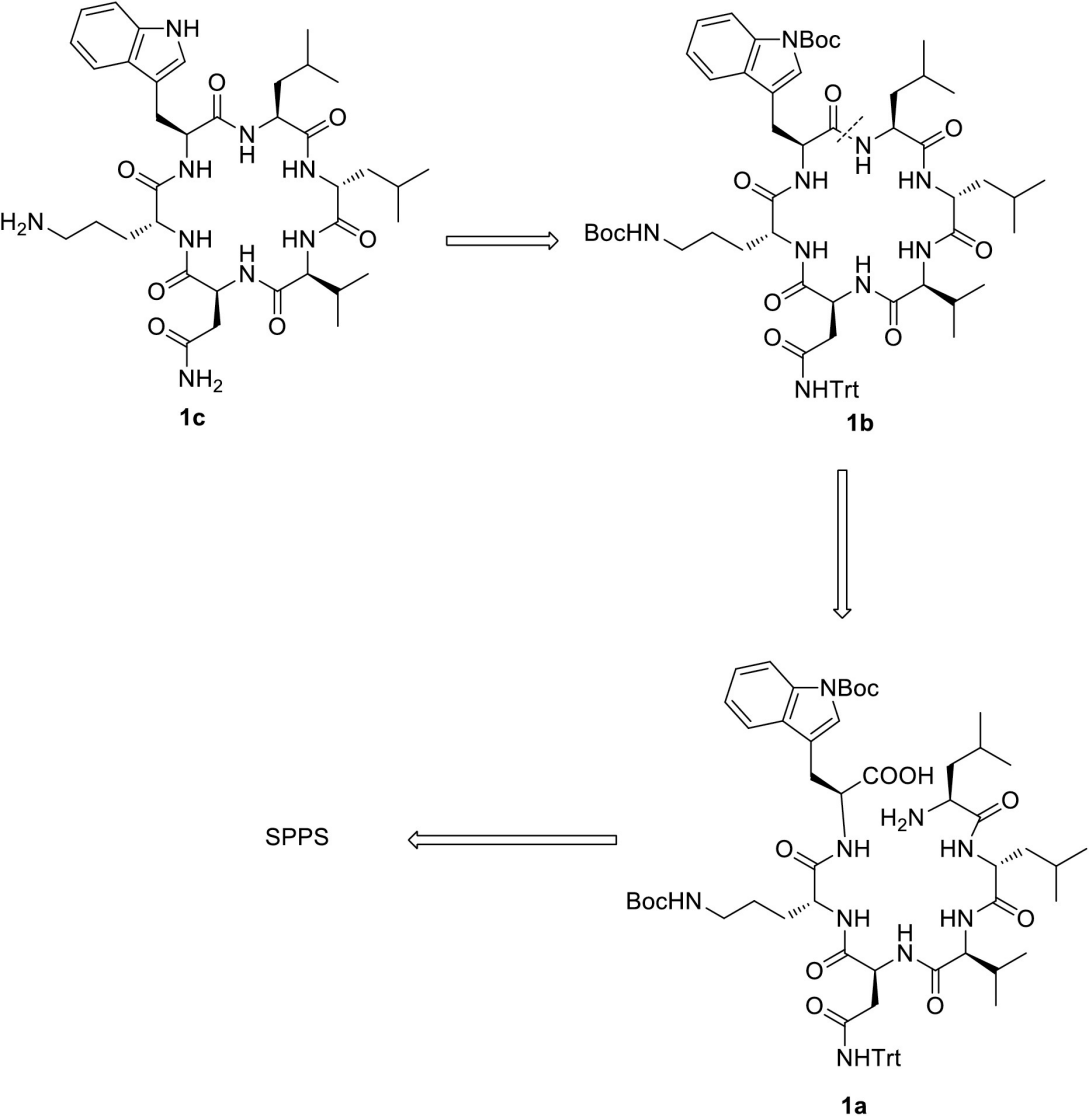
Scheme that shows the synthetic approach to wollamide B.

**Fig 3:**
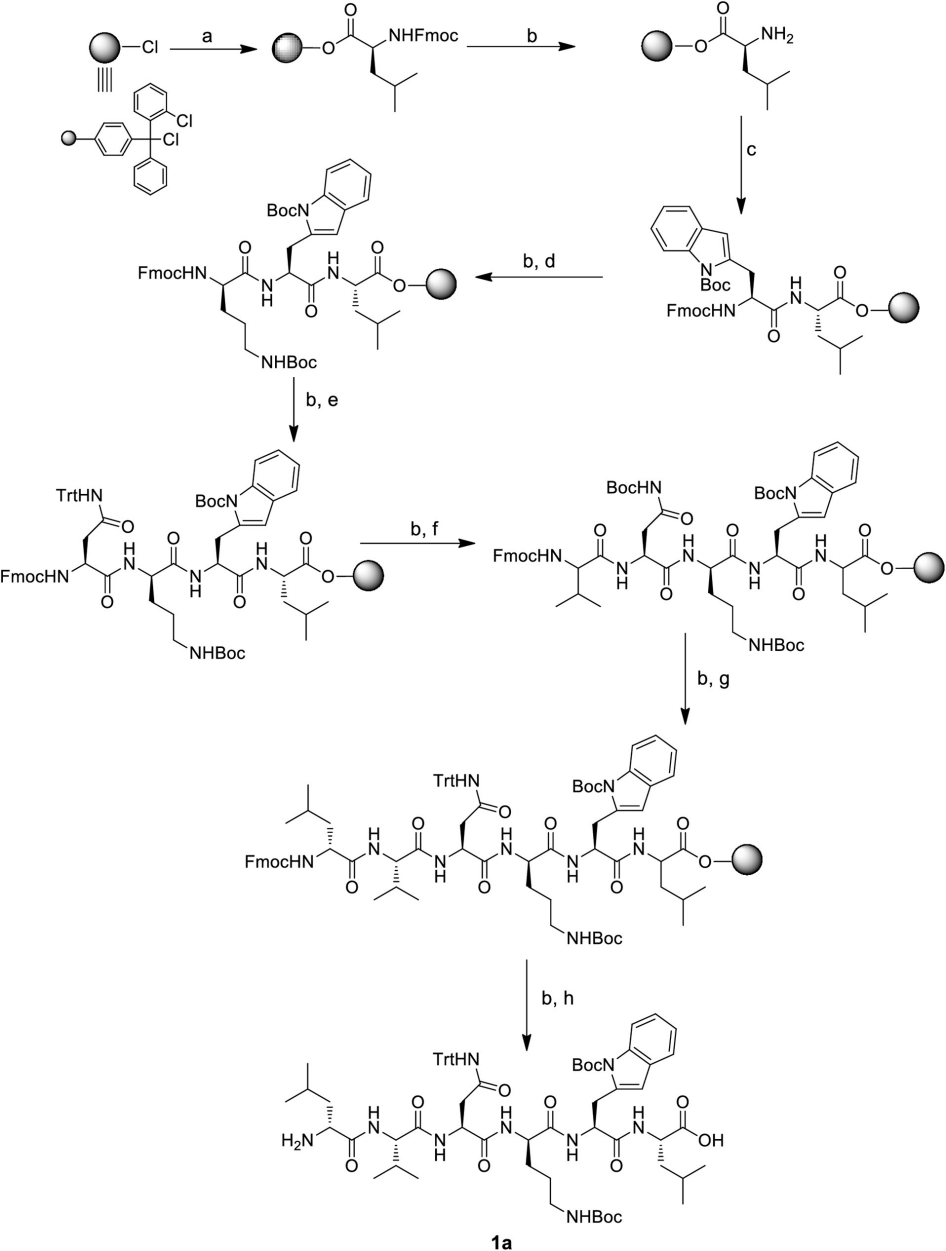
A synthetic scheme that shows the approach used to synthesize the linear precursor of wollamide B using SPPS. Reagents and conditions: a) Fmoc-L-Leu-OH, DIPEA, DCM, 2 h; b) 20% piperidine/DMF, 10 min (2X); c) Fmoc-L-Trp (Boc)-OH, HATU, DIPEA, NMP, 1 h; d) Fmoc-D-Orn (Boc)-OH, HATU, DIPEA, NMP, 1 h; e) Fmoc-L-Asn (Trt)-OH, HATU, DIPEA, NMP, 1 h; f) Fmoc-L-Val-OH, HATU, DIPEA, NMP, 1 h; g) Fmoc-D-Leu-OH, HATU, DIPEA, NMP, 1 h; h) 20% HFIP/DC, 1 h.

As the synthesis of wollamide B (**1c**) was not reported previously, we devise a new synthetic approach which is illustrated in Fig 2. We envisioned that **1c** would be available through deblocking of the orthogonally Boc protected cyclic precursor **1b** which in turn can be obtained through macrolactamization of a side chain protected linear hexapeptide precursor **1c** in solution phase. The hexapeptide **1c** would be obtained using Fmoc-based solid phase peptide synthesis (SPPS) approach according to the pathway outlined in Fig 3. The Boc/Trt-protecting groups were chosen as they can stand the mild acidic cleavage of the linear precursor using hexafluoroisopropanol (HFIP).

The synthesis of **1a** was initiated through loading the first amino acid, Fmoc-Leu-OH, on a 2-chlorotrityl chloride resin with DIPEA/DCM which was then followed by capping with methanol. Based on calculation of the increase in the mass of the resin after loading, the efficiency of loading was found to be 90%. Subsequent elongation of the peptides was achieved through stepwise coupling of the Fmoc-amino acids, namely Fmoc-Trp (Boc)-OH, Fmoc-D-Orn (Boc)-OH, Fmoc-Asn (Trt)-OH, Fmoc-Val-OH and Fmoc-D-Leu-OH by using HATU as the coupling agent in NMP-DIPEA. The deprotection of the Fmoc group from the resin bound peptide was always done using 20% piperidine in DMF prior to the next amide coupling process. Cleavage of the assembled linear precursors **1a** was achieved through treatment of the resin with 20% HFIP-DCM and did not affect the Boc and Trt protecting groups.

Macrocyclization of **1a** with HATU, HOBt and DIPEA in DMF under highly dilute conditions (1mM) gave crude **1b** which was purified by column chromatography. The purified cyclic peptide was treated with a cleaving cocktail that consisted of TFA-TIPS-H_2_O (95/2.5/2.5) to remove the Trt and Boc side chain protecting groups. This afforded **1c** after final purification, again by column chromatography. The purity of **1c** was asserted using LC-MS and the ^1^H, ^13^C NMR and ESI-MS spectral data were in accord to what was previously reported for the natural product [34].

### Antimycobacterial activity and *in vitro* ADME profiles of wollamide B

The antimycobacterial activity of **1c** was tested against the standard drug susceptible *Mtb* H37Rv strain using a microplate alamar blue broth dilution assay. The result of the testing (Table 1) showed that **1c** was active against the tested strain with an MIC of 0.6 μM.

**Table 1:** Antimycobacterial activities and *in vitro* ADME profiles of wollamide B (1c).

Activity/pharmacokinetics	Value
Antimycobacterial activities	
*Mtb* H37Rv MIC (μM)	0.6
*M*. *bovis* IC_50_ (μM) [34]	3.1
Cytotoxicity in Hep2G cells IC_50_(μM)	56
Solubility CLND^a^ (μg/mL)	151
Log D pH = 7.4	1.8
Artificial membrane permeability (nm/sec)	<3
% Human serum albumin binding	72.66
Plasma stability^b^ (% remaining)	
Mouse plasma	90
Human Plasma	100
*In vitro* microsomal clearance	
Mouse microsomal half-life (min)	>138.6
Mouse CL_int_ (mL/min/g)	<0.50
Mouse predicted % LBF	<16.83
Human microsomal half-life (min)	56.8
Human CL_int_ (mL/min/g)	0.97
Human predicted % LBF	56.88

^a^Refers to kinetic aqueous solubility determined by chemiluminescent nitrogen detection (CLND);

^b^Estimated by quantifying the amount of a compound remaining after incubation with human and mouse plasma for 2 h at 37°C; CL_int_, intrinsic clearance; LBF, liver blood flow.

An *in-vitro* ADME profiling was done for **1c** to evaluate its drug-likeness [35]. The ADME profiling included (1) a study of its physicochemical properties *vis-a-vis* water solubility, cell membrane permeability, human serum albumin binding affinity and lipophilicity, (2) determination of its plasma stability after 2 hour incubation of the compound with human and mouse plasma, and (3) metabolic stability, which was predicted from the experimentally measured intrinsic clearance of the compound in both mouse and human liver microsomes. The results are summarily displayed in Table 1, showing that **1c** has good aqueous solubility (151 μg/mL), moderate affinity towards plasma protein albumin (73%), modest lipophilicity (Log D = 1.8 at pH = 7.4) but poor passive permeability through artificial membranes (< 3 nm/sec). More than 90% of the compound remained intact after 2 hour incubation of the compound with mouse and human plasma at 37°C showing its remarkable *in vitro* plasma stability. The microsomal metabolic stability, expressed as % liver blood flow (LBF), was suitably high. Additionally **1c** did not show sign of toxicity up to a concentration of 50 μM against HepG2 cells (Table 1).

### Design and synthesis of wollamide B analogues

**Fig 4:**
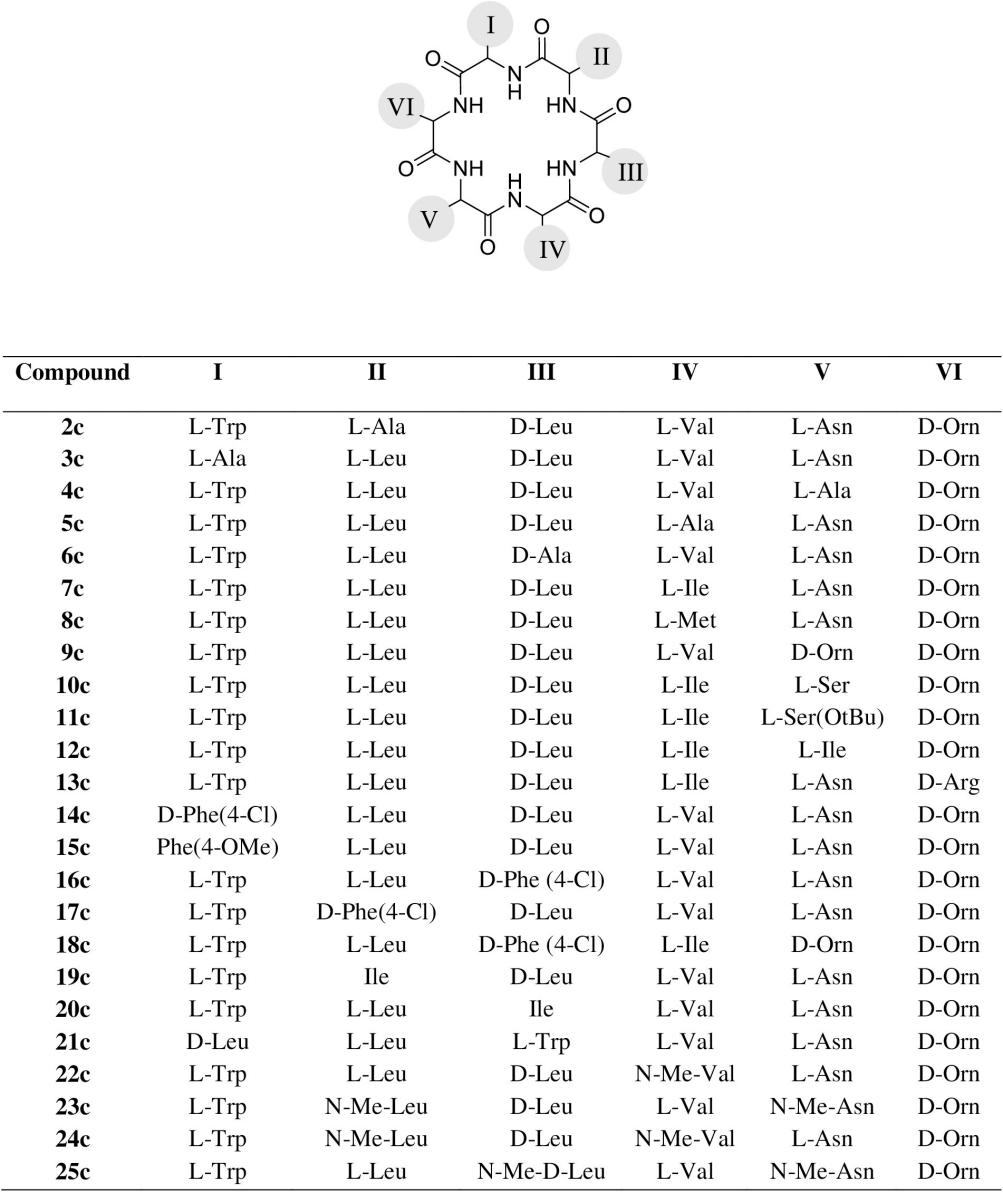
Structures of the synthesized wollamide B analogues.

The favorable *in vitro* activity and ADME properties of wollamide B laid the basis for rational optimization to achieve better antimycobacterial potency and pharmacokinetic properties. Accordingly, libraries of analogues were designed and synthesized (**2c** - **25c**) following the method described for the synthesis of wollamide B. The antimycobacterial activity testing and *in vitro* ADME profiling of the synthesized analogues was also done. The complete lists of all the newly prepared analogues are displayed in Fig 4. The results of the antimycobacterial activity and cytotoxicity assay, the *in vitro* experimentally determined physicochemical properties, plasma and metabolic stabilities of the synthesized analogues are presented in Tables 2–5.

**Table 2:** *In vitro* antimycobacterial MICs and cytotoxicities of wollamide B analogues.

Compound	MIC^a^ against *Mtb* H37Rv (μM)	IC_50_ against Hep2G Cells (μM)
**2c**	>80	>100
**3c**	>80	>100
**4c**	20	>100
**5c**	>80	>100
**6c**	>80	>100
**7c**	1.1	50
**8c**	>80	nd
**9c**	2.5	>100
**10c**	15	>100
**11c**	>40	>100
**12c**	3.1	>100
**13c**	0.6	nd
**14c**	40	>100
**15c**	>80	nd
**16c**	1.9	60
**17c**	30	>100
**18c**	17.5	>100
**19c**	7.5	>100
**20c**	>80	>100
**21c**	20	>100
**22c**	12.5	nd
**23c**	>80	>100
**24c**	>80	>100
**25c**	>80	>100

^a^INH was used as a positive control and each test was done as duplicate (see the method part); nd = not determined.

**Table 3:** The *in vitro* physicochemical properties of the synthesized wollamide B analogues.

Compound	Solubility^a^ (μg/mL)	% HSA	Log D	Permeability^b^ (nm/sec)
**2c**	>134	43.14	0.94	<10
**3c**	136	25.51	nd	nd
**4c**	143	82.76	2	<3
**5c**	129	56.76	1.32	<10
**6c**	≥196	42.21	1.04	<10
**7c**	≥177	76.35	1.83	<10
**8c**	≥164	73.79	1.78	<10
**9c**	39	86.46	1.32	10
**10c**	≥228	86.47	2.16	<10
**11c**	202	92.68	3.00	<3
**12c**	≥206	96.06	2.94	19
**13c**	≥189	87.55	2.05	11
**14c**	≥218	70.25	1.9	<10
**15c**	≥189	46.55	1.73	<10
**16c**	≥192	92.54	1.92	<3
**17c**	≥230	93.83	2.12	17
**18c**	7	98.70	nd	80
**19c**	≥183	67.10	1.73	<10
**20c**	≥195	83.67	1.83	<3
**21c**	≥210	79.64	1.89	<3
**22c**	≥153	73.98	1.96	<10
**23c**	≥174	82.21	2.00	<10
**24c**	≥217	87.02	2.18	<10
**25c**	≥221	82.97	2.25	<10

^a^Refers to kinetic aqueous solubility determined by chemiluminescent nitrogen detection (CLND);

^b^refers to passive artificial membrane permeability; HSA, human serum albumin binding; nd, not determined.

**Table 4:** *In vitro* plasma stabilities for selected wollamides B analogues.

Compound	Plasma stability (% remaining)*	
	Human	Mouse
**2c**	107	93
**3c**	86	92
**4c**	95	100
**5c**	98	96
**6c**	95	102
**7c**	99	98
**9c**	100	100
**14c**	104	70
**16c**	99	98
**21c**	97	98

*Estimated by quantifying the amount of a compound remaining after incubation with human and mouse plasma for 2 hours at 37°C. Each test were done in triplicates (see method part)

**Table 5:** Microsomal stabilities of wollamide B analogues.

Compound	CL_int_ (mL/min/g tissue)		CL_pred_ (mL/min/kg)		% LBF		Microsomal t_1/2_ (min)	
	Human	Mouse	Human	Mouse	Human	Mouse	Human	Mouse
**2c**	<0.40	<0.48	6.31	<20.50	<35.08	<16.27	>138.60	>138.60
**3c**	<0.40	<0.48	6.31	<20.50	<35.08	<16.27	>138.60	>138.60
**4c**	<0.40	<0.48	6.31	<20.50	<35.08	<16.27	>138.60	>138.60
**5c**	<0.40	<0.48	6.31	<20.50	<35.08	<16.27	>138.60	>138.60
**6c**	0.73	<0.48	8.99	<20.50	49.94	<16.27	75.10	>138.60
**7c**	1.70	<0.48	12.57	<20.50	69.81	<16.27	32.38	>138.60
**8c**	1.79	<0.48	12.76	<35.08	70.90	<16.30	30.66	>138.60
**9c**	0.45	0.57	6.85	23.53	38.08	18.68	121.79	117.25
**10c**	9.77	<0.48	16.70	<20.50	93.00	<16.3	5.63	>138.60
**11c**	3.33	<0.48	14.70	<20.50	81.90	<16.3	16.54	>138.60
**12c**	5.93	<0.48	16.02	<20.50	88.98	<16.39	9.28	>138.60
**13c**	7.44	1.01	16.38	36.60	91.01	29.00	7.40	63.00
**14c**	<0.40	<0.48	6.31	<20.50	<35.08	<16.27	>138.60	>138.60
**15c**	<0.40	0.78	<6.35	30.20	<35.08	24.00	>138.60	85.50
**16c**	2.43	0.59	13.82	24.21	76.78	19.21	22.64	113.23
**17c**	<0.40	0.54	<6.35	22.60	<35.08	17.9	>138.60	123.50
**18c**	1.06	0.98	10.65	35.80	59.15	28.4	51.72	67.90
**19c**	<0.40	<0.48	<6.35	<20.50	<35.08	<16.3	>138.60	>138.60
**20c**	<0.40	<0.48	<6.35	<20.50	<35.08	<16.3	>138.60	>138.60
**21c**	<0.40	<0.50	<6.35	<21.21	<35.25	<16.83	>138.60	>138.60
**22c**	<0.40	<0.48	<6.35	<20.50	<35.08	<16.3	>138.60	>138.60
**23c**	3.19	1.06	14.63	37.80	81.29	30.00	17.24	63.00
**24c**	3.51	1.79	14.90	52.90	82.70	42.0	37.26	15.68
**25c**	<0.40	<0.48	<6.35	<20.50	<35.08	<16.3	>138.60	>138.60

Cl_int_, intrinsic microsomal clearance (see in the method); CL_pred_, predicted metabolic clearance; LBF, liver blood flow; nd, not determined.

As presently there is no information about the target and mode of action of wollamides, the design of the analogues was based on an approach commonly utilized to generate SAR of biologically active antimicrobial peptides.

#### Analogues synthesized through Ala scanning

The first generation of wollamide derivatives was synthesized by sequentially replacing each of the amino acids of wollamide B, except the D-Orn, with Ala (alanine scanning). This was done to assess the contribution of each amino acid of the lead for its antimycobacterial activity [36]. The D-Orn moiety was not replaced as the respective wollamide B analogue desotamide B had also been isolated and shown no antimycobacterial activity [34]. Accordingly, five Ala scan analogues (2c-6c) were synthesized. The result of the *in vitro* atimycobacterial testing showed that all of them were inactive at the maximum dose tested except 4c which showed moderate activity with an MIC of 20 μM. This result infers that the side chains of all the amino acids present in the molecule contribute to activity either indirectly through dictating conformational space [37] or directly interacting with an assumed target.

The *in vitro* plasma stability of all Ala scan derivatives was similar to wollamide B, i.e. more than 86% of all the compounds remained intact after 2 hours incubation with human and mouse plasma at 37°C (Table 4). This is another proof of the excellent stability of the class in general.

The Ala scan analogues further shared higher metabolic stability and longer half-life compared to wollamide B. Based on the predicted hepatic clearance values, while wollamide B exhibited moderate metabolic stability (% LBF = 1/2), the Ala scan analogues were found in the range which is considered to be metabolically stable (% LBF < 1/3). Moreover, the microsomal half-lives of the Ala scan derivatives were doubled compared to wollamide B.

The experimentally determined log D values ranged between 0.94 (**2c**) and 2.00 (**4c**). Considering their molecular weight, this value is not satisfactory for good cellular permeability [38]. Hence, further structural optimization of wollamide B should focus on improving this issue. The experimentally determined serum albumin binding affinity of the compounds showed large variation ranging from 26% for **3c** to 83% for **4c**. This value seems correlated with the degree of lipophilicity and the presence or absence of the aromatic indole moiety. When the compound was more lipophilic and the indole moiety present, the binding affinity was found to be high. On the other hand, replacing Trp with Ala (**3c**) significantly lowered the affinity showing the contribution of the aromatic side chain for the relatively high albumin binding affinity of wollamide B.

We then closely examined the role of each of the six amino acids of wollamide B in view of improving potency and activity. Consequently, more analogues (second generation) were synthesized by replacing each residue with other proteinogenic and non proteinogenic amino acids.

#### Analogues synthesized through replacing the amino acid Val

Val could be a good site for variation because wollamide A (another naturally occurring wollamide) which have another amino acid at this site (*allo*-Ile) was also found to be active against *M*. *bovis* (2.8 μM)[34]. Val was replaced by other natural lipophilic amino acids namely, Ile (7c) and Met (8c). Ile was chosen to test the possibility of changing the expensive *allo*-Ile group of wollamide A while keeping its activity. Ile leads to an increase in the hydrophobicity of antimicrobial peptides by partly shielding its own NH group [39].

Indeed **7c** exhibited potent antimycobacterial activity with an MIC of 1.1 μM against *Mtb* H37Rv, and its Log D value was slightly increased compared to wollamide B. However, this modification was found to negatively affect the metabolic clearance in human liver microsomes (Table 5).

Exchanging Val by the lipophilic Met (**8c**) resulted in complete loss of activity at the maximum dose tested (80 μM) though the compound had comparable lipophilicity. This finding, together with a similar result found by replacing Val with Ala, indicates that the Val/Ile moieties have more specific roles than simply maintaining lipophilicity at this site.

#### Analogues synthesized through replacing the amino acid Asn

Amphiphilicity and cationicity are the two important features of AMPs that influence their activity [40]. While a certain degree of cationicity is essential for initial selective binding to the negatively charged cell surfaces of prokaryotes, the amphiphilic nature enables them to interfere with the cytoplasmic membrane barrier or to self-promote uptake across the cellular membrane [41, 42]. These two parameters were also showed to be crucial for activity in another series of antimycobacterial peptides [12]. Based on such findings, analogues with varying cationicity and amphiphilicity were designed and synthesized.

To testify the effect of introducing a second positive charge on activity and stability, an analogue was synthesized by introducing another D-Orn in exchange for Asn (**9c**). The substitution was done in place of the polar amino acid Asn not to greatly compromise the overall lipophilicity. Moreover, keeping the two D-Orn moieties adjacent to each other helps to maintain the amphiphilic nature.

This modification yielded a compound with an appreciable antimycobacterial activity (MIC of 2.5 μM). Of greater significance, compound **9c** exhibited a better *in vitro* microsomal stability (% LBF < 1/3) and its half-life was two times greater than that of the lead compound. This finding showed the possibility of improving the microsomal stability in this class while still keeping high antimycobacterial activity.

Asn was also replaced with the polar amino acid Ser (**10c**) and the lipophilic Ser (t-Bu) (**11c**) to explore the effect of varying the polarity of this position. While **10c** retained moderate antimycobacterial activity with an MIC of 15 μM, compound **11c** was completely inactive.

The result of the *in vitro* ADME profiling of compound **10c** showed that such modification did not affect the overall lipophilicity of wollamide B. However, it had an adverse effect on the metabolic stability. Among all the synthesized analogues, **10C** exhibited the lowest stability in human liver microsomes.

In view of improving the poor lipophilicity and permeability of the class in general, compound **12c** was prepared through exchanging both Asn and Val of the lead with the lipophilic amino acid Ile. While keeping significant antimycobacterial potency (MIC of 3.1 μM), this change indeed produced the most lipophilic analogue so far. While **12c** also exhibited better permeability than any of the synthesized analogues, it had high affinity to plasma proteins and a short microsomal half-life. This finding showed the possibility of improving passive membrane stability in this class while still keeping meaningful high antimycobacterial activity. However, more work has to be done to find a substituent that conveys the right balance of potency, metabolic stability and permeability. The other possibility is to try to optimize just two parameters, e.g. potency and metabolic stability or potency and membrane permeability, or perhaps combining a potent cyclopeptide—if it is a CYP450 substrate—with a CYP inhibitor.

#### Analogues synthesized through replacing the amino acid D-Orn

Previous studies done on antimicrobial peptides showed that Arg containing peptides are generally more potent than the corresponding Lys or Orn analogues [43]. The higher antimicrobial activities of such peptides is attributed to the guanidinium group of Arg which allows more dispersed positive charge and offers greater directionality and possibility of hydrogen bonding with the surrounding water molecules. The Arg side chain allows the formation of more interactions compared to the mono charge present in ornithine [44, 45].

As we suspected introduction of the strongly basic guanidine side chain would greatly compromise lipophilicity, Val was simultaneously replaced by Ile. This afforded a very potent analogue (**13c**) with an MIC of 0.6 μM. On top of its remarkable activity, compound **13c** exhibited higher lipophilicity (Log D = 2.05), permeability (11 nm/sec) and aqueous solubility (189 μg/mL) than wollamide B. The major shortcoming of this compound was its poor metabolic stability in human liver microsomes which we associated with the metabolically labile guanidine group [46].

#### Analogues synthesized through replacing the amino acids Trp, Leu and D-Leu

Another constitutional parameter for activity that we investigated was aromaticity. The number, type and arrangement of aromatic amino residues of antimicrobial peptides was shown to have an influence on their activities [43]. To testify if this also holds for wollamide B, we varied the type, number and arrangement of aromatic amino acids in the molecule.

Trp was swapped for Phe where the para positions of the phenyl side chain were blocked either with a chlorine (**14c**) or hydroxymethyl group (**15c**). The *in vitro* metabolic study of these two compounds showed that the predicted metabolic clearances of both compounds were much lower than wollamide B. The result of the *in vitro* ADME profiling also revealed that the two compounds exhibited nearly the same degree of lipophilicity with the lead. However, the antimycobacterial activities of **14c** and **15c** were much lower. While **14c** showed only weak activity at an MIC of 40 μM, **15c** was completely inactive at the maximum dose tested. The loss of activities, in spite of maintaining comparable lipophilicity and greater metabolic stability, suggests that the role of Trp is far beyond contributing to the hydrophobicity of the molecule. This finding goes in line with a study made to examine the role of Trp for the antimicrobial activity Arg and Trp rich peptides [44, 47, 48]. The study highlighted the unique properties of Trp to have a distinct preference for the interfacial region of lipid bilayers and its ability to participate in cation–π interactions, thereby facilitating enhanced peptide–membrane interactions.

Two analogues were synthesized by introducing additional aromatic amino acids in the molecule, namely 4-chloro-D-Phe in place of both D-Leu (**16c**) and Leu (**17c**). This was done to check the effect of having multiple aromatic amino acids with different arrangement in the molecule. While **16c** still maintained a notably high antimycobacterial activity **(**MIC = 1.9 μM), the MIC of **17c** was 30 μM which is much lower than the lead. Contrary to its good antimycobacterial potency, **16c** exhibited unfavorable pharmacokinetic properties with poor metabolic stability (% LBF > ¾), short half-life (< 30 min) and high serum albumin binding (92%). The drop in the activity of **17c**, in spite of its better metabolic stability, might be attributed to the change in the stereochemistry of the amino acid from L to D, again suggesting that a specific interaction with a yet unknown target or targets causes activity in this cyclopeptide class.

Aiming at improving the poor *in vitro* metabolic stabilities of **16c**, **18c** was synthesized by incorporating a D-Orn in place Asn. This was based on the finding in compound **9c** where such change improved metabolic stability. Though the desired improvement in metabolic stability was achieved, the antimycobacterial activity was greatly compromised (MIC = 17.5 μM).

Both the D- and L-Leu residues of wollamide B were further replaced with Ile to check if such modification had similar outcome like the one obtained in compound **7c**. While replacing L-Leu with Ile (**19c**) retained moderate activity (MIC = 7.5 μM) exchanging D-Leu for L-Ile (**20c**) completely abolished the activity. This was another instance that switching the stereocenters of these two amino acids gravely affects the activity of wollamide B. The predicted metabolic stabilities and the half-lives of both **19c** and **20c** were much better than the lead compound. This is particularly interesting for **19c** as it still retained some antimycobacterial activity.

To investigate the effect of changing the sequence of the amino acids in wollamide B, one of its stereoisomers (**21c**) was prepared through randomly exchanging the position of Trp for D-Leu. This compound exhibited different stability and physicochemical properties. While the change improved the experimental *in vitro* pharmacokinetic properties through increasing aqueous solubility, metabolic stability and half-life, the antimycobacterial activity was compromised. The compound showed activity against *Mtb* H37Rv with an MIC of 20 μM. This finding indicates that the amino acids sequence is indeed crucial both for antimycobacterial activity and pharmacokinetic properties of wollamides.

#### N-methylated wollamides

Many studies showed N-methylation of cyclic peptides to offer a number of advantages, including (1) improvement of the therapeutic efficacy of peptides by fine-tuning their selectivity for a receptor [49], (2) enhancing the hydrophobicity by reducing the number of hydrogen-bond donors and by preventing the formation of intermolecular and intramolecular hydrogen bonds which in turn improves oral bioavailability [50]. (3) Multiple *N*-methylations also introduce remarkable metabolic stability, even in the presence of gut peptidases, in comparison with cyclization only [51]. Hence, hoping to achieve one or more of the aforementioned goals, *mono*-, and *di—*N-methylated analogues were synthesized (22c-25c) and checked for their *in vitro* antimycobacterial activities, stabilities and physicochemical properties.

The effect of N-methylation on the activity of the prepared analogues produced varying results. While the *mono*-N-methylated analogue (**22c**) retained moderate antimycobacterial activity (MIC = 12.5 μM), the *di*-N-methylated analogues (**23c-25c**) were completely inactive irrespective of the position of the two methyl groups in the molecule.

As expected, N-methylation of the Val moiety of wollamide B increased lipophilicity and human liver microsomal stability. However, the contribution of this modification in improving the low permeability was not significant.

N, N-Dimethylated analogues displayed varying stability in human liver microsomes depending on the site of methylation. The metabolic stability was found to be compromised when the two methyl groups were introduced at Leu and Val (leading to **23c**) and on Leu and Asn (leading to **24c**). On the contrary, dimethylating the D-Leu and Asn to give **25c** increased microsomal stability.

The determined Log D values of the N-dimethylated analogues were also not identical, in spite of the introduction of the same functional groups. While **25c** exhibited the highest Log D value in these series (2.25), **23c** was found to be only slightly more lipophilic than its *mono*-N-methylated analogue (Log D = 2.0). Such differences in lipophilicity may be due to differences in the number of exposed polar surfaces because the molecules assume different conformations as a result of such methylations.

## Conclusion

In conclusion, the reported chemical structure and antimycobacterial activity of wollamide B was verified by its independent synthesis and testing its activity against *Mtb* H37Rv. The SAR study that was performed based on alanine scanning revealed that all amino acids of this cyclohexapeptide contribute to the observed activity. The structural modifications reported here show what to change in view of improved potency and pharmacokinetic properties. Among the synthesized 25 wollamides, five (**7c**, **9c**, **10c**, **13c** and **16c**) showed antimycobacterial potency with an MIC ≤ 3.1 μM. Compounds **13c** (MIC = 0.6 μM) and **7c** (MIC = 1.1 μM) displayed a good balance of antimycobacterial activity and pharmacokinetic properties, deserving further studies. The evaluation of their *in vivo* efficacy is presently under way.

## Materials and methods

### General

All the chemicals and reagents used for synthesis and purification were purchased from a commercial source and used without further purification. A polypropylene syringe (Inject®, B.Braun Melsungen AG, Germany) fitted with a frits column plate was used for the solid phase synthesis of the linear hexapeptides. For a solution phase synthesis, a reaction progress was monitored by analytical thin layer chromatography (TLC) (Silica gel 60 F_254_, Merk, Germany) and the purification of all the synthesized compounds were performed by a column chromatography using Silica gel 60 (0.063–0.200mm, Merk) as a stationary phase. TLC plates were visualized by 254 nm UV light. Electrospray ionization mass spectra (ESI-MS) were acquired using SSQ 710 C mass Spektrometer, Finningen MAT GmbH, Bremen, Germany. NMR spectra were recorded on Bruker Varian Inova Unity 500 (operating frequency 300 or 500 MHz for ^1^H-NMR and 101 or 126 MHz for ^13^C-NMR), Kolthoff GmbH, Filsum, Germany. Proton and carbon chemical shifts are reported in ppm. ^1^H NMR spectral data are reported as follows: chemical shift, multiplicity (s = singlet, d = doublet, t = triplet, q = quartet, m = multiplet, br = broad, ovlp = overlapping), coupling constant in Hz and integration.

Purity analysis of all the final target compounds was done by an independent quality control team at GlaxoSmithKline (GSK), Stevenage using UPLC-MS with UV diode array detection and MS used to confirm identity. Evaporative light scattering detector (ELSD) was also used as an additional method of detection to confirm if there are nonechromophore impurities. Generally the purity of the synthesized final compounds was found ≥ 95%. UPLC-MS involved the following: Waters Acquity UPLC system coupled to a Waters TQD ESI mass spectrometer and Waters TUV detector. A Waters Acquity UPLC BEH C18 1.7 μm, 2.1 mm × 50 mm column was used. Solvent A consisted of water with 0.1% formic acid. Solvent B consisted of acetonitrile with 0.1% formic acid. The method involved gradient elution as follows: flow 1.0 mL/min, the first 1.5 min (97% A, 3% B), till 1.9 min (from 97% A, 3% B to 100% B, 0% A), then for the last 0.4 min (95% B, 5% A). The wavelength for UV detection was 254 nm.

### Synthesis of the target cyclic hexapeptides

Synthesis of all the target compounds were achieved in three steps which included construction of the linear hexapeptide precursors using a solid support; macrocylclization of the formed linear precursors in solution phase and global deprotection of all the reactive amino acid side chains [52].

### Synthesis of the linear hexapeptide precursors

The linear hexapeptides (**1a** - **25a**) (Table A in S1 File) were synthesized following a standard Fmoc based solid phase peptide synthesis (SPPS) approach using 2-chlorotrityl chloride resin as a solid support as described by Chatterjee J et al. [52].

The synthesis were initiated through loading the first amino acids, Fmoc-XX-OH (1.2 eq.), on a 2-chlorotrityl chloride resin (1.6 mmol/g loading capacity) with DIPEA (2.5 eq.)/DCM which was followed by capping with methanol. Subsequent elongation of the peptides was achieved through stepwise coupling of the Fmoc-amino acids using HATU (3.eq)/DIPEA (6 eq.) as the coupling agent in NMP. The deprotection of the Fmoc group from the resin bound peptide was always done using 20% piperidine in DMF prior to the next amide coupling. Cleavage of the assembled linear precursors was achieved through treatment of the resin with 20% HFIP-DCM.

### Macrocyclization

The linear hexapeptide precursors prepared by SPPS approach were dissolved in DMF (1mM) and cooled to 0°C in an ice bath. To the solution was added HATU (3 eq.), and HOBt (3 eq.) under vigorous stirring. DIPEA (10 eq.) was then added to the dilute solution and the reaction mixture was allowed to warm slowly to the room temperature and kept stirring for 3 days. Following these, the solvent was removed under reduced pressure and the obtained crude solid mass was purified by a column chromatography after doing the relevant work up (see the supplementary material). This afforded pure cyclic hexapeptides (**1b** – **25b**) (Table B in S1 File) whose reactive amino acid side chains were protected.

### Global deprotection of the side chains protecting groups

The protecting groups from reactive amino acid side chains of the cyclic hexapeptides were removed with a cleaving cocktail made up of TFA/TIPS/H2O (95:2.5/2.5/) for amino acids that contain Trt/Pbf protecting groups and with TFA/DCM (1:1) for amino acids containing Boc/tBu protecting groups. The cleaving solution was then removed under a strong pressure and the residue was purified by column chromatography.

The structures of the final compounds were confirmed by a combination of ESI-MS and NMR spectral analysis and their purity were asserted using UPLC-MS.

### *Mtb* inhibition assay

The measurement of the MIC for each tested compound was performed in 96-well flat-bottomed, polystyrene microtiter plates. Ten 2-fold drug dilutions were performed in neat DMSO. Then 5 μL of these drug solutions were added to 95 μL of Middlebrook 7H9 medium (Difco catalogue ref 271310). Isoniazid was used as the positive control: eight 2-fold dilutions of isoniazid were prepared, starting at 160 μg/mL, and 5 μL of this control curve was added to 95 μL of Middlebrook 7H9 medium. Likewise, 5 μL of neat DMSO were used as the growth and blank controls. The inoculum was standardized to approximately 1 × 10^7^ CFU/mL and diluted 1 in 100 in Middlebrook 7H9 broth (10% ADC; Becton Dickinson BBL catalogue ref. 211887) and 0.025% Tween 80, to produce the final inoculum. Following this, 100 μL of this inoculum was added to the entire plate, except for the blank controls. All of the plates were placed in a sealed box to prevent drying out of the peripheral wells, and they were incubated at 37°C without shaking for 6 days. A resazurin solution was prepared by dissolving one tablet of resazurin (Resazurin Tablets for Milk Testing; ref 330884Y, VWR International Ltd.) in 30 mL of sterile phosphate-buffered saline. Of this solution, 25 μL was added to each well. Fluorescence was measured (Spectramax M5, Molecular Devices, excitation 530 nm, emission 590 nm) after 48 h, to determine the MIC values. For each compound, the average value of the duplicate samples was calculated.

### HepG2 cytotoxicity assay

Actively growing HepG2 cells were removed from a T-175 TC flask using 5 mL Eagle’s MEM (containing 10% FBS, 1% NEAA, 1% penicillin/streptomycin) and dispersed in the medium by repeated pipetting. Seeding density was checked to ensure that new monolayers were not >50% confluent at the time of harvesting. Cell suspension was added to 500 mL of the same medium at a final density of 1.2x10^5^ cells/ per mL. This cell suspension (25 μL, typically 3000 cells per well) was dispensed into the wells of 384-well clear-bottom plates (Greiner, cat. # 781091) using a Multidrop instrument. Prior to addition of the cell suspension, the screening compounds (250 nL) were dispensed into the plates with an Echo 555 instrument. Plates were allowed to incubate at 37°C at 80% relative humidity for 48 h under 5% CO_2_. After the incubation period, the plates were allowed to equilibrate at room temperature for 30 min before proceeding to develop the luminescent signal. The signal developer, CellTiter-Glo (Promega) was equilibrated at room temperature for 30 min and added to the plates (25 μL per well) using a Multidrop. The plates were left for 10min at room temperature for stabilization and were subsequently read using a View Lux instrument (PerkinElmer). The human biological samples were sourced ethically and their research use was in accord with the terms of the informed consents. The use of human biological samples was approved by human biological samples management (HBSM) core team of GSK and fulfils HBSM GSK policies.

### Physicochemical properties

#### Chemi-Luminescent Nitrogen Detection (CLND) solubility assay

GSK in-house kinetic solubility assay: 5 μL of 10mM DMSO stock solution diluted to 100 uL with pH7.4 phosphate buffered saline, equilibrated for 1 hour at room temperature, filtered through Millipore Multiscreen HTS-PCF filter plates (MSSL BPC).

The filtrate is quantified by suitably calibrated flow injection CLND. The standard error of the CLND solubility determination is ±30 μM, the upper limit of the solubility is 500 μM when working from 10 mM DMSO stock solution.

#### Chrom logD assay

The Chromatographic Hydrophobicity Index (CHI) values were measured using a reversed phase HPLC column (50 x 2 mmx 3 μMGemini NX C18, Phenomenex, UK) with fast acetonitrile gradient at starting mobile phase of 100% pH = 7.4 buffer. CHI values are derived directly from the gradient retention times by using a calibration line obtained for standard compounds. The CHI value approximates to the volume % organic concentration when the compound elutes. CHI is linearly transformed into ChromlogD by the formula:

ChromlogD=0.0857CHI−2.00

The average error of the assay is ±3 CHI unit or ±0.25 ChromlogD.

#### Artificial membrane permeability assay

A 8% L-a-phosphatidylcholine (EPC) in 1% cholesterol decane solution and a 1.8% EPC in cholesterol decane solution were prepared. The lipid solution was then aliquoted into 4 mL capped vials, sealed with parafilm and stored in-20°Cfreezer. The lipid solution was then transferred from 4 mL vial into 96-well half area plate (130μL/well) for daily assay usage. An additional 50 mM phosphate buffer with 0.5% encapsin, pH at 7.4 was prepared. The assay was run by the Biomek FX and Biomek software. The assay procedure is written under the Biomek software. For one batch assay, it can test two 96-well sample plates with at least one standard on each sample plate. The total assay time was about 4 hours. 3.5 μL of lipid solution were added to the filler plate, shaken for 12 seconds, and 250 μL of buffer were added to donor side and 100 μL to the receiver side. The assay plate was shaken for 45 min before adding the compounds. The test compounds (2.5 μL) were added to the donor side. The assay was run as replicates: Assay plates1 and 2 tested the sample plate 1; assay plates 3 and 4 tested the sample plate 2. The assay plates were then incubated and shaken for 3 hours at room temperature.

The assay samples were transferred to the HPLC analysis plates and 100 μL of receiver solution were aspirated and transferred to the receiver for analysis. Similarly, another 100 μL from the donor solution were transferred to the donor analysis plate.

Compound concentration was measured by HPLC at different time-points and permeability was established as nm/sec.

#### Human serum albumin (HSA) binding assay

HSA binding assay was performed according to the method described by Valko K, et al [53]. The basic principle of the measurements is the determination of gradient retention times of compounds on immobilized HSA column and converting these retention times to the appropriate property values using calibration. The peaks are detected and identified by UV. Calibration sets of compounds are measured before each run for which the HSA binding values are known. The retention times of the calibration set of compounds are plotted against these known values. The slope and the intercept of the obtained straight lines are used to calculate the HSA binding for the unknown compounds.

#### Plasma stability assay

Plasma stability of the compounds was assessed in CD1 mouse and human plasma. Plasma was spiked with 1 μL of a 10 mM of each test compound solution to produce a 1 uM incubation. Three separate 300 μl aliquots were then taken from each tube and incubated at 37°C for 2 h. At each time point (0, 15, 30, 60 and 120 min), 50 μL of plasma were collected from each sample. Samples were extracted by protein precipitation with 250 μL of 0.1% AcOH acetonitrile methanol 3–1 (v/v) containing 1uM internal standard and centrifuged for 10min at 2800rpm. Supernatants were collected prior the injection onto an LC-MS/MS system. Analyte/Internal standard peak area ratios were referenced to the zero time-point samples as 100% in order to determine the percentage of compound remaining for each time-point. Ln plots of the % remaining for each compound were used to determine the half-life for the plasma incubations.

The human biological samples were sourced ethically, and their research use was in accord with the terms of the informed consents. The use of human biological samples was approved by human biological samples management (HBSM) core team of GSK and fulfils HBSM GSK policies.

#### Intrinsic clearance (CL_int_) assay

Pooled mouse and human liver microsomes are purchased from Xenotech. Microsomes (final protein concentration 0.5 mg/mL, MgCl2 (final concentration = 5 mM) and test compound (final substrate concentration = 0.5] M; final DMSO concentration = 0.5%) in 0.1 M phosphate buffer pH 7.4 are pre-incubated at 37°C prior to the addition of NADPH (final concentration = 1 mM) to initiate the reaction. The final incubation volume is 600 μL.

Control incubation is included for each compound tested where 0.1 M phosphate buffer pH 7.4 is added instead of NADPH (minus NADPH). One control compound is included with each species. All incubations are performed singularly for each test compound. Each compound is incubated for 45 minutes and samples (100 μL) of incubate are taken at 0, 5, 15, 30 and 45 min. The control (minus NADPH) is sampled at 30 min only. The reactions are stopped by the addition of sample to 200 μL Acetonitrile: Methanol 3:1 containing internal standard. The terminated samples are centrifuged at 2,500 rpm for 20 min at 4°C to precipitate the protein. Quantitative analysis: following protein precipitation, the samples were analyzed using specific LC-MS/MS conditions. Data analysis: from a plot of ln peak area ratio (compound peak area/internal standard peak area) against time, the gradient of the line was determined. Subsequently, half-life and intrinsic clearance were calculated using the equations below:

Eliminationrateconstant(K)=(−gradient)

$$\mathrm{Half}\;\mathrm{life}\;(t_{1/2})\;(\min)=\frac{0.693}{K}$$

$$\mathrm{Cli}\;(\mathrm{mL}/\min/g\;\mathrm{protien})=\frac{V\times 0.693}{t_{1/2}}$$

WhereV=Incubationvolume(mL/gmicrosomalprotien)

## Supporting information

S1 File Supplementary text, tables and figures. (PDF)

## Abbreviations

AMP: antimycobacterial Peptide
Boc-: *tert*-butyloxycarbonyl
CHI: chromatographic hydrophobicity index
CL_int_: intrinsic microsomal clearance
CLND: chemiluminescent nitrogen detection
DCM: dichloromethane
DIPEA, N: N-Diisopropylethylamine
DMF: dimethylformamide
ADME: drug metabolism and pharmacokinetics
DMSO: dimethyl sulfoxide
Fmoc-: fluorenylmethyloxycarbonyl
HATU: 1-[Bis(dimethylamino)methylene]-1*H*-1,2,3-triazolo[4,5-*b*]pyridinium 3-oxid hexafluorophosphate
HFIP: hexafluoro-2-propanol
HOBt: hydroxybenzotriazole
IC_50_: half maximal inhibitory concentration
LBF: liver blood flow
MIC: minimum inhibitory concentration
Mtb: *Mycobacterium. Tuberculosis*
NADPH: reduced nicotinamide adenine dinucleotide phosphate
NMP: N-Methyl-2-pyrrolidone
Pbf-: 2,2,4,6,7-Pentamethyldihydrobenzofuran-5-sulfonyl
HSA: human serum albumin
SAR: structure activity-relationship
SPPS: solid phase peptide synthesis
*t*-Bu: *tert*-butyl
TFA: trifluoroacetic acid
TIPS: triisopropylsilane
Trt-: trityl
UPLC: ultra-performance liquid chromatography
