# Supplementary material for: Design, synthesis and structure-activity relationship study of wollamide B; a new potential anti TB agent
Source: PLoS One. 2017 Apr 19;12(4):e0176088. doi: 10.1371/journal.pone.0176088 (PMC5397059; doi:10.1371/journal.pone.0176088)
Supplement: S1 File — (PDF) [file pone.0176088.s001.pdf]

Supplementary file

Design, synthesis and structure-activity relationship study of wollamide B; a new potential anti TB agent

Henok Asfaw[1], Katja Laqua[1], Anna Maria Walkowska[1], Fraser Cunningham[2,#a], Maria Santos Martinez-Martinez[2], Juan Carlos Cuevas-Zurita[2], Lluís Ballell-Pages[2], Peter Imming[1, *]

This section provides detailed information on the synthetic protocols and spectroscopic data of the target compounds. It also includes additional tables mentioned in the main text.

Table of contents

General procedure for solid phase peptide synthesis (SPPS) 2
General procedure for macrocyclization of the linear peptides 3
General procedure for deprotection of the reactive side chains protecting groups 4
Synthesis protocols for individual compounds 5
Supplementary tables 30
^{1}H NMR spectra and UPLC data for selected wollamides 32

General procedure for solid phase peptide synthesis (SPPS)

General procedure for loading of 2-chlorotrityl chloride resin

The resin (1.0 g, 1.6 mmol/g loading capacity) was weighed, transferred to a syringe fitted with a filtering frit and was allowed to swell in dry DCM for 20 min while shaking on a mechanical shaker. The amino acid to be loaded (1.2 eq.) was separately dissolved in a vial with anhydrous DCM and well mixed with DIPEA (2.5 eq.). The solution was then transferred to the syringe and the loading was allowed to be taken place for 2 h. Following that, 1 mL distilled methanol was added to the contents of the syringe and continued to be shaken for additional 15 minutes to ensure the capping of remaining trityl chloride groups. The solvent and excess reactants were then filtered off from the syringe and the resin was thoroughly washed with DCM (5X), DCM/MeOH (1:1) (4X) and MeOH (2X). Finally, the resin was dried under vacuum pressure. Mass of the dried loaded resin was weighed and the loading efficiency was calculated from the increase in the mass of the loaded resin as follows

$$\mathit{l}\mathrm{oading(mmolg^{-1})} = \ (\mathrm{M_{total}} - \mathrm{M_{resin}}\ ) \times 10^3)/(\mathrm{MW} - 36.46) \times \mathrm{M_{total}}$$

Where M_{total} = mass of the loaded resin, M_{resin} = mass of the unloaded resin, MW = molecular weight of the immobilized amino acid

Generally, the loading efficiency was calculated to be in the range of 70-95%. A weighed amount of loaded resin mass that corresponds to 0.3 mmol of the loaded Fmoc-Xaa-OH was then used for the synthesis of each linear peptide.

General procedure for deblocking of Fmoc-protecting group

Removal of the Fmoc protecting group from the resin bound Fmoc peptide prior to coupling of the next amino acid was always achieved by treating the resin with a solution of 20% piperidine in DMF (v/v) for 10 minutes and this was repeated for the second time. The resin was washed with DMF (1X) between each deprotection step and after the final deprotection, the washing was done five times with DMF to make sure the complete removal of traces of piperidine.

General procedure for HATU mediated coupling of each amino acid

In 10 mL screw fitted vial, the Fmoc-Xaa-OH (3 eq.) and the coupling reagent HATU (3 eq.) were mixed and dissolved in NMP. DIPEA (6 eq.) was then added to the solution, well mixed

by vortexing and the contents were transferred to a prewashed resin in a syringe. The peptide coupling was then allowed to taken place for 1h. Finally, the excess unreacted amino acid and other reagents were filtered-off from the syringe and resin was washed with DMF (4X) before proceeding to the next Fmoc cleavage.

General procedure for coupling to N-methylated peptide

A solution of Fmoc-Xaa-OH (3 eq.), HATU (3 eq.) and DIPEA (6 eq.) in NMP was added to the resin-bound N-methylamine peptide in the syringe and shaken for 2 h at room temperature. The excess unreacted amino acid and other reagents were filtered-off from the syringe and resin was washed with DMF (4X). The coupling was repeated for the second time.

General procedure for cleavage of the peptide from 2-chlorotrityl chloride resin

After the final Fmoc deprotection, the resin was washed with DMF (4X), dry DCM (2X) and allowed to swell in dry DCM for 20 min. A cleaving cocktail made of 20% HFIP in DCM (v/v) was then introduced into the syringe and the cleavage of the full-length hexapeptide was allowed to taken place for 1h. Following this, the filtrate was collected in already weighed round bottom flak and the drained resin was further washed twice with DCM and all the washings were collected in the flask. Finally, the solvent was removed under vacuum and kept on the oil pump for prolonged period to exclude all the residual HFIP. The obtained solid mass was directly used for the next step without further purification.

General procedure for macrocyclization of the linear peptides

The linear hexapeptide prepared by SPPS approach was dissolved in DMF (1mM) and cooled to 0°C in an ice bath. To the solution was added HATU (3 equiv), and HOAt (3 equiv) under vigorous stirring. DIPEA (10 equiv) was then added to the dilute solution and the reaction mixture was allowed to warm slowly to the room temperature and kept stirring for 3 days. Following these, the solvent was removed under reduced pressure and the residue was dissolved in 40 mL saturated $NaHCO_3$ while stirring for about 10 minute. 20 mL of ethyl acetate was added to the mixture and stirred for further 5 minutes. The content was then transferred to a separtory funnel and the organic layer was collected. The aqueous phase was washed twice with 20 mL ethyl acetate. The combined ethyl acetate fraction was washed with brine, dried by anhydrous $MgSO_4$, filtered and concentrated under a reduced pressure. The

obtained crude solid mass was purified by a column chromatography to give a pure cyclic hexapeptides whose reactive amino acid side chains were protected.

General procedure for deprotection of the reactive side chains protecting groups

The protecting groups from reactive amino acid side chains of the synthesized cyclic hexapeptides were removed with a cleaving cocktail made up of TFA/TIPS/H2O (95:2.5/2.5/) for amino acids that contain Trt/Pbf protecting groups and with TFA/DCM (1:1) for amino acids containing Boc/tBu protecting group. The cleaving solution was then removed under a strong pressure and the residue was purified by column chromatography.

Synthesis protocols for individual compounds

Synthesis of H_2N-D-Leu-Val-Asn(Trt)-D-Orn(Boc)-Trp(Boc)-Leu-COOH (1a)

SPPS approach was used to prepare **1a** according to the general procedure described under section S1. The synthesis was initiated from a resin loaded with Fmoc-Leu-OH (0.43 g, 0.3 mmol). Crude % yield (87%), ESI-MS m/z calcd for $C_{66}H_{89}N_9O_{12}$:1199.66; found: 1200.47 $[M+H]^+$

Synthesis of cyclo (Trp (Boc)-D-Orn(Boc)-Asn(Trt)-Val-D-Leu-Leu) (1b)

Compound **1b** was obtained through a macrocyclization of **1a** (0.2 g, 0.17 mmol) with HATU (0.19g, 0.51 mmol), HOBt (0.07 g, 0.51 mmol) and DIPEA (0.19 g, 1.7 mmol) according to the procedure described in section S2. The crude was purified by a column chromatography using a silica gel as a stationary phase and chloroform/methanol (49:1) as a mobile phase to give a white amorphous solid mass. Yield (68%), ESI-MS m/z calcd $C_{66}H_{87}N_9O_{11}$:1181.65; found: 1180.90 $[M-H]^-$. R_f = 0.23 in $CHCl_3$/MeOH (95:5).

Synthesis of cyclo (Trp-D-Orn-Asn-Val-D-Leu-Leu) (1c)

Compound **1b** (0.05 g, 0.05 mmol) was treated with 2 mL of TFA/TIPS/H_2O (95:2.5/2.5/) for 3 h. The cleaving solution was removed under a strong pressure and the residue was purified by column chromatography using a silica gel as a stationary phase and chloroform/methanol/ammonia solution (80:20:1) as a mobile phase to give a white amorphous solid mass. Yield (91%), mp 234-236 °C, UPLC-MS (UV) purity: 99.9%, RT 0.75 min, ESI-MS m/z calcd for $C_{37}H_{57}N_9O_7$: 739.44; found: 740.44 $[M+H]^+$.

^{1}H NMR (400 MHz, DMSO-d6) δ 10.81 (d, *J* = 2.4 Hz, 1H), 8.33 (t, *J* = 6.5 Hz, 2H), 8.23 (d, *J* = 8.0 Hz, 1H), 7.53 (ddt, *J* = 23.6, 15.8, 7.4 Hz, 6H), 7.34 (dd, *J* = 17.5, 8.1 Hz, 2H), 7.12 (d, *J* = 2.4 Hz, 1H), 7.05 (ddd, *J* = 8.1, 6.9, 1.2 Hz, 1H), 6.97 (ddd, *J* = 7.9, 5.3, 1.1 Hz, 2H), 4.59 (dt, *J* = 8.3, 6.1 Hz, 1H), 4.42 (q, *J* = 7.2 Hz, 1H), 4.29 (ddt, *J* = 10.0, 7.8, 3.4 Hz, 2H), 4.17 (q, *J* = 7.5 Hz, 1H), 4.00 (dd, *J* = 7.5, 4.4 Hz, 1H), 3.21 – 3.13 (m, 2H), 3.00 – 2.92 (m, 1H), 2.69 – 2.58 (m, 3H), 2.25 (td, *J* = 7.1, 4.6 Hz, 1H), 1.60 – 1.21 (m, 10H), 0.97 – 0.68 (m, 18H). ^{13}C NMR (126 MHz, CD_3OD-d_4) δ 175.09, 173.36, 172.61, 172.52, 172.33, 171.98, 171.97, 136.69, 127.07, 123.34, 121.11, 118.55, 117.96, 110.86, 109.19, 60.31, 55.93, 52.01, 51.89, 51.17, 50.01, 40.69, 39.48, 39.13, 35.67, 29.13, 27.17, 26.22, 24.67, 24.50, 24.42, 21.59, 21.57, 21.30, 21.07, 18.07, 16.54.

Synthesis of H_2N-D-Leu-Val-Asn(Trt)-D-Orn(Boc)-Trp(Boc)-Ala-COOH (2a)

SPPS approach was used to prepare **2a** according to the general procedure described under section S1. The synthesis was initiated from a resin loaded with Fmoc-Leu-OH (0.28 g, 0.3 mmol). Crude % yield (95%), ESI-MS m/z calcd for $C_{63}H_{83}N_9O_{12}$: 1157.62; found: 1158.54 $[M+Na]^+$.

Synthesis of cyclo(Trp (Boc)-D-Orn(Boc)-Asn(Trt)-Val-D-Leu-Ala) (2b)

Compound 2b was obtained through a macrocyclization of **2a** (0.34 g, 0.3 mmol) with HATU (0.34 g, 0.9 mmol), HOBt (0.12 g, 0.9 mmol) and DIPEA (0.52 mL, 3.0 mmol) according to the procedure described in section S2. It was purified by a column chromatography using a silica gel as a stationary phase and chloroform/methanol (19:1) as a mobile phase to give a white amorphous solid mass. Yield (45%), ESI-MS m/z calcd for $C_{63}H_{81}N_9O_{11}$: 1139.61; found: 1162.59 $[M+Na]^+$. R_f = 0.35 in $CHCl_3$/MeOH (95:5),

Synthesis of cyclo(Trp-D-Orn-Asn-Val-D-Leu-Ala) (2c)

Compound 2b (0.08 g, 0.07 mmol) was treated with 1.5 mL of TFA/TIPS/H_2O (95:2.5/2.5/) for 3 h. The cleaving solution was removed under a strong pressure and the residue was purified by column chromatography using a silica gel as a stationary phase and chloroform/methanol/ammonia solution (80:20:1) as a mobile phase to give a white amorphous solid mass. Yield (80%), mp 230-232 °C, UPLC-MS (UV) purity: 98%, RT 0.64 min, ESI-MS m/z calcd for $C_{34}H_{51}N_9O_7$: 697.39; found: 698.39 $[M+H]^+$. ^1H NMR (500 MHz, CD_3OD-d_4) δ 7.56 (d, J = 7.9 Hz, 1H), 7.32 (d, J = 8.1 Hz, 1H), 7.14 (d, J = 2.4 Hz, 1H), 7.09 (t, J = 7.4 Hz, 1H), 7.01 (t, J = 7.6 Hz, 1H), 4.66 (t, J = 4.6 Hz, 1H), 4.58 – 4.44 (m, 3H), 4.38 (dd, J = 10.2, 4.9 Hz, 1H), 3.99 (dd, J = 4.9, 2.2 Hz, 1H), 3.25 (dd, J= 15.0, 4.5 Hz, 1H), 3.17 – 3.07 (m, 2H), 2.82 (dd, J = 17.0, 9.4 Hz, 2H), 2.71 – 2.63 (m, 1H), 2.33 – 2.21 (m, 1H), 1.83 (dt, J = 12.2, 7.0 Hz, 1H), 1.78 – 1.71 (m, 1H), 1.63 – 1.46 (m, 5H), 1.26 – 1.18 (m, 3H), 1.05 – 0.87 (m, 12H). ^{13}C NMR (126 MHz, CD_3OD-d_4) δ 175.13, 173.52, 172.72, 172.65, 172.21, 172.14, 172.01, 136.71, 127.06, 123.50, 121.15, 118.56, 117.95, 110.88, 108.93, 65.45, 60.68, 56.01, 51.70, 51.47, 49.85, 39.94, 38.80, 35.46, 29.07, 27.13, 25.79, 24.48, 23.30, 21.51, 21.27, 18.02, 16.78, 16.35.

Synthesis of H_2N-D-Leu-Val-Asn(Trt)-D-Orn(Boc)-Ala-Leu-COOH (3a)

SPPS approach was used to prepare **3a** according to the general procedure described under section S1. The synthesis was initiated from a resin loaded with Fmoc-Leu-OH (0.28 g, 0.3 mmol). Crude % yield (103%), ESI-MS m/z calcd for $C_{53}H_{76}N_8O_{10}$: 984.57; found: 985.52 $[M+H]^+$.

Synthesis of cyclo(Ala-D-Orn (Boc)-Asn(Trt)-Val-D-Leu-Leu) (3b)

Compound **3b** was obtained through a macrocyclization of **3a** (0.36g, 0.37 mmol) with HATU (0.42g, 1.11 mmol), HOBt (0.15 g, 1.11 mmol) and DIPEA (0.65 mL, 3.7 mmol) according to the procedure described in section S2. It was purified by a column chromatography using a silica gel as a stationary phase and chloroform/methanol (19:1) as a mobile phase to give a white amorphous solid mass. Yield (43%), ESI-MS m/z calcd for $C_{53}H_{74}N_8O_9$: 966.56; found: 989.57 $[M+Na]^+$. R_f = 0.08 in $CHCl_3$/MeOH (95:5).

Synthesis of cyclo(Ala-D-Orn-Asn-Val-D-Leu-Leu) (3c)

Compound **3b** (0.05 g, 0.05 mmol) was treated with 1.0 mL of TFA/TIPS/H_2O (95:2.5/2.5/) for 3 h. The cleaving solution was removed under a strong pressure and the residue was purified by column chromatography using a silica gel as a stationary phase and chloroform/methanol/ammonia solution (80:20:1) as a mobile phase to give a white amorphous solid mass. Yield (92%), mp 265-267 °C, UPLC-MS (UV) purity: 90%, RT 0.64 min, ESI-MS m/z calcd for $C_{29}H_{52}N_8O_7$: 624.40; found: 625.40 $[M+H]^+$. ^{1}H NMR (500 MHz, DMSO-d_6) δ 8.43 (d, J = 6.6 Hz, 1H), 8.29 – 8.16 (m, 2H), 7.93 (s, 1H), 7.74 (d, J = 7.0 Hz, 1H), 7.63 (s, 3H), 7.45 (d, J = 6.9 Hz, 2H), 7.24 (d, J = 8.2 Hz, 1H), 6.86 (s, 1H), 4.58 (d, J = 6.9 Hz, 2H), 4.39 (d, J = 7.7 Hz, 1H), 4.28 (d, J = 7.1 Hz, 1H), 4.20 (d, J = 7.5 Hz, 1H), 4.01 (dd, J = 7.3, 3.9 Hz, 2H), 2.76 (d, J = 8.5 Hz, 1H), 2.58 (d, J = 6.3 Hz, 2H), 2.23 (d, J = 9.4 Hz, 1H), 1.76 – 1.32 (m, 10H), 1.24 (d, J = 7.2 Hz, 3H), 0.86 (dt, J = 27.1, 6.2 Hz, 18H).

Synthesis of H_2N-D-Leu-Val-Ala-D-Orn(Boc)-Trp(Boc)-Leu-COOH (4a)

SPPS approach was used to prepare **4a** according to the general procedure described under section S1. The synthesis was initiated from a resin loaded with Fmoc-Leu-OH (0.28 g, 0.3 mmol). Crude % yield (99%), ESI-MS m/z calcd for $C_{46}H_{74}N_8O_{11}$: 914.55; found: 915.50 $[M+H]^+$.

Synthesis of cyclo(Trp(Boc)-D-Orn(Boc)-Ala-Val-D-Leu-Leu) (4b)

Compound **4b** was obtained through a macrocyclization of **4a** (0.27 g, 0.3 mmol) with HATU (0.34 g, 0.9 mmol), HOBt (0.12 g, 0.9 mmol) and DIPEA (0.52 mL, 3.0 mmol) according to the procedure described in section S2. It was purified by a column chromatography using a silica gel as a stationary phase and chloroform/methanol (19:1) as a mobile phase to give a white amorphous solid mass. Yield (56%), ESI-MS m/z calcd for $C_{46}H_{72}N_8O_{10}$: 896.54; found: 919.47 $[M+Na]^+$. R_f = 0.02 in $CHCl_3$/MeOH (96:4),

Synthesis of cyclo(Trp-D-Orn-Ala-Val-D-Leu-Leu) (4c)

Compound **4b** (0.05 g, 0.06 mmol) was treated with 1.0 mL of TFA/TIPS/H_2O (95:2.5/2.5/) for 1 h. The cleaving solution was removed under a strong pressure and the residue was purified by column chromatography using a silica gel as a stationary phase and chloroform/methanol/ammonia solution (80:20:1) as a mobile phase to give a white amorphous solid mass. Yield (80%), mp 234-237 °C, UPLC-MS (UV) purity: 99.9%, RT 0.81 min, ESI-MS m/z calcd for $C_{36}H_{56}N_8O_6$: 696.43; found: 697.43$[M+H]^+$. ^{1}H NMR (500 MHz, CD_3OD-d_4) δ 7.56 (d, J = 7.9 Hz, 1H), 7.33 (d, J = 8.2 Hz, 1H), 7.19 (s, 1H), 7.09 (t, J = 7.7 Hz, 1H), 7.00 (t, J = 7.5 Hz, 1H), 4.51 (dd, J = 8.3, 4.4 Hz, 1H), 4.40 (q, J = 7.4 Hz, 2H), 4.29 (dt, J = 10.0, 4.7 Hz, 2H), 4.04 (d, J = 4.6 Hz, 1H), 3.18 (dd, J = 15.0, 8.3 Hz, 1H), 2.75 (dtt, J = 20.0, 13.1, 7.1 Hz, 3H), 2.30 (h, J = 6.5 Hz, 1H), 1.56 (dddd, J = 52.5, 31.1, 15.0, 7.7 Hz, 7H), 1.33 (dd, J = 24.0, 11.0 Hz, 6H), 1.01 – 0.74 (m, 18H). ^{13}C NMR (126 MHz, CD_3OD-d_4) δ 174.71, 173.35, 172.44, 172.15, 172.11, 171.95, 136.71, 127.17, 123.39, 121.28, 118.66, 118.12, 110.93, 108.95, 59.72, 55.10, 52.45, 52.18, 51.40, 48.96, 40.93, 39.29, 38.80, 29.00, 26.24, 26.13, 24.61, 24.41, 23.07, 21.56, 21.51, 21.33, 21.14, 18.22, 16.33, 16.29.

Synthesis of H_2N-D-Leu-Ala-Asn(Trt)-D-Orn(Boc)-Trp(Boc)-Leu-COOH (5a)

SPPS approach was used to prepare **5a** according to the general procedure described under section S1. The synthesis was initiated from a resin loaded with Fmoc-Leu-OH (0.28 g, 0.3 mmol). Crude % yield (87%), ESI-MS m/z calcd for $C_{64}H_{85}N_9O_{12}$: 1171.63; found: 1194.48 $[M+Na]^+$.

Synthesis of cyclo(Trp(Boc)-D-Orn(Boc)-Asn(Trt)-Ala-D-Leu-Leu) (5b)

Compound **5b** was obtained through a macrocyclization of **5a** (0.3 g, 0.26 mmol) with HATU (0.3 g, 0.78 mmol), HOBt (0.11g, 0.78 mmol) and DIPEA (0.45 mL, 2.6 mmol) according to the procedure described in section S2. It was purified by a column chromatography using a silica gel as a stationary phase and chloroform/methanol (19:1) as a mobile phase to give a white amorphous solid mass. Yield (56%), ESI-MS m/z calcd for $C_{64}H_{83}N_9O_{11}$: 1153.62; found: 1176.57 $[M+Na]^+$. R_f = 0.29 in $CHCl_3$/MeOH (95:5).

Synthesis of cyclo(Trp-D-Orn-Asn-Ala-D-Leu-Leu) (5c)

Compound **5b** (0.14 g, 0.12 mmol) was treated with 2.5 mL of TFA/TIPS/H_2O (95:2.5/2.5/) for 3 h. The cleaving solution was removed under a strong pressure and the residue was purified by column chromatography using a silica gel as a stationary phase and chloroform/methanol/ammonia solution (80:20:1) as a mobile phase to give a white amorphous solid mass. Yield (88%), mp 231-234 °C, UPLC-MS (UV) purity: 99.9%, RT 0.71 min, ESI-MS m/z calcd for $C_{35}H_{53}N_9O_7$: 711.41; found: 712.41$[M+H]^+$. ^{1}H NMR (400 MHz, CD_3OD-d_4) δ 7.57 (dq, J = 7.8, 1.1 Hz, 1H), 7.32 (dt, J = 8.1, 1.0 Hz, 1H), 7.13 (d, J = 6.0 Hz, 1H), 7.08 (ddd, J = 8.2, 7.0, 1.3 Hz, 1H), 7.01 (dddd, J = 8.0, 7.1, 2.4, 1.1 Hz, 1H), 4.70 – 4.61 (m, 1H), 4.57 (ddd, J = 9.0, 5.0, 3.8 Hz, 1H), 4.54 – 4.45 (m, 1H), 4.40 – 4.20 (m, 2H), 4.10 (q, J = 7.5 Hz, 1H), 3.29 – 3.20 (m, 1H), 3.18 – 2.96 (m, 3H), 2.81 – 2.64 (m, 2H), 1.88 – 1.79 (m, 1H), 1.76 – 1.25 (m, 12H), 1.03 – 0.79 (m, 12H). ^{13}C NMR (126 MHz, CD_3OD-d_4) δ 174.22, 173.60, 173.51, 172.65, 172.37, 172.25, 171.91, 136.69, 127.07, 123.40, 121.11, 118.55, 117.95, 110.88, 109.22, 55.74, 52.48, 51.97, 51.18, 50.66, 49.74, 40.50, 39.99, 39.52, 35.72, 27.21, 26.92, 26.53, 24.67, 24.52, 21.78, 21.63, 21.30, 20.85, 15.74.

Synthesis of H_2N-D-Ala-Val-Asn(Trt)-D-Orn(Boc)-Trp(Boc)-Leu-COOH (6a)

SPPS approach was used to prepare **6a** according to the general procedure described under section S1. The synthesis was initiated from a resin loaded with Fmoc-Leu-OH (0.43 g, 0.3 mmol). Crude % yield (72%), ESI-MS m/z calcd for $C_{63}H_{83}N_9O_{12}$: 1157.62; found: 1180.24 $[M+Na]^+$.

Synthesis of cyclo(Trp(Boc)-D-Orn(Boc)-Asn(Trt)-Val-D-Ala-Leu) (6b)

Compound **6b** was obtained through a macrocyclization of **6a** (0.23 g, 0.2 mmol) with HATU (0.23 g, 0.6 mmol), HOBt (0.08 g, 0.6 mmol) and DIPEA (0.35 mL, 2.0 mmol) according to the procedure described in section S2. It was purified by a column chromatography using a silica gel as a stationary phase and chloroform/methanol (97:3) as a mobile phase to give a white amorphous solid mass. Yield (64 %), ESI-MS m/z calcd for $C_{63}H_{81}N_9O_{11}$: 1139.61; found: 1162.30 $[M+Na]^+$. R_f = 0.09 in $CHCl_3$/MeOH (97:3).

Synthesis of cyclo(Trp-D-Orn-Asn-Val-D-Ala-Leu) (6c)

Compound **6c** (0.15 g, 0.13 mmol) was treated with 3.5 mL of TFA/TIPS/H_2O (95:2.5/2.5/) for 3 h. The cleaving solution was removed under a strong pressure and the residue was purified by column chromatography using a silica gel as a stationary phase and chloroform/methanol/ammonia solution (80:20:1) as a mobile phase to give a white amorphous solid mass. Yield (72%), mp 230-232 °C, UPLC-MS (UV) purity: 92%, RT 0.65 min, ESI-MS m/z calcd for $C_{34}H_{51}N_9O_7$: 697.39; found: 698.39$[M+H]^+$. ^{1}H NMR (400 MHz, CD_3OD-d_4) δ 7.57 (dt, J = 7.8, 1.0 Hz, 1H), 7.32 (dt, J = 8.1, 0.9 Hz, 1H), 7.13 (s, 1H), 7.08 (ddd, J = 8.1, 7.0, 1.2 Hz, 1H), 7.01 (ddd, J = 8.0, 7.0, 1.1 Hz, 1H), 4.69 (dd, J = 6.4, 4.6 Hz, 1H), 4.58 (dd, J = 9.1, 5.3 Hz, 1H), 4.47 – 4.34 (m, 3H), 4.03 – 3.95 (m, 1H), 3.29 – 3.22 (m, 1H), 3.12 (dd, J = 14.7, 9.1 Hz, 1H), 3.05 – 2.98 (m, 1H), 2.83 – 2.67 (m, 3H), 2.28 (pd, J = 7.0, 4.7 Hz, 1H), 1.83 (dddd, J = 14.2, 9.6, 6.8, 5.0 Hz, 1H), 1.68 (ddt, J = 14.2, 9.8, 4.8 Hz, 1H), 1.63 – 1.29 (m, 8H), 1.06 – 0.83 (m, 12H). ^{13}C NMR (101 MHz, CD_3OD-d_4) δ 175.64, 173.31, 172.60, 172.38, 172.32, 172.09, 171.74, 136.69, 127.07, 123.35, 121.11, 118.55, 117.95, 110.87, 109.14, 60.56, 55.92, 51.68, 51.19, 50.21, 49.07, 40.61, 38.82, 35.51, 29.22, 27.14, 26.22, 24.58, 23.49, 21.72, 21.19, 17.90, 16.61, 15.68.

Synthesis of H_2N-D-Leu-Ile-Asn(Trt)-D-Orn(Boc)-Trp(Boc)-Leu-COOH (7a)

SPPS approach was used to prepare **7a** according to the general procedure described under section S11**.** The synthesis was initiated from a resin loaded with Fmoc- Leu-OH (0.43 g, 0.3 mmol). Crude % yield (88%), ESI-MS m/z calcd for $C_{66}H_{89}N_9O_{12}$:1213.68; found: 1212.54 $[M-H]^-$.

Synthesis of cyclo (D-Leu-Ile-Asn(Trt)-D-Orn(Boc)-Trp(Boc)-Leu) (7b)

Compound **7b** was obtained through a macrocyclization of **7a** (0.32 g, 0.26 mmol) with HATU (0.3 g, 0.78 mmol), HOBt (0.11 g, 0.78 mmol) and DIPEA (0.45 mL, 2.6 mmol) according to the procedure described in section S2. The crude was purified by a column chromatography using a silica gel as a stationary phase and chloroform/methanol (49:1) as a mobile phase to give a white amorphous solid mass. Yield (52%), ESI-MS m/z calcd for $C_{66}H_{87}N_9O_{11}$:1195.67; found: 1194.49$[M-H]^-$. R_f = 0.11 in $CHCl_3$/MeOH (49:1).

Synthesis of cyclo(D-Leu-Ile-Asn-D-Orn-Trp-Leu) (7c)

Compound **7b** (0.12 g, 0.1 mmol) was treated with 2.5 mL of TFA/TIPS/H_2O (95:2.5/2.5/) for 3 h. The cleaving solution was removed under a strong pressure and the residue was purified by column chromatography using a silica gel as a stationary phase and chloroform/methanol/ammonia solution (70:30:1) as a mobile phase to give a white amorphous solid mass. Yield (65%), mp 232-235 °C, UPLC-MS (UV) purity: 97%, RT 0.8 min, ESI-MS m/z calcd for $C_{38}H_{59}N_9O_7$: 753.45; found: 754.45 $[M+H]^+$. ^{1}H NMR (400 MHz, CD_3OD-d_4) δ 7.57 (dt, J = 7.8, 1.0 Hz, 1H), 7.32 (dt, J = 8.1, 0.9 Hz, 1H), 7.13 (s, 1H), 7.08 (ddd, J = 8.1, 7.0, 1.2 Hz, 1H), 7.01 (ddd, J = 8.0, 7.1, 1.1 Hz, 1H), 4.67 (dd, J = 6.0, 4.3 Hz, 1H), 4.56 (dd, J = 9.3, 5.1 Hz, 1H), 4.51 – 4.40 (m, 2H), 4.37 (dd, J = 10.0, 4.9 Hz, 1H), 4.07 (d, J = 4.5 Hz, 1H), 3.28 – 3.21 (m, 1H), 3.16 – 3.05 (m, 2H), 2.86 – 2.68 (m, 3H), 2.02 (ddt, J = 9.3, 6.9, 4.6 Hz, 1H), 1.87 – 1.80 (m, 1H), 1.72 – 1.44 (m, 8H), 1.36 – 1.28 (m, 3H), 1.03 – 0.83 (m, 18H). ^{13}C NMR (101 MHz, CD_3OD-d_4) δ 175.26, 173.35, 172.65, 172.49, 172.34, 172.13, 171.78, 136.70, 127.06, 123.34, 121.10, 118.56, 117.95, 110.85, 109.14, 59.72, 56.07, 52.01, 51.15, 50.04, 40.69, 39.52, 38.79, 35.97, 35.51, 27.23, 26.00, 24.69, 24.60, 24.52, 23.49, 22.32, 21.59, 21.57, 21.33, 21.03, 14.71, 10.76.

Synthesis of H_2N-D-Leu-Met-Asn(Trt)-D-Orn(Boc)-Trp(Boc)-Leu-COOH (8a)

SPPS approach was used to prepare **8a** according to the general procedure described under section S1**.** The synthesis was initiated from a resin loaded with Fmoc- Leu-OH (0.31 g, 0.3 mmol). Crude % yield (96%), ESI-MS m/z calcd for $C_{66}H_{89}N_9O_{12}S$: 1231.64; found: 1230.64 $[M-H]^+$.

Synthesis of cyclo (D-Leu-Met-Asn(Trt)-D-Orn(Boc)-Trp(Boc)-Leu) (8b)

Compound **8b** was obtained through a macrocyclization of **8a** (0.36 g, 0.29 mmol) with HATU (0.33 g, 0.87 mmol), HOBt (0.12 g, 0.87 mmol) and DIPEA (0.51 mL, 2.9 mmol) according to the procedure described in section S2. The crude was purified by a column chromatography using a silica gel as a stationary phase and chloroform/methanol (49:1) as a mobile phase to give a white amorphous solid mass. Yield (40%), ESI-MS m/z calcd for $C_{66}H_{87}N_9O_{11}S$:1213.62; found: 1236.63 $[M+Na]^+$. R_f = 0.1 in $CHCl_3$/MeOH (49:1)

Synthesis of cyclo (D-Leu-Met-Asn-D-Orn-Trp-Leu) (8c)

Compound **8b** (0.13 g, 0.11 mmol) was treated with 3 mL of TFA/TIPS/H_2O (95:2.5/2.5/) for 3 h. The cleaving solution was removed under a strong pressure and the residue was purified by column chromatography using a silica gel as a stationary phase and chloroform/methanol/ammonia solution (70:30:1) as a mobile phase to give a white amorphous solid mass. Yield (74%), mp 233-235 °C, UPLC-MS (UV) purity: 95%, RT 0.75 min, ESI-MS m/z calcd for $C_{37}H_{57}N_9O_7S$: 771.41; found: 772.41 $[M+H]^+$. ^1H NMR (400 MHz, CD_3OD-d_4) δ 7.58 (dt, *J* = 7.8, 1.0 Hz, 1H), 7.32 (dt, *J* = 8.1, 0.9 Hz, 1H), 7.12 (s, 1H), 7.08 (ddd, *J* = 8.2, 7.0, 1.2 Hz, 1H), 7.01 (ddd, *J* = 8.0, 7.0, 1.1 Hz, 1H), 4.69 (dd, *J* = 6.0, 4.5 Hz, 1H), 4.59 (dd, *J* = 9.3, 5.1 Hz, 1H), 4.49 (t, *J* = 7.1 Hz, 1H), 4.39 – 4.25 (m, 3H), 3.25 (ddd, *J* = 14.7, 5.2, 0.8 Hz, 1H), 3.16 – 3.04 (m, 2H), 2.85 – 2.71 (m, 3H), 2.65 (ddd, *J* = 13.0, 7.8, 4.9 Hz, 1H), 2.53 (ddd, *J* = 13.4, 8.3, 7.4 Hz, 1H), 2.19 (dtd, *J* = 14.3, 8.1, 4.3 Hz, 1H), 2.09 (s, 3H), 1.98 (dddd, *J* = 14.5, 9.8, 7.4, 4.9 Hz, 1H), 1.87 – 1.79 (m, 1H), 1.73 – 1.39 (m, 8H), 1.36 – 1.25 (m, 1H), 1.02 – 0.80 (m, 12H). ^{13}C NMR (126 MHz, CD_3OD-d_4) δ 175.03, 173.32, 173.12, 172.57, 172.51, 172.15, 171.56, 136.68, 127.09, 123.31, 121.08, 118.54, 117.97, 110.84, 109.23, 56.02, 53.54, 52.48, 51.55, 51.10, 50.10, 40.93, 39.48, 38.74, 35.42, 29.79, 29.68, 27.35, 25.94, 24.69, 24.52, 23.34, 21.53, 21.43, 21.16, 17.28, 15.85, 13.64.

Synthesis of H_2N-D-Leu-Val-D-Orn(Boc)-D-Orn(Boc)-Trp(Boc)-Leu-COOH (9a)

SPPS approach was used to prepare **9a** according to the general procedure described under section S1. The synthesis was initiated from a resin loaded with Fmoc- Leu-OH (0.3 g, 0.3 mmol). Crude % yield (95 %), ESI-MS m/z calcd for $C_{53}H_{87}N_9O_{13}$:1057.64: found: 1056.56 $[M-H]^-$.

Synthesis of cyclo(D-Leu-Val-D-Orn(Boc)-D-Orn(Boc)-Trp(Boc)-Leu) (9b)

Compound **9b** was obtained through a macrocyclization of **9a** (0.3 g, 0.29 mmol) with HATU (0.3 g, 0.86 mmol), HOBt (0.12 g, 0.86 mmol) and DIPEA (0.5 mL, 2.87 mmol) according to the procedure described in section S2. The crude was purified by a column chromatography using a silica gel as a stationary phase and chloroform/methanol (49:1) as a mobile phase to give a white amorphous solid mass. Yield (40%), ESI-MS m/z calcd for $C_{53}H_{85}N_9O_{12}$: 1039.63; found: 1038.51 $[M-H]^-$.R_f = 0.12 in $CHCl_3$/MeOH (49:1)

Synthesis of cyclo (D-Leu-Val-D-Orn-D-Orn-Trp-Leu) (9c)

Compound **9b** (0.11 g, 0.11 mmol) was treated with 2.5 mL of TFA/DCM (95:2.5/2.5/) for 1 h. The cleaving solution was removed under a strong pressure and the product was washed with cold ether and kept under strong vacuum to give a white amorphous solid mass. Yield (70%), mp 235-237 °C, UPLC-MS (UV) purity: 97%, RT 0.68 min, ESI-MS m/z calcd for $C_{38}H_{61}N_9O_6$: 739.47; found: 740.47 $[M+H]^+$. ^{1}H NMR (400 MHz, CD_3OD-d_4) δ 7.61 (dt, *J* = 7.9, 1.0 Hz, 1H), 7.36 (dt, *J* = 8.1, 0.9 Hz, 1H), 7.22 (s, 1H), 7.12 (ddd, *J* = 8.2, 7.0, 1.2 Hz, 1H), 7.04 (ddd, *J* = 8.0, 7.1, 1.1 Hz, 1H), 4.55 – 4.37 (m, 4H), 4.23 (dd, *J* = 8.9, 3.9 Hz, 1H), 3.94 – 3.84 (m, 1H), 3.41 – 3.34 (m, 1H), 3.12 (dd, *J* = 14.8, 9.5 Hz, 1H), 2.93 (t, *J* = 7.3 Hz, 2H), 2.77 – 2.58 (m, 2H), 2.05 (ddd, *J* = 13.1, 9.1, 6.4 Hz, 2H), 1.79 – 1.48 (m, 9H), 1.45 – 1.24 (m, 4H), 1.08 – 0.73 (m, 18H). ^{13}C NMR (101 MHz, CD_3OD-d_4) δ 173.55, 173.07, 172.70, 172.52, 172.47, 171.88, 127.00, 123.46, 121.28, 118.68, 117.86, 111.03, 109.07, 60.74, 56.07, 53.40, 52.43, 51.48, 51.05, 50.31, 39.85, 39.55, 38.73, 29.03, 28.27, 27.94, 27.00, 24.46, 24.31, 23.75, 22.93, 22.08, 21.67, 21.19, 20.25, 18.30, 17.89.

Synthesis of H_2N-Trp(Boc)-Leu-D-Leu-Ile-Ser(tBu)-D-Orn(Boc)-COOH (10a)

SPPS approach was used to prepare **10a** according to the general procedure described under section S1. The synthesis was initiated from a resin loaded with Fmoc- D-Orn(Boc)-OH (0.33 g, 0.3 mmol). Crude % yield (78%), ESI-MS m/z calcd for $C_{51}H_{84}N_8O_{12}$: 1000.62; found: 1023.74 $[M+Na]^+$.

Synthesis of cyclo(Trp(Boc)-Leu-D-Leu-Ile-Ser(tBu)-D-Orn(Boc)) (10b)

Compound **10b** was obtained through a macrocyclization of **10a** (0.23 g, 0.23 mmol) with HATU (0.27 g, 0.67 mmol), HOBt (0.09 g, 0.67 mmol) and DIPEA (0.41 mL, 2.33 mmol) according to the procedure described in section S2. The crude was purified by a column chromatography using a silica gel as a stationary phase and chloroform/methanol (49:1) as a mobile phase to give a white amorphous solid mass. Yield (68%), ESI-MS m/z calcd for $C_{51}H_{82}N_8O_{11}$: 982.61; found: 1005.75 $[M+Na]^+$. R_f = 0.04in $CHCl_3$/MeOH (49:1),

Synthesis of cyclo(Trp-Leu-D-Leu-Ile-Ser-D-Orn) (10c)

Compound **10b** (0.14 g, 0.14 mmol) was treated with 4 mL of TFA/DCM (1:1) for 1 h. The cleaving solution was removed under a strong pressure and the residue was purified by column chromatography using a silica gel as a stationary phase and chloroform/methanol/ammonia solution (80:20:1) as a mobile phase to give a white amorphous solid mass. Yield (45%), mp 95-97 °C, UPLC-MS (UV) purity: 98%, RT 0.8 min, ESI-MS m/z calcd for $C_{34}H_{52}N_8O_7$: 726.44; found: 727.44 $[M+H]^+$. ^1H NMR (400 MHz, CD_3OD-d_4) δ 7.57 (dt, *J* = 7.9, 1.0 Hz, 1H), 7.38 – 7.32 (m, 1H), 7.17 (s, 1H), 7.09 (ddd, *J* = 8.1, 6.9, 1.1 Hz, 1H), 7.00 (ddd, *J* = 7.9, 7.0, 1.0 Hz, 1H), 4.51 (dd, *J* = 9.3, 4.7 Hz, 1H), 4.44 (dt, *J* = 9.2, 5.2 Hz, 2H), 4.35 (dt, *J* = 14.5, 7.4 Hz, 2H), 4.11 (d, *J* = 4.7 Hz, 1H), 3.98 (dd, *J* = 11.3, 5.0 Hz, 1H), 3.83 (dd, *J* = 11.3, 4.2 Hz, 1H), 3.08 (dd, *J* = 14.8, 9.4 Hz, 1H), 2.77 (qdd, *J* = 12.6, 8.6, 6.3 Hz, 2H), 2.04 (ddt, *J* = 9.3, 6.9, 4.5 Hz, 1H), 1.72 – 1.23 (m, 12H), 1.02 – 0.75 (m, 18H). ^{13}C NMR (101 MHz, CD_3OD-d_4) δ 174.91, 172.74, 172.49, 172.41, 172.12, 171.05, 136.74, 127.02, 123.37, 121.19, 118.58, 117.88, 110.99, 109.07, 61.76, 59.53, 56.91, 55.59, 52.50, 52.06, 51.26, 48.22, 48.01, 47.79, 47.79, 47.61, 47.58, 47.37, 47.16, 46.94, 40.71, 39.43, 38.72, 35.76, 26.72, 26.23, 24.58, 24.52, 24.46, 23.08, 21.51, 21.49, 21.27, 21.18, 16.94, 14.82, 10.72.

Synthesis of cyclo(Trp-Leu-D-Leu-Ile-Ser(t-Bu)-D-Orn) (11c)

11c was isolated as a side product when **10c** was prepared due to incomplete deprotection of the t-Bu protecting group of serine. Yield (30%), mp 104-107 °C, UPLC-MS (UV) purity: 97%, RT 0.92 min, ESI-MS m/z calcd for $C_{41}H_{66}N_8O_7$: 782.51; found: 783.51 $[M-H]^-$. ^1H NMR (500 MHz, CD_3OD-d_4) δ 7.57 (dt, *J* = 7.9, 1.0 Hz, 1H), 7.33 (dt, *J* = 8.2, 0.9 Hz, 1H), 7.18 (s, 1H), 7.09 (ddd, *J* = 8.2, 7.0, 1.2 Hz, 1H), 7.01 (ddd, *J* = 7.9, 7.1, 1.0 Hz, 1H), 4.56 (dd, *J* = 9.0, 4.2 Hz, 1H), 4.44 (dd, *J* = 7.3, 6.1 Hz, 2H), 4.33 (dd, *J* = 8.0, 6.9 Hz, 1H), 4.14 (t, *J* = 7.5 Hz, 1H), 4.09 (d, *J* = 4.5 Hz, 1H), 3.63 (dd, *J* = 8.9, 6.0 Hz, 1H), 3.57 – 3.47 (m, 1H), 3.42 – 3.33 (m, 1H), 3.15 (dd, *J* = 14.9, 9.0 Hz, 1H), 2.61 – 2.50 (m, 2H), 2.00 (ddt, *J* = 9.2, 6.9, 4.2 Hz, 1H), 1.66 – 1.18 (m, 12H), 1.14 (s, 9H), 0.98 – 0.76 (m, 18H). ^{13}C NMR (126 MHz, CD_3OD-d_4) δ 174.25, 172.69, 172.46, 172.11, 172.05, 170.59, 136.69, 127.16, 123.54, 121.21, 118.63, 118.15, 110.96, 109.20, 73.17, 60.88, 59.27, 54.77, 53.85, 53.15, 52.08, 51.48, 40.88, 39.70, 39.70, 39.03, 35.88, 26.54, 26.43, 26.34, 26.31, 25.82, 24.62, 24.36, 21.59, 21.45, 21.38, 21.08, 14.93, 10.81.

Synthesis of H_2N-D-Leu-Ile-Ile-D-Orn(Boc)-Trp(Boc)-Leu-COOH (12a)

SPPS approach was used to prepare **12a** according to the general procedure described under section S1. The synthesis was initiated from a resin loaded with Fmoc- Leu-OH (0.3 g, 0.3 mmol). Crude % yield (104%), ESI-MS m/z calcd for $C_{50}H_{82}N_8O_{11}$: 970.61; found: 970.00 $[M-H]^-$.

Synthesis of cyclo(D-Leu-Ile-Ile-D-Orn(Boc)-Trp(Boc)-Leu) (12b)

Compound **12b** was obtained through a macrocyclization of **12a** (0.44 g, 0.46 mmol) with HATU (0.52 g, 1.37 mmol), HOBt (0.18 g, 1.37 mmol) and DIPEA (0.79 mL, 4.6 mmol) according to the procedure described in section S2. The crude was purified by a column chromatography using a silica gel as a stationary phase and chloroform/methanol (49:1) as a mobile phase to give a white amorphous solid mass. Yield (54%), ESI-MS m/z calcd for $C_{50}H_{80}N_8O_{10}$: 952.60; found: 953.58 $[M+H]^+$. R_f = 0.09 in $CHCl_3$/MeOH (49:1),

Synthesis of cyclo(D-Leu-Ile-Ile-D-Orn-Trp-Leu) (12c)

Compound **12b** (0.21 g, 0.22 mmol) was treated with 4 mL of TFA/DCM (1:1) for 1 h. The cleaving solution was removed under a strong pressure and the residue was purified by column chromatography using a silica gel as a stationary phase and chloroform/methanol/ammonia solution (85:15:1) as a mobile phase to give a white amorphous solid mass. Yield (70%), mp 165 °C, UPLC-MS (UV) purity: 99.9%, RT 0.92 min, ESI-MS m/z calcd for $C_{40}H_{64}N_8O_6$: 752.49; found: 753.50 $[M+H]^+$. ^{1}H NMR (400 MHz, CD_3OD-d_4) δ 7.60 (d, *J* = 7.9 Hz, 1H), 7.33 (d, *J* = 8.1 Hz, 1H), 7.15 (s, 1H), 7.12 – 7.04 (m, 1H), 7.03 – 6.94 (m, 1H), 4.61 (dd, *J* = 10.6, 3.7 Hz, 1H), 4.55 (t, *J* = 7.4 Hz, 1H), 4.43 (t, *J* = 7.4 Hz, 1H), 4.36 (d, *J* = 8.0 Hz, 1H), 4.12 (d, *J* = 5.3 Hz, 1H), 3.89 (dd, *J* = 8.5, 6.5 Hz, 1H), 3.51 (dd, *J* = 14.8, 3.7 Hz, 1H), 3.03 (dd, *J* = 14.8, 10.7 Hz, 1H), 2.53 – 2.35 (m, 2H), 1.97 (ddd, *J* = 16.1, 8.3, 4.4 Hz, 1H), 1.91 – 1.79 (m, 1H), 1.73 – 1.40 (m, 9H), 1.32 – 1.05 (m, 4H), 1.03 – 0.83 (m, 24H), 0.81 – 0.70 (m, 1H). ^{13}C NMR (101 MHz, CD_3OD-d_4) δ 173.21, 173.02, 172.60, 172.35, 171.66, 171.47, 136.75, 127.03, 123.46, 121.18, 118.55, 118.02, 110.98, 109.86, 59.26, 56.58, 54.51, 54.38, 51.66, 51.34, 40.45, 39.70, 38.99, 36.99, 35.97, 27.02, 26.59, 25.69, 24.76, 24.75, 24.51, 24.41, 21.91, 21.61, 21.25, 20.86, 14.88, 14.15, 10.59, 9.88.

Preparation of H_2N-D-Leu-Ile-Asn(Trt)-D-Arg(Pbf)-Trp(Boc)-Leu-COOH (13a)

SPPS approach was used to prepare **13a** according to the general procedure described under section S1. The synthesis was initiated from a resin loaded with Fmoc- Leu-OH (0.3 g, 0.3 mmol). Crude % yield (96%), ESI-MS m/z calcd for $C_{75}H_{99}N_{11}O_{13}S$: 1407.73; found: 1408.89 $[M+H]^+$.

Synthesis of cyclo (D-Leu-Ile-Asn(Trt)-D-Arg (Pbf)-Trp(Boc)-Leu) (13b)

Compound **13b** was obtained through a macrocyclization of **13a** (0.521 g, 0.37 mmol) with HATU (0.42 g, 1.1 mmol), HOBt (0.15 g, 1.1 mmol) and DIPEA (0.65 mL, 3.7 mmol) according to the procedure described in section S2. The crude was purified by a column chromatography using a silica gel as a stationary phase and chloroform/methanol (49:1) as a mobile phase to give a white amorphous solid mass. Yield (70%), ESI-MS: m/z calcd for $C_{76}H_{99}N_{11}O_{12}S$: 1389.72; found: 1412.84 $[M+Na]^+$. R_f = 0.77 in $CHCl_3$/MeOH (4:1),

Synthesis of cyclo(D-Leu-Ile-Asn-D-Arg-Trp-Leu) (13c)

Compound **13b** (0.32 g, 0.23 mmol) was treated with 5 mL of TFA/TIPS/H_2O (95:2.5/2.5/) for 3 h. The cleaving solution was removed under a strong pressure and the product was washed with cold ether and kept under strong vacuum to give a white amorphous solid mass. Yield (70%), mp 271-273 °C, UPLC-MS (UV) purity: 96%, RT 0.79 min, ESI-MS m/z calcd for $C_{39}H_{61}N_{11}O_7$: 795.48; found: 796.488 $[M+H]^+$. ^1H NMR (400 MHz, DMSO-d_6) δ 11.08 (s, 1H), 8.42 (d, *J* = 8.0 Hz, 1H), 8.25 (t, *J* = 6.6 Hz, 2H), 7.96 (s, 1H), 7.85 (d, *J* = 6.4 Hz, 1H), 7.46 (dd, *J* = 16.8, 7.8 Hz, 4H), 7.35 (d, *J* = 8.1 Hz, 1H), 7.31 – 7.17 (m, 3H), 7.13 (d, *J* = 2.3 Hz, 1H), 7.01 (t, *J* = 7.5 Hz, 1H), 6.97 – 6.88 (m, 2H), 4.57 (q, *J* = 6.7 Hz, 1H), 4.42 (q, *J* = 7.2 Hz, 1H), 4.35 – 4.21 (m, 2H), 4.12 – 3.96 (m, 2H), 3.27 – 3.18 (m, 1H), 2.91 (dd, *J* = 14.6, 9.7 Hz, 3H), 2.57 (d, *J* = 6.5 Hz, 2H), 1.95 (dq, *J* = 11.0, 5.7, 5.0 Hz, 1H), 1.63 – 1.12 (m, 12H), 0.97 – 0.70 (m, 16H). ^{13}C NMR (101 MHz, DMSO-d_6) δ 173.57, 172.05, 171.94, 171.92, 171.28, 170.91, 170.82, 157.19, 136.65, 127.32, 124.27, 121.14, 118.62, 118.32, 112.01, 110.35, 67.46, 65.35, 58.55, 55.42, 53.14, 52.15, 51.09, 49.92, 42.29, 37.90, 35.96, 27.69, 27.50, 25.04, 24.88, 24.54, 24.35, 23.03, 22.97, 22.50, 16.12, 15.61, 12.20.

Synthesis of H_2N-Val-Asn(Trt)-D-Orn(Boc)-Phe(4-Cl)-Leu-D-Leu-COOH (14a)

SPPS approach was used to prepare **14a** according to the general procedure described under section S1. The synthesis was initiated from a resin loaded with Fmoc-D-Leu-OH (0.3 g, 0.3 mmol). Crude % yield (98%), ESI-MS m/z calcd for $C_{59}H_{79}N_8O_{10}$: 1094.56; found: 1117.21 $[M+Na]^+$.

Synthesis of cyclo(D-Phe(4-Cl)-D-Orn(Boc)-Asn(Trt)-Val-D-Leu-Leu) (14b)

Compound **14b** was obtained through a macrocyclization of **14a** (0.33 g, 0.3 mmol) with HATU (0.34 g, 0.9 mmol), HOBt (0.12 g, 0.9 mmol) and DIPEA (0.52 mL, 3.0 mmol) according to the procedure described in section S2. It was purified by a column chromatography using a silica gel as a stationary phase and chloroform/methanol (97:3) as a mobile phase to give a white amorphous solid mass. Yield (43%), ESI-MS m/z calcd for $C_{59}H_{77}ClN_8O_9$: 1076.55; found: 1099.27 $[M+Na]^+$ R_f = 0.14 in $CHCl_3$/MeOH (98:2).

Synthesis of cyclo(D-Phe(4-Cl)-D-Orn-Asn-Val-D-Leu-Leu) (14c)

Compound **14b** (0.12 g, 0.11 mmol) was treated with 2.5 mL of TFA/TIPS/H_2O (95:2.5/2.5/) for 3 h. The cleaving solution was removed under a strong pressure and the residue was purified by column chromatography using a silica gel as a stationary phase and chloroform/methanol/ammonia solution (80:20:1) as a mobile phase to give a white amorphous solid mass. Yield (91%), mp 255-258 °C, UPLC-MS (UV) purity: 99.9%, RT 0.81 min, ESI-MS m/z calcd for $C_{35}H_{55}$ ClN_8O_7: 734.39; found: 735.39 $[M+H]^+$. ^{1}H NMR (400 MHz, CD_3OD-d_4) δ 7.32 – 7.26 (m, 2H), 7.21 – 7.16 (m, 2H), 4.68 (dd, J = 7.6, 6.2 Hz, 1H), 4.41 (ddd, J = 11.7, 8.6, 6.5 Hz, 3H), 4.20 (dd, J = 8.6, 5.3 Hz, 1H), 4.14 (d, J = 5.2 Hz, 1H), 3.20 (dd, J = 13.7, 6.9 Hz, 1H), 3.07 (dd, J = 13.7, 8.5 Hz, 1H), 2.91 – 2.83 (m, 2H), 2.81 (d, J = 7.6 Hz, 1H), 2.74 (dd, J = 15.6, 6.2 Hz, 1H), 2.28 – 2.19 (m, 1H), 1.69 – 1.54 (m, 8H), 1.39 (ddd, J = 13.4, 8.9, 5.8 Hz, 1H), 1.28 (dt, J = 13.5, 6.9 Hz, 1H), 1.04 – 0.80 (m, 18H). ^{13}C NMR (101 MHz, CD_3OD-d_4) δ 173.94, 173.37, 172.75, 172.33, 172.06, 171.92, 170.84, 136.21, 132.15, 130.58, 128.18, 59.15, 55.06, 53.01, 52.27, 51.21, 50.53, 39.65, 39.60, 38.57, 35.97, 35.47, 30.21, 27.95, 24.55, 24.29, 23.32, 21.73, 21.02, 20.85, 18.24, 16.40.

Synthesis of H_2N-D-Leu-Val-Asn (Trt)-D-Orn(Boc)- Phe(4-OMe)-Leu- COOH (15a)

SPPS approach was used to prepare **15a** according to the general procedure described under section S11. The synthesis was initiated from a resin loaded with Fmoc- Leu-OH (0.31 g, 0.3 mmol). Crude % yield (96%), ESI-MS m/z calcd for $C_{60}H_{82}N_8O_{11}$:1090.61; found: 1089.59 $[M-H]^-$.

Synthesis of cyclo(D-Leu-Val-Asn(Trt)-D-Orn(Boc)-Phe(4-OMe)-Leu) (15b)

Compound **15b** was obtained through a macrocyclization of **15a** (0.35 g, 0.32 mmol) with HATU (0.37 g, 0.96 mmol), HOBt (0.13 g, 0.96 mmol) and DIPEA (0.56 mL, 3.2 mmol) according to the procedure described in section S2. The crude was purified by a column chromatography using a silica gel as a stationary phase and chloroform/methanol (49:1) as a mobile phase to give a white amorphous solid mass. Yield (41%), ESI-MS m/z calcd for $C_{60}H_{80}N_8O_{10}$:1072.60; found: 1095.60$[M+Na]^+$. R_f = 0.09 in $CHCl_3$/MeOH (49:1)

Synthesis of cyclo(D-Leu-Val-Asn-D-Orn-Phe(4-OMe)-Leu) (15c)

Compound **15b** (0.06 g, 0.06 mmol) was treated with 3 mL of TFA/TIPS/H_2O (95:2.5/2.5/) for 3 h. The cleaving solution was removed under a strong pressure and the residue was purified by column chromatography using a silica gel as a stationary phase and chloroform/methanol/ammonia solution (85:15:1) as a mobile phase to give a white amorphous solid mass. Yield (82%), mp 243-245 °C, UPLC-MS (UV) purity: 99.9%, RT 0.74 min, ESI-MS m/z calcd for $C_{36}H_{58}N_8O_8$: 730.44; found: 731.44 $[M+H]^+$. ^{1}H NMR (400 MHz, CD_3OD-d_4) δ 7.17 – 7.08 (m, 2H), 6.87 – 6.79 (m, 2H), 4.68 (dd, *J* = 5.7, 4.6 Hz, 1H), 4.53 – 4.38 (m, 3H), 4.31 (dd, *J* = 9.9, 5.1 Hz, 1H), 4.05 (d, *J* = 4.6 Hz, 1H), 3.75 (s, 3H), 3.17 – 3.01 (m, 2H), 2.86 (dd, *J* = 14.0, 10.2 Hz, 1H), 2.80 – 2.69 (m, 3H), 2.36 – 2.27 (m, 1H), 1.87 – 1.80 (m, 1H), 1.74 – 1.35 (m, 9H), 1.04 – 0.87 (m, 18H). ^{13}C NMR (101 MHz, CD_3OD-d_4) δ 175.20, 173.39, 172.41, 172.38, 172.15, 172.02, 171.84, 158.68, 129.89, 128.61, 113.52, 60.26, 56.81, 54.22, 52.12, 51.99, 51.22, 50.04, 40.89, 39.53, 39.21, 36.34, 35.68, 29.16, 26.33, 24.73, 24.70, 24.52, 22.80, 21.66, 21.57, 21.36, 21.07, 18.11, 16.95, 16.49.

Synthesis of H_2N-D-Phe(4-Cl)-Val-Asn(Trt)-D-Orn(Boc)-Trp(Boc)-Leu-COOH (16a)

SPPS approach was used to prepare **16a** according to the general procedure described under section S1. The synthesis was initiated from a resin loaded with Fmoc- Leu-OH (0.143 g, 0.1 mmol). Crude % yield (70%), ESI-MS m/z calcd for $C_{70}H_{88}ClN_9O_{12}$: 1267.61; found: 1266.83 $[M-H]^-$

Synthesis of cyclo(D-Phe(4-Cl)-Val-Asn(Trt)-D-Orn(Boc)-Trp(Boc)-Leu) (16b)

Compound **16b** was obtained through a macrocyclization of **16a** (0.09 g, 0.07 mmol) with HATU (0.08 g, 0.2 mmol), HOBt (0.03 g, 0.2 mmol) and DIPEA (0.12 mL, 0.68 mmol) according to the procedure described in section S2. The crude was purified by a column chromatography using a silica gel as a stationary phase and chloroform/methanol (49:1) as a mobile phase to give a white amorphous solid mass. Yield (53%), ESI-MS m/z calcd for $C_{70}H_{86}ClN_9O_{11}$:1249.60; found: 1272.65 $[M+Na]^+$. R_f = 0.08 in $CHCl_3$/MeOH (49:1)

Synthesis of cyclo(D-Phe(4-Cl)-Val-Asn-D-Orn-Trp-Leu) (16c)

Compound **16b** (0.05 g, 0.04 mmol) was treated with 1.5 mL of TFA/TIPS/H_2O (95:2.5/2.5/) for 3 h. The cleaving solution was removed under a strong pressure and the product was washed with cold ether and kept under strong vacuum to give a white amorphous solid mass. Yield (75%), mp 280-282 °C, UPLC-MS (UV) purity: 91%, RT 0.82 min, ESI-MS m/z calcd for $C_{40}H_{54}ClN_9O_7$: 807.38; found: 808.378 $[M+H]^+$. ^1H NMR (400 MHz, CD_3OD-d_4) δ 7.58 (dt, *J* = 7.8, 1.0 Hz, 1H), 7.32 (dt, *J* = 8.2, 1.0 Hz, 1H), 7.30 – 7.25 (m, 2H), 7.24 – 7.18 (m, 2H), 7.12 (s, 1H), 7.09 (ddd, *J* = 8.2, 6.9, 1.2 Hz, 1H), 7.02 (ddd, *J* = 7.9, 7.1, 1.1 Hz, 1H), 4.70 – 4.51 (m, 3H), 4.45 – 4.35 (m, 2H), 3.77 (d, *J* = 4.7 Hz, 1H), 3.24 (dd, *J* = 14.7, 5.3 Hz, 1H), 3.16 – 3.06 (m, 2H), 3.03 – 2.92 (m, 2H), 2.84 (dq, *J* = 8.7, 6.2 Hz, 2H), 2.70 (dd, *J* = 16.8, 4.2 Hz, 1H), 2.16 – 2.08 (m, 1H), 1.91 – 1.28 (m, 7H), 0.97 – 0.68 (m, 12H). ^{13}C NMR (101 MHz, CD_3OD-d_4) δ 174.00, 173.40, 172.62, 172.40, 172.34, 172.14, 171.74, 135.10, 132.51, 130.59, 128.22, 127.05, 123.32, 121.11, 118.56, 117.92, 110.85, 109.09, 93.19, 60.56, 56.13, 54.94, 51.54, 51.11, 49.97, 40.51, 38.78, 36.05, 35.39, 29.00, 27.25, 26.05, 24.58, 23.33, 21.56, 21.23, 17.76, 16.38.

Synthesis of H_2N-Asn(Trt)-D-Orn(Boc)-Trp(Boc)-D-Phe(4-Cl)-D-Leu-Val-COOH (17a)

SPPS approach was used to prepare **17a** according to the general procedure described under section S1. The synthesis was initiated from a resin loaded with Fmoc-Val-OH (0.29 g, 0.25 mmol). Crude % yield (80%), ESI-MS m/z calcd for $C_{69}H_{86}ClN_9O_{12}$: 1267.61; found: 1290.61 $[M+Na]^+$.

Synthesis of cyclo(Asn(Trt)-D-Orn(Boc)-Trp(Boc)-D-Phe(4-Cl)-D-Leu-Val) (17b)

Compound **17b** was obtained through a macrocyclization of **17a** (0.25 g, 0.2 mmol) with HATU (0.23 g, 0.6 mmol), HOBt (0.08 g 0.6 mmol) and DIPEA (0.35 mL, 2.0 mmol) according to the procedure described in section S2. It was purified by a column chromatography using a silica gel as a stationary phase and chloroform/methanol (98:2) as a mobile phase to give a white amorphous solid mass. Yield (54%), ESI-MS m/z calcd for $C_{69}H_{84}ClN_9O_{11}$: 1249.60; found: 1272.81 $[M+Na]^+$.

Synthesis of cyclo(Asn-D-Orn-Trp-D-Phe (4-Cl)-D-Leu-Val) (17c)

Compound **17b** (0.12 g, 0.09 mmol) was treated with 3.0 mL of TFA/TIPS/H_2O (95:2.5/2.5/) for 3 h. The cleaving solution was removed under a strong pressure and the residue was purified by column chromatography using a silica gel as a stationary phase and chloroform/methanol/ammonia solution (85:15:1) as a mobile phase to give a white amorphous solid mass. Yield (80%), mp 240-243°C, UPLC-MS (UV) purity: 99.9%, RT 0.82 min, ESI-MS m/z calcd for $C_{40}H_{54}ClN_9O_7$: 807.38; found: 808.38 $[M+H]^+$ ^1H NMR (400 MHz, CD_3OD-d_4) δ 7.55 (dt, J = 7.9, 1.0 Hz, 1H), 7.39 (dt, J = 8.2, 0.9 Hz, 1H), 7.14 (ddd, J = 8.2, 7.0, 1.1 Hz, 1H), 7.07 – 7.02 (m, 3H), 7.00 (s, 1H), 6.75 – 6.69 (m, 2H), 4.71 – 4.62 (m, 2H), 4.55 – 4.43 (m, 3H), 3.86 (d, J = 5.7 Hz, 1H), 3.11 (dd, J = 14.0, 9.1 Hz, 1H), 3.00 (ddd, J = 14.0, 6.3, 0.9 Hz, 1H), 2.96 – 2.81 (m, 3H), 2.81 – 2.76 (m, 2H), 2.72 (dd, J = 14.1, 4.7 Hz, 1H), 2.22 – 2.12 (m, 1H), 1.92 – 1.83 (m, 1H), 1.77 (ddd, J = 14.1, 9.5, 4.9 Hz, 1H), 1.69 – 1.52 (m, 3H), 1.47 – 1.40 (m, 1H), 1.38 – 1.34 (m, 1H), 1.05 – 0.84 (m, 12H). ^{13}C NMR (101 MHz, CD_3OD-d_4) δ 174.07, 173.57, 172.71, 172.04, 171.68, 171.48, 171.16, 136.63, 135.11, 132.18, 130.28, 128.04, 127.10, 123.31, 121.13, 118.49, 117.91, 111.15, 109.08, 61.30, 55.19, 54.57, 51.66, 51.44, 50.78, 41.45, 38.84, 35.68, 35.36, 29.42, 27.61, 26.28, 24.23, 23.29, 21.88, 21.23, 18.04, 17.24.

Synthesis of H_2N-D-Phe(4-Cl)-Ile-D-Orn(Boc)-D-Orn(Boc)-Trp(Boc)-Leu-COOH (18a)

SPPS approach was used to prepare **18a** according to the general procedure described under section S1. The synthesis was initiated from a resin loaded with Fmoc- Leu-OH (0.43 g, 0.44 mmol). Crude % yield (99%), ESI-MS m/z calcd for $C_{57}H_{86}ClN_9O_{13}$: 1139.60; found: 1140.62 $[M+H]^+$.

Synthesis of cyclo(D-Phe (4-Cl)-Ile-D-Orn(Boc)-D-Orn(Boc)-Trp(Boc)-Leu) (18b)

Compound **18b** was obtained through a macrocyclization of **18a** (0.3 g, 0.26 mmol) with HATU (0.3 g, 0.79 mmol), HOBt (0.11 g, 0.79 mmol) and DIPEA (0.46 mL, 2.63 mmol) according to the procedure described in section S2. The crude was purified by a column chromatography using a silica gel as a stationary phase and chloroform/methanol (49:1) as a mobile phase to give a white amorphous solid mass. Yield (55%), ESI-MS m/z calcd for $C_{57}H_{84}ClN_9O_{12}$: 1121.59; found, 1144.51 $[M+Na]^+$. R_f = 0.26 in $CHCl_3$/MeOH (49:1)

Synthesis of cyclo(D-Phe (4-Cl)-Ile-D-Orn-D-Orn-Trp-Leu) (18c)

Compound **18b** (0.11 g, 0.1 mmol) was treated with 3 mL of TFA/TIPS/H_2O (95:2.5/2.5/) for 1 h. The cleaving solution was removed under a strong pressure and the residue was purified by column chromatography using a silica gel as a stationary phase and chloroform/methanol/ammonia solution (70:30:1) as a mobile phase to give a white amorphous solid mass. Yield (50%), mp 290-292 °C, UPLC-MS (UV) purity: 96%, RT 0.73 min, ESI-MS m/z calcd for $C_{42}H_{60}ClN_9O_6$: 821.44; found: 822.44 $[M+H]^+$. ^{1}H NMR (400 MHz, DMSO-d_6) δ 10.86 (s, 1H), 8.60 (d, *J* = 7.9 Hz, 1H), 8.30 (d, *J* = 6.1 Hz, 1H), 8.14 (d, *J* = 7.7 Hz, 1H), 7.94 (d, *J* = 8.5 Hz, 1H), 7.51 (d, *J* = 7.7 Hz, 1H), 7.33 – 7.24 (m, 3H), 7.21 (s, 1H), 7.19 – 7.13 (m, 2H), 7.07 – 7.01 (m, 1H), 7.00 – 6.93 (m, 1H), 4.61 – 4.49 (m, 1H), 4.35 – 4.15 (m, 3H), 3.96 (dt, *J* = 10.1, 4.9 Hz, 1H), 3.89 – 3.77 (m, 1H), 3.22 (d, *J* = 3.6 Hz, 1H), 2.96 – 2.81 (m, 2H), 2.78 – 2.69 (m, 1H), 2.44 (d, *J* = 6.7 Hz, 2H), 2.16 (t, *J* = 7.0 Hz, 2H), 1.81 – 1.14 (m, 13H), 1.04 – 0.91 (m, 1H), 0.86 – 0.54 (m, 12H). ^{13}C NMR (101 MHz, DMSO-d_6) δ 172.03, 171.91, 171.77, 171.68, 171.47, 169.87, 136.66, 131.51, 131.47, 128.38, 127.23, 124.52, 121.31, 118.74, 118.36, 111.79, 110.33, 67.45, 57.78, 56.23, 54.48, 53.97, 52.89, 51.03, 41.39, 41.06, 40.65, 40.60, 37.84, 34.76, 29.99, 29.40, 27.98, 27.58, 26.59, 25.56, 24.69, 24.63, 23.45, 21.88, 15.46, 10.58

Synthesis of H_2N-Asn(Trt)-D-Orn(Boc)-Trp(Boc)-Ile-D-Leu-Val-COOH (19a)

SPPS approach was used to prepare **19a** according to the general procedure described under section S1. The synthesis was initiated from a resin loaded with Fmoc-Val-OH (0.29 g, 0.25 mmol). Crude % yield (89%), ESI-MS m/z calcd for $C_{66}H_{89}N_9O_{12}$: 1199.66; found: 1200.59 $[M+H]^+$.

Synthesis of cyclo(Asn(Trt)-D-Orn(Boc)-Trp(Boc)-Ile-D-Leu-Val) (19b)

Compound **19b** was obtained through a macrocyclization of **19a** (0.27 g, 0.22 mmol) with HATU (0.25 g, 0.67 mmol), HOBt (0.09 g 0.67 mmol) and DIPEA (0.39 mL, 6.65 mmol) according to the procedure described in section S2. It was purified by a column chromatography using a silica gel as a stationary phase and chloroform/methanol (98:2) as a mobile phase to give a white amorphous solid mass. Yield (47%), ESI-MS m/z calcd for $C_{66}H_{87}N_9O_{11}$: 1181.65; found: 1204.56 $[M+Na]^+$. R_f = 0.08 in $CHCl_3$/MeOH (98:2),

Synthesis of cyclo(Asn-D-Orn-Trp-Ile-D-Leu-Val) (19c)

Compound **19b** (0.11 g, 0.09 mmol) was treated with 2.5 mL of TFA/TIPS/H_2O (95:2.5/2.5/) for 3 h. The cleaving solution was removed under a strong pressure and the residue was washed with cold ether to give a white amorphous solid mass. Yield (96%), mp 175-177 °C, UPLC-MS (UV) purity: 95%, RT 0.74 min, ESI-MS m/z calcd for $C_{37}H_{57}N_9O_7$: 739.44; found: 740.44 $[M+H]^+$. ^{1}H NMR (400 MHz, DMSO-d_6) δ 10.80 (d, J = 2.5 Hz, 2H), 8.44 (d, J = 4.8 Hz, 1H), 8.34 (dd, J = 16.0, 8.2 Hz, 2H), 7.56 (ddd, J = 22.1, 13.8, 7.2 Hz, 5H), 7.31 (d, J = 8.2 Hz, 2H), 7.12 (d, J = 2.3 Hz, 1H), 7.07 – 7.00 (m, 1H), 6.99 – 6.92 (m, 2H), 4.61 (dt, J = 8.4, 6.2 Hz, 1H), 4.37 – 4.12 (m, 4H), 4.05 (dd, J = 8.0, 4.1 Hz, 1H), 3.18 (dd, J = 14.6, 4.5 Hz, 1H), 2.96 (dd, J = 14.8, 10.4 Hz, 1H), 2.71 – 2.56 (m, 3H), 2.36 – 2.23 (m, 1H), 1.82 – 1.18 (m, 10H), 1.01 – 0.68 (m, 18H). ^{13}C NMR (101 MHz, DMSO-d_6) δ 173.99, 172.20, 171.31, 171.27, 171.26, 171.25, 170.92, 136.57, 127.46, 124.04, 121.33, 118.79, 118.59, 111.80, 110.62, 58.65, 56.16, 55.88, 52.60, 52.05, 50.07, 38.75, 38.20, 37.62, 29.24, 27.99, 27.09, 25.56, 25.07, 24.53, 23.66, 22.86, 22.50, 19.62, 17.29, 15.35, 11.66.

Synthesis of H_2N-Asn(Trt)-D-Orn(Boc)-Trp(Boc)-Leu-Ile-Val-COOH (20a)

SPPS approach was used to prepare **20a** according to the general procedure described under section S1. The synthesis was initiated from a resin loaded with Fmoc-Val-OH (0.30 g, 0.25 mmol). Crude % yield (85%), ESI-MS m/z calcd for $C_{66}H_{89}N_9O_{12}$: 1199.66; found: 1222.71 $[M+Na]^+$.

Synthesis of cyclo(Asn(Trt)-D-Orn(Boc)-Trp(Boc)-Leu-Ile-Val) (20b)

Compound **20b** was obtained through a macrocyclization of **20a** (0.26 g, 0.21 mmol) with HATU (0.24 g, 0.64 mmol), HOBt (0.07 g 0.64 mmol) and DIPEA (0.37 mL, 6.36 mmol) according to the procedure described in section S2. It was purified by a column chromatography using a silica gel as a stationary phase and chloroform/methanol (98:2) as a mobile phase to give a white amorphous solid mass. Yield (47%), ESI-MS m/z calcd for $C_{66}H_{87}N_9O_{11}$: 1181.65; found: 1204.65 $[M+Na]^+$. R_f = 0.1 in $CHCl_3$/MeOH (97:3).

Synthesis of cyclo(Asn-D-Orn-Trp-Leu-Ile-Val) (20c)

Compound **20b** (0.13 g, 0.11 mmol) was treated with 2.5 mL of TFA/TIPS/H_2O (95:2.5/2.5) for 3 h. The cleaving solution was removed under a strong pressure and the residue was purified by column chromatography using a silica gel as a stationary phase and chloroform/methanol/ammonia solution (80:20:1) as a mobile phase to give a white amorphous solid mass. that contains a mixture two stereoisomers (3:1 ratio) . Yield (72%), mp 166-169 °C, UPLC-MS (UV) purity: 95%, RT 0.77 min, ESI-MS m/z calcd for $C_{37}H_{57}N_9O_7$: 739.44; found: 740.44$[M+H]^+$. ^1H NMR (400 MHz, CD_3OD-d_4) δ 7.63 (dd, J = 7.9, 3.7 Hz, 2H), 7.36 – 7.29 (m, 2H), 7.15 (d, J = 5.6 Hz, 1H), 7.12 – 7.05 (m, 2H), 7.05 – 6.98 (m, 2H), 4.71 – 4.64 (m, 2H), 4.60 (dd, J = 10.0, 4.6 Hz, 1H), 4.49 (dd, J = 11.4, 3.9 Hz, 1H), 4.43 (t, J = 7.8 Hz, 1H), 4.34 (dd, J = 8.4, 5.2 Hz, 1H), 4.13 (t, J = 7.4 Hz, 1H), 4.08 (d, J = 5.2 Hz, 1H), 3.76 (d, J = 8.1 Hz, 1H), 3.43 – 3.34 (m, 1H), 3.11 (ddd, J = 15.2, 9.4, 6.1 Hz, 2H), 3.02 (dd, J = 16.1, 4.7 Hz, 1H), 2.90 – 2.78 (m, 3H), 2.77 – 2.61 (m, 3H), 2.20 (ddt, J = 20.8, 14.4, 6.4 Hz, 2H), 1.92 – 1.38 (m, 14H), 1.08 – 0.77 (m, 24H). ^{13}C NMR (101 MHz, CD_3OD-d_4) δ 174.64, 173.93, 173.80, 173.73, 173.53, 173.52, 173.42, 172.52, 171.98, 171.95, 171.74, 136.71, 127.08, 127.05, 123.24, 123.16, 121.15, 121.12, 118.52, 117.93, 110.97, 110.92, 109.98, 109.46, 78.03, 62.28, 59.84, 59.78, 58.57, 55.84, 55.31, 53.64, 52.32, 52.13, 50.96, 50.44, 40.22, 39.43, 38.82, 38.54, 36.33, 34.87, 29.96, 28.64, 26.87, 26.54, 25.38, 24.55, 23.14, 22.34, 22.29, 21.87, 19.96, 19.80, 18.67, 18.38, 17.12, 16.21, 14.64, 14.40, 9.81, 9.64.

Synthesis of H_2N- Trp(Boc)-D-Orn (Boc)-Asn(Trt)-Val-D-Leu-Leu-COOH (21a)

SPPS approach was used to prepare **21a** according to the general procedure described under section S1. The synthesis was initiated from a resin loaded with Fmoc-Leu-OH (0.63 g, 0.5 mmol). Crude % yield (92%), ESI-MS m/z calcd for $C_{66}H_{89}N_9O_{12}$:1199.66; found: 1198.95 $[M+Na]^+$.

Synthesis of cyclo(Trp (Boc)-D-Orn (Boc)-Asn(Trt)-Val-D-Leu-Leu) (21b)

Compound **21b** was obtained through a macrocyclization of **21a** (0.2 g, 0.17 mmol) with HATU (0.19 g, 0.51 mmol), HOBt (0.07 g, 0.51 mmol) and DIPEA (0.19 g, 1.7 mmol) in DMF (170 mL) according to the procedure described in section S2. It was purified by a column chromatography using a silica gel as a stationary phase and chloroform/methanol (97:3) as a mobile phase to give a white amorphous solid mass. Yield (50 %), ESI-MS m/z calcd for $C_{66}H_{87}N_9O_{11}$: 1181.65; found: 1180.97$[M-H]^-$. R_f = 0.11 in $CHCl_3$/MeOH (97:3).

Synthesis of cyclo(Trp-Val-Asn-D-Orn-D-Leu-Leu) (21c)

Compound **21b** (0.08g, 0.07 mmol) was treated with 3 mL of TFA/TIPS/H_2O (95:2.5/2.5/) for 3 h. The cleaving solution was removed under a strong pressure and the residue was purified by column chromatography using a silica gel as a stationary phase and chloroform/methanol/ammonia solution (80:20:1) as a mobile phase to give a white amorphous solid mass. Yield (89%), mp 232-235 °C, UPLC-MS (UV) purity: 97%, RT 0.78 min, ESI-MS m/z calcd for $C_{37}H_{57}N_9O_7$: 739.44; found: 740.44 $[M+H]^+$. ^{1}H NMR (400 MHz, CD_3OD-d_4) δ 7.62 (d, *J* = 7.9 Hz, 1H), 7.33 (d, *J* = 8.1 Hz, 1H), 7.14 – 7.04 (m, 2H), 7.00 (d, *J* = 7.3 Hz, 1H), 4.75 (t, *J* = 7.4 Hz, 1H), 4.61 (dd, *J* = 8.6, 4.2 Hz, 1H), 4.31 (d, *J* = 7.6 Hz, 1H), 4.25 – 4.12 (m, 2H), 4.02 (t, *J* = 7.5 Hz, 1H), 3.37 – 3.32 (m, 1H), 3.23 (dd, *J* = 14.3, 7.4 Hz, 1H), 2.84 (dd, *J* = 15.9, 4.2 Hz, 1H), 2.71 (dd, *J* = 15.8, 8.6 Hz, 1H), 2.55 (t, *J* = 7.2 Hz, 2H), 2.16 (h, *J* = 6.9 Hz, 1H), 1.68 – 1.20 (m, 10H), 1.01 – 0.77 (m, 18H). ^{13}C NMR (101 MHz, CD_3OD-d_4) δ 174.05, 173.44, 173.16, 172.49, 171.99, 171.50, 171.22, 136.56, 127.56, 123.09, 120.96, 118.28, 118.14, 110.87, 109.91, 58.01, 54.39, 53.42, 52.99, 52.16, 51.26, 39.77, 39.62, 39.36, 35.91, 31.59, 28.07, 26.76, 25.87, 24.59, 24.47, 22.25, 21.38, 21.32, 19.61, 17.99, 17.68.

Synthesis of H_2N-D-Leu-N-Me-Val-Asn(Trt)-D-Orn(Boc)-Trp(Boc)-Leu-COOH (22a)

SPPS approach was used to prepare **22a** according to the general procedure described under section S1. The synthesis was initiated from a resin loaded with Fmoc-Leu-OH (0.31 g, 0.3 mmol). Crude % yield (93%), ESI-MS m/z calcd for $C_{66}H_{89}N_9O_{12}$: 1213.68; found: 1214.83 $[M+H]^+$.

Synthesis of cyclo(D-Leu-N-Me-Val-Asn(Trt)-D-Orn(Boc)-Trp(Boc)-Leu) (22b)

Compound **22b** was obtained through a macrocyclization of **22a** (0.34 g, 0.28 mmol) with HATU (0.32 g, 0.84 mmol), HOBt (0.11 g, 0.84 mmol) and DIPEA (0.49 mL, 2.8 mmol) according to the procedure described in section S2. The crude was purified by a column chromatography using a silica gel as a stationary phase and chloroform/methanol (49:1) as a mobile phase to give a white amorphous solid mass. Yield (60%), ESI-MS m/z calcd for $C_{67}H_{89}N_9O_{11}$: 1195.67; found: 1218.70 $[M+Na]^+$. $R_f = 0.1$ in $CHCl_3$/MeOH (98:2).

Synthesis of cyclo(D-Leu-N-Me-Val-Asn-D-Orn-Trp-Leu) (22c)

Compound **22b** (0.24 g, 0.2 mmol) was treated with 2.5 mL of TFA/TIPS/H_2O (95:2.5/2.5/) for 3 h. The cleaving solution was removed under a strong pressure and the residue was purified by column chromatography using a silica gel as a stationary phase and chloroform/methanol/ammonia solution (80:20:1) as a mobile phase to give a white amorphous solid mass. Yield (71%), mp 92-95 °C, UPLC-MS (UV) purity: 98%, RT 0.78 min, ESI-MS m/z calcd for $C_{38}H_{59}N_9O_7$: 753.45; found: 754.45 $[M+H]^+$. ^{1}H NMR (400 MHz, CD_3OD-d_4) δ 7.59 (dt, *J* = 7.9, 1.0 Hz, 1H), 7.34 (dt, *J* = 8.1, 0.9 Hz, 1H), 7.18 (s, 1H), 7.09 (ddd, *J* = 8.2, 7.0, 1.2 Hz, 1H), 7.01 (ddd, *J* = 8.0, 7.1, 1.1 Hz, 1H), 4.74 – 4.63 (m, 3H), 4.61 – 4.49 (m, 2H), 4.38 (dd, *J* = 8.2, 5.7 Hz, 1H), 3.37 (ddd, *J* = 14.7, 4.4, 0.9 Hz, 1H), 3.12 (dd, *J* = 14.8, 9.7 Hz, 1H), 3.03 (s, 2H), 2.98 – 2.81 (m, 2H), 2.69 (dddd, *J* = 36.2, 12.6, 8.5, 6.3 Hz, 2H), 2.24 (dp, *J* = 9.6, 6.6 Hz, 1H), 1.72 – 1.20 (m, 10H), 1.10 – 0.81 (m, 18H). ^{13}C NMR (101 MHz, CD_3OD-d_4) δ 174.13, 173.87, 172.83, 172.61, 172.12, 171.41, 171.02, 136.68, 127.14, 123.44, 121.11, 118.56, 117.96, 110.98, 109.46, 65.46, 62.96, 55.69, 52.33, 51.20, 50.16, 41.18, 39.52, 38.63, 35.55, 31.08, 27.68, 27.27, 26.72, 24.47, 24.38, 23.03, 21.98, 21.88, 20.90, 20.85, 19.64, 18.66.

Synthesis of H_2N-Val-N-Me-Asn(Trt)-D-Orn(Boc)-Trp(Boc)-N-Me-Leu-D-Leu-COOH (23a)

SPPS approach was used to prepare **23a** according to the general procedure described under section S1. The synthesis was initiated from a resin loaded with Fmoc-D-Leu-OH (0.31 g, 0.3 mmol). Crude % yield (96%), ESI-MS m/z calcd for $C_{68}H_{93}N_9O_{12}$:1227.69; found: 1250.70 $[M+Na]^+$.

Synthesis of cyclo(Val-N-Me-Asn(Trt)-D-Orn(Boc)-Trp(Boc)-N-Me-Leu-D-Leu) (23b)

Compound **23b** was obtained through a macrocyclization of **23a** (0.38 g, 0.3 mmol) with HATU (0.34 g, 0.9 mmol), HOBt (0.12 g, 0.9 mmol) and DIPEA (0.52 mL, 3.0 mmol) according to the procedure described in section S2. The crude was purified by a column chromatography using a silica gel as a stationary phase and chloroform/methanol (49:1) as a mobile phase to give a white amorphous solid mass. Yield (80%), ESI-MS m/z calcd for $C_{68}H_{91}N_9O_{11}$: 1209.68; found: 1232.68 $[M+Na]^+$. R_f = 0.12 in $CHCl_3$/MeOH (99:1)

Synthesis of cyclo(Val-N-Me-Asn-D-Orn-Trp-N-Me-Leu-D-Leu) (23c)

Compound **23b** (0.29 g, 0.24 mmol) was treated with 2.5 mL of TFA/TIPS/H_2O (95:2.5/2.5/) for 3 h. The cleaving solution was removed under a strong pressure and the residue was purified by column chromatography using a silica gel as a stationary phase and chloroform/methanol/ammonia solution (85:15:1) as a mobile phase to give a white amorphous solid mass. Yield (60%), mp 93 °C, UPLC-MS (UV) purity: 97%, RT 0.8 min, ESI-MS m/z calcd for $C_{39}H_{61}N_9O_7$: 767.47; found: 768.47 $[M+H]^+$. ^1H NMR (400 MHz, CD_3OD-d_4) δ 7.53 (s, 0H), 7.50 (dt, *J* = 7.9, 0.9 Hz, 1H), 7.35 (dt, *J* = 8.2, 1.0 Hz, 1H), 7.10 (ddd, *J* = 8.1, 7.0, 1.2 Hz, 1H), 7.05 – 6.99 (m, 1H), 6.97 (s, 1H), 5.60 (dd, *J* = 10.1, 4.4 Hz, 1H), 4.93 (dd, *J* = 10.8, 4.4 Hz, 1H), 4.75 (dd, *J* = 11.7, 3.6 Hz, 1H), 4.52 (dq, *J* = 6.5, 4.7, 3.8 Hz, 2H), 4.44 (t, *J* = 10.2 Hz, 1H), 3.45 – 3.33 (m, 1H), 3.23 – 3.05 (m, 2H), 3.02 – 2.89 (m, 2H), 2.77 (s, 2H), 2.68 (s, 1H), 2.61 (s, 2H), 2.36 (dd, *J* = 15.8, 4.4 Hz, 1H), 2.11 (dp, *J* = 8.4, 6.6 Hz, 1H), 1.94 – 1.40 (m, 9H), 1.08 – 0.79 (m, 12H), 0.74 – 0.65 (m, 1H), 0.51 – 0.32 (m, 6H), -0.57 (ddd, *J* = 13.2, 9.9, 3.4 Hz, 1H). ^{13}C NMR (101 MHz, CD_3OD-d_4) δ 173.31, 172.72, 172.54, 172.26, 171.37, 168.46, 168.11, 136.56, 126.97, 123.16, 121.29, 118.71, 117.71, 111.23, 109.01, 58.16, 56.53, 55.24, 52.26, 51.43, 50.23, 41.92, 39.04, 36.91, 34.53, 29.84, 28.92, 28.36, 28.16, 27.28, 24.74, 24.15, 23.02, 21.95, 21.90, 21.64, 21.30, 19.66, 18.33, 18.10, 16.94.

Synthesis of H_2N-Trp(Boc)-N-Me-Leu-D-Leu-N-Me-Val-Asn(Trt)-D-Orn(Boc)-COOH (24a)

SPPS approach was used to prepare **24a** according to the general procedure described under section S1. The synthesis was initiated from a resin loaded with Fmoc- D-Orn(Boc)-OH (0.33 g, 0.3 mmol). Crude % yield (90%), ESI-MS m/z calcd for $C_{68}H_{93}N_9O_{12}$: 1227.69; found: 1250.79 $[M+Na]^+$.

Synthesis of cyclo (Trp(Boc)-N-Me-Leu-D-Leu-N-Me-Val-Asn(Trt)-D-Orn(Boc)) (24b)

Compound **24b** was obtained through a macrocyclization of **24a** (0.34 g, 0.27 mmol) with HATU (0.31 g, 0.82 mmol), HOBt (0.11 g, 0.82 mmol) and DIPEA (0.48 mL, 2.73 mmol) according to the procedure described in section S2. The crude was purified by a column chromatography using a silica gel as a stationary phase and chloroform/methanol (49:1) as a mobile phase to give a white amorphous solid mass. Yield (73%), ESI-MS m/z calcd for $C_{68}H_{91}N_9O_{11}$: 1209.68; found: 1232.72 $[M+Na]^+$. R_f = 0.06 in $CHCl_3$/MeOH (49:1)

Synthesis of cyclo(Trp-N-Me-Leu-D-Leu-N-Me-Val-Asn-D-Orn) (24c)

Compound **24b** (0.24 g, 0.2 mmol) was treated with 5 mL of TFA/TIPS/H_2O (95:2.5:2.5) for 3 h. The cleaving solution was removed under a strong pressure and the residue was purified by column chromatography using a silica gel as a stationary phase and chloroform/methanol/ammonia solution (80:20:1) as a mobile phase to give a white amorphous solid mass. Yield (60%), mp 85-87 °C, UPLC-MS (UV) purity: 99.9%, RT 0.82 min, ESI-MS m/z calcd for $C_{39}H_{61}N_9O_7$: 767.47; found: 768.47$[M+H]^+$. ^1H NMR (400 MHz, CD_3OD-d_4) δ 7.55 – 7.50 (m, 1H), 7.37 – 7.31 (m, 1H), 7.13 – 7.06 (m, 1H), 7.06 – 6.97 (m, 2H), 5.09 (dd, *J* = 10.6, 4.6 Hz, 1H), 4.76 – 4.71 (m, 1H), 4.58 – 4.43 (m, 3H), 3.79 (d, *J* = 10.5 Hz, 1H), 3.37 (dd, *J* = 13.5, 10.7 Hz, 1H), 3.16 – 3.08 (m, 1H), 3.07 (s, 3H), 3.02 – 2.88 (m, 3H), 2.77 (dd, *J* = 16.0, 8.3 Hz, 1H), 2.61 (s, 3H), 2.33 (dt, *J* = 10.5, 6.3 Hz, 1H), 1.88 – 1.44 (m, 9H), 1.39 – 1.28 (m, 1H), 1.02 – 0.83 (m, 12H), 0.43 (dd, *J* = 51.3, 6.6 Hz, 6H). ^{13}C NMR (101 MHz, CD_3OD-d_4) δ 174.21, 172.87, 172.82, 171.42, 170.85, 168.98, 136.54, 127.11, 123.28, 121.30, 118.72, 117.79, 111.27, 108.87, 58.42, 56.91, 51.87, 51.01, 49.97, 40.16, 38.95, 36.47, 34.66, 33.21, 33.19, 29.14, 28.37, 28.17, 26.28, 24.77, 24.35, 23.27, 21.66, 21.04, 20.04, 18.88, 18.82, 16.95, 16.78, 12.24.

Synthesis of H_2N-Trp(Boc)-Leu-N-Me-D-Leu-Val-N-Me-Asn(Trt)-D-Orn(Boc)-COOH (25a)

SPPS approach was used to prepare **25a** according to the general procedure described under section S1. The synthesis was initiated from a resin loaded with Fmoc- D-Orn(Boc)-OH (0.33 g, 0.3 mmol). Crude % yield (95%), ESI-MS m/z calcd for $C_{68}H_{93}N_9O_{12}$: 1227.69; found: 1250.74 $[M+Na]^+$.

Synthesis of cyclo(Trp(Boc)-Leu-N-Me-D-Leu-Val-N-Me-Asn(Trt)-D-Orn(Boc)) (25b)

25b was obtained through a macrocyclization of **25a** (0.34 g, 0.28 mmol) with HATU (0.32 g, 0.84 mmol), HOBt (0.11 g, 0.84 mmol) and DIPEA (0.49 mL, 2.79 mmol) according to the procedure described in section S2. The crude was purified by a column chromatography using a silica gel as a stationary phase and chloroform/methanol (49:1) as a mobile phase to give a white amorphous solid mass. Yield (60%), ESI-MS m/z calcd for $C_{68}H_{91}N_9O_{11}$: 1209.68; found: 1232.69 $[M+Na]^+$. R_f = 0.11 in $CHCl_3$/MeOH (49:1),

Synthesis of cyclo(Trp-Leu-N-Me-D-Leu-Val-N-Me-Asn-D-Orn) (25c)

25b (0.08g, 0.07 mmol) was treated with 3 mL of TFA/TIPS/H_2O (95:2.5:2.5) for 3 h. The cleaving solution was removed under a strong pressure and the residue was purified by column chromatography using a silica gel as a stationary phase and chloroform/methanol/ammonia solution (80:20:1) as a mobile phase to give a white amorphous solid mass. Yield (63%), mp 93-95 °C, UPLC-MS (UV) purity: 98%, RT 0.82 min, ESI-MS m/z calcd for $C_{39}H_{61}N_9O_7$: 767.47; found: 768.47 $[M+H]^+$. ^1H NMR (400 MHz, CD_3OD-d_4) δ 7.68 (d, *J* = 7.8 Hz, 1H), 7.37 – 7.29 (m, 1H), 7.18 (s, 1H), 7.14 – 7.07 (m, 1H), 7.06 – 6.97 (m, 1H), 5.26 (dd, *J* = 8.7, 6.9 Hz, 1H), 4.92 (d, *J* = 4.3 Hz, 1H), 4.68 (dd, *J* = 9.8, 5.5 Hz, 1H), 4.60 (q, *J* = 8.2, 7.5 Hz, 1H), 4.39 (dq, *J* = 8.4, 4.9, 3.9 Hz, 1H), 4.28 (dd, *J* = 8.3, 5.3 Hz, 1H), 3.47 (dd, *J* = 14.6, 5.5 Hz, 1H), 3.31 (d, *J* = 5.4 Hz, 3H), 3.15 (dd, *J* = 16.4, 5.3 Hz, 1H), 3.09 – 3.00 (m, 1H), 2.97 (d, *J* = 5.8 Hz, 3H), 2.89 – 2.82 (m, 1H), 2.77 – 2.61 (m, 2H), 2.11 (dq, *J* = 11.2, 6.6 Hz, 1H), 1.78 – 1.54 (m, 4H), 1.42 (dddd, *J* = 24.0, 18.0, 9.2, 5.6 Hz, 6H), 1.07 – 0.78 (m, 18H). ^{13}C NMR (101 MHz, CD_3OD-d_4) δ 174.12, 172.91, 172.74, 172.06, 172.02, 170.39, 170.15, 136.69, 127.00, 123.64, 121.06, 118.47, 118.07, 110.92, 109.77, 61.68, 54.55, 54.05, 53.75, 52.00, 48.95, 40.21, 38.65, 38.03, 36.58, 33.37, 30.75, 29.53, 28.18, 27.37, 24.56, 24.29, 23.25, 21.87, 21.71, 21.22, 21.12, 19.24, 15.73.

Supplementary tables

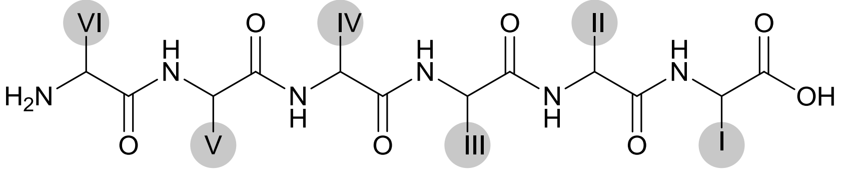

Table A. The linear precursors of the synthesized wollamide B analogues

Compound	I	II	III	IV	V	VI
**2a**	Ala	Trp (Boc)	D-Orn(Boc)	Asn(Trt)	Val	D-Leu
**3a**	Leu	Ala	D-Orn(Boc)	Asn(Trt)	Val	D-Leu
**4a**	Leu	Trp (Boc)	D-Orn(Boc)	Ala	Val	D-Leu
**5a**	Leu	Trp (Boc)	D-Orn(Boc)	Asn(Trt)	Ala	D-Leu
**6a**	Leu	Trp (Boc)	D-Orn(Boc)	Asn(Trt)	Val	D-Ala
**7a**	Leu	Trp (Boc)	D-Orn(Boc)	Asn(Trt)	*allo*-Ile	D-Leu
**8a**	Leu	Trp (Boc)	D-Orn(Boc)	Asn(Trt)	Ile	D-Leu
**9a**	Leu	Trp (Boc)	D-Orn(Boc)	Asn(Trt)	Met	D-Leu
**10a**	D-Leu	Leu	Trp(Boc)	D-Orn(Boc)	Asn(Trt)	Asp(Bzl)
**11a**	Leu	Trp (Boc)	D-Orn(Boc)	D-Orn(Boc)	Val	D-Leu
**12a**	D-Orn(Boc)	Ser (t-Bu)	Ile	D-Leu	Leu	Trp(Boc)
**13a**	D-Orn(Boc)	Ser (t-Bu)	Ile	D-Leu	Leu	Trp(Boc)
**14a**	Leu	Trp (Boc)	D-Orn(Boc)	Ile	Ile	D-Leu
**15a**	Leu	D-Leu	D-Phe(4-Cl)	D-Orn(Boc)	Asn(Trt)	Val
**16a**	Leu	D-Phe (4-OMe)	D-Orn(Boc)	Asn(Trt)	Val	D-Leu
**17a**	Val	D-Leu	D-Phe(4-Cl)	Trp(Boc)	D-Orn(Boc)	Asn(Trt)
**18a**	Leu	Trp (Boc)	D-Orn(Boc)	Asn(Trt)	Val	D-Phe (4-Cl)
**19a**	Leu	Trp (Boc)	D-Orn(Boc)	D-Orn(Boc)	Ile	D-Phe (4-Cl)
**20a**	Val	D-Leu	Ile	Trp(Boc)	D-Orn(Boc)	Asn(Trt)
**21a**	Val	Ile	Leu	Trp(Boc)	D-Orn(Boc)	Asn(Trt)
**22a**	Leu	Trp (Boc)	D-Arg(Pbf)	Asn(Trt)	Ile	D-Leu
**23a**	Leu	Trp (Boc)	D-Orn(Boc)	Asn(Trt)	N-Me-Val	D-Leu
**24a**	D-Leu	N-Me-Leu	Trp(Boc)	D-Orn(Boc)	Asn(Trt)	N-Me-Val
**25a**	D-Orn(Boc)	Asn(Trt)	N-Me-Val	D-Leu	N-Me-Leu	Trp(Boc)
**26a**	D-Orn(Boc)	N-Me-Asn(Trt)	Val	N-Me-D-Leu	Leu	Trp(Boc)
**27a**	Leu	D-Leu	Val	Asn(Trt)	D-Orn(Boc)	Trp(Boc)
**28a**	Leu	D-Leu	Ala	Asn(Trt)	D-Orn(Boc)	Trp(Boc)

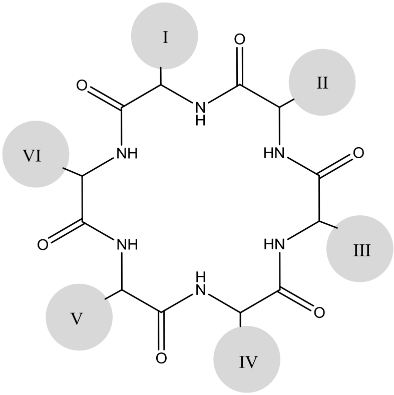

Table B. The cyclic precursors of the synthesized wollamide B analogues

Compound	I	II	III	IV	V	VI
**2b**	Ala	Trp (Boc)	D-Orn(Boc)	Asn(Trt)	Val	D-Leu
**3b**	Leu	Ala	D-Orn(Boc)	Asn(Trt)	Val	D-Leu
**4b**	Leu	Trp (Boc)	D-Orn(Boc)	Ala	Val	D-Leu
**5b**	Leu	Trp (Boc)	D-Orn(Boc)	Asn(Trt)	Ala	D-Leu
**6b**	Leu	Trp (Boc)	D-Orn(Boc)	Asn(Trt)	Val	D-Ala
**7b**	Leu	Trp (Boc)	D-Orn(Boc)	Asn(Trt)	*allo*-Ile	D-Leu
**8b**	Leu	Trp (Boc)	D-Orn(Boc)	Asn(Trt)	Ile	D-Leu
**9b**	Leu	Trp (Boc)	D-Orn(Boc)	Asn(Trt)	Met	D-Leu
**10b**	D-Leu	Leu	Trp(Boc)	D-Orn(Boc)	Asn(Trt)	Asp(Bzl)
**11b**	Leu	Trp (Boc)	D-Orn(Boc)	D-Orn(Boc)	Val	D-Leu
**12b**	D-Orn(Boc)	Ser (t-Bu)	Ile	D-Leu	Leu	Trp(Boc)
**13b**	D-Orn(Boc)	Ser (t-Bu)	Ile	D-Leu	Leu	Trp(Boc)
**14b**	Leu	Trp (Boc)	D-Orn(Boc)	Ile	Ile	D-Leu
**15b**	Leu	D-Leu	D-Phe(4-Cl)	D-Orn(Boc)	Asn(Trt)	Val
**16b**	Leu	D-Phe (4-OMe)	D-Orn(Boc)	Asn(Trt)	Val	D-Leu
**17b**	Val	D-Leu	D-Phe(4-Cl)	Trp(Boc)	D-Orn(Boc)	Asn(Trt)
**18b**	Leu	Trp (Boc)	D-Orn(Boc)	Asn(Trt)	Val	D-Phe (4-Cl)
**19b**	Leu	Trp (Boc)	D-Orn(Boc)	D-Orn(Boc)	Ile	D-Phe (4-Cl)
**20b**	Val	D-Leu	Ile	Trp(Boc)	D-Orn(Boc)	Asn(Trt)
**21b**	Val	Ile	Leu	Trp(Boc)	D-Orn(Boc)	Asn(Trt)
**22b**	Leu	Trp (Boc)	D-Arg(Pbf)	Asn(Trt)	Ile	D-Leu
**23b**	Leu	Trp (Boc)	D-Orn(Boc)	Asn(Trt)	N-Me-Val	D-Leu
**24b**	D-Leu	N-Me-Leu	Trp(Boc)	D-Orn(Boc)	Asn(Trt)	N-Me-Val
**25b**	D-Orn(Boc)	Asn(Trt)	N-Me-Val	D-Leu	N-Me-Leu	Trp(Boc)
**26b**	D-Orn(Boc)	N-Me-Asn(Trt)	Val	N-Me-D-Leu	Leu	Trp(Boc)
**27b**	Leu	D-Leu	Val	Asn(Trt)	D-Orn(Boc)	Trp(Boc)
**28b**	Leu	D-Leu	Ala	Asn(Trt)	D-Orn(Boc)	Trp(Boc)

^{1}H NMR spectra and UPLC data for selected wollamides

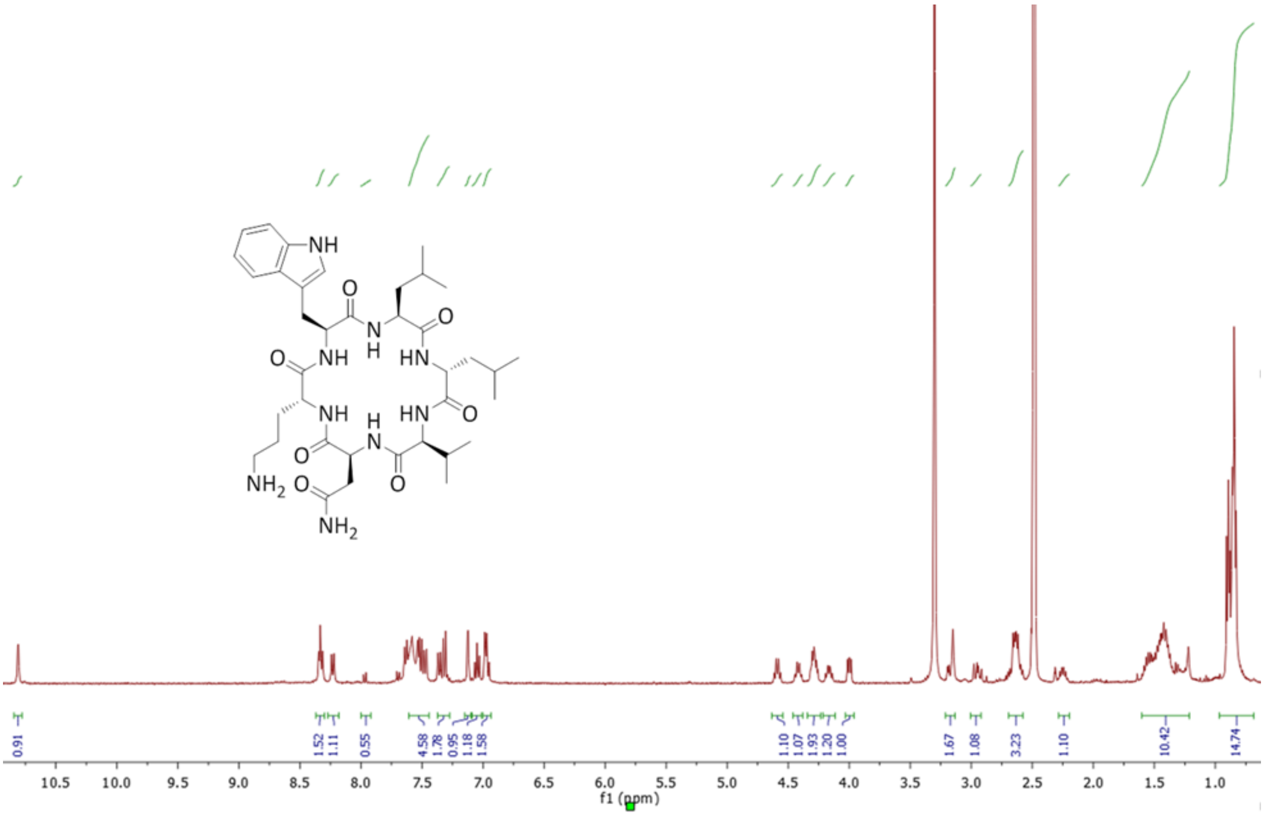

Fig A. ^{1}H NMR (400 MHz, DMSO-d6) spectrum of **1c**

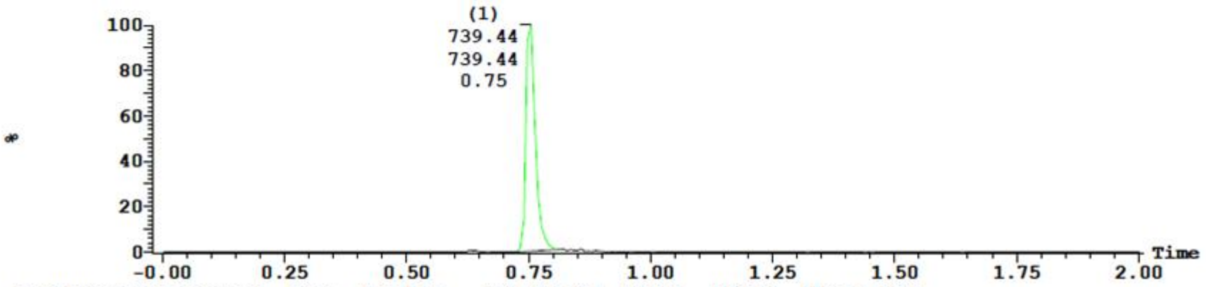

Peak Number	Compound	Time	AreaAbs	Area %Total	Width	Height	Mass Found
1	Found	0.75	3e+005	100.00	0	1e+007	739.44, 739.44

MS ES- :738.438 1.0000Da 5.9e+005

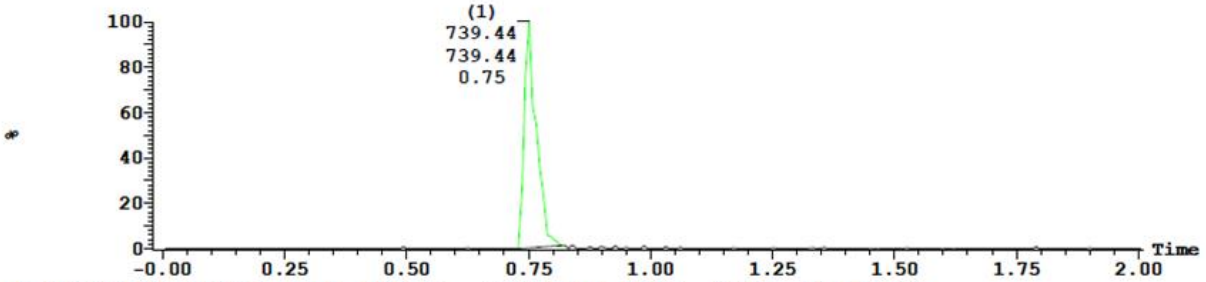

Peak Number	Compound	Time	AreaAbs	Area %Total	Width	Height	Mass Found
1	Found	0.75	2e+004	100.00	0	6e+005	739.44, 739.44

UV Detector: TIC 4.992e+1

Range: 4.992e+1

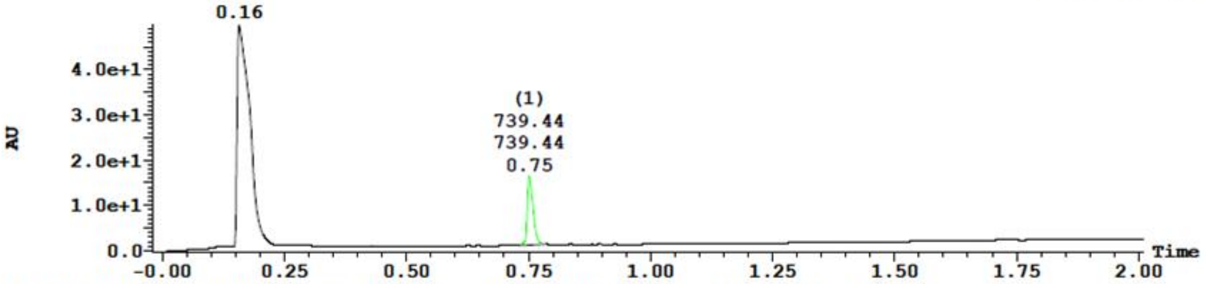

Peak Number	Compound	Time	AreaAbs	Area %Total	Width	Height	Mass Found
1	Found	0.75	2e+005	100.00	0	2e+007	739.44, 739.44

Fig B. The result of UPLC-MS quality checkup for **1c**

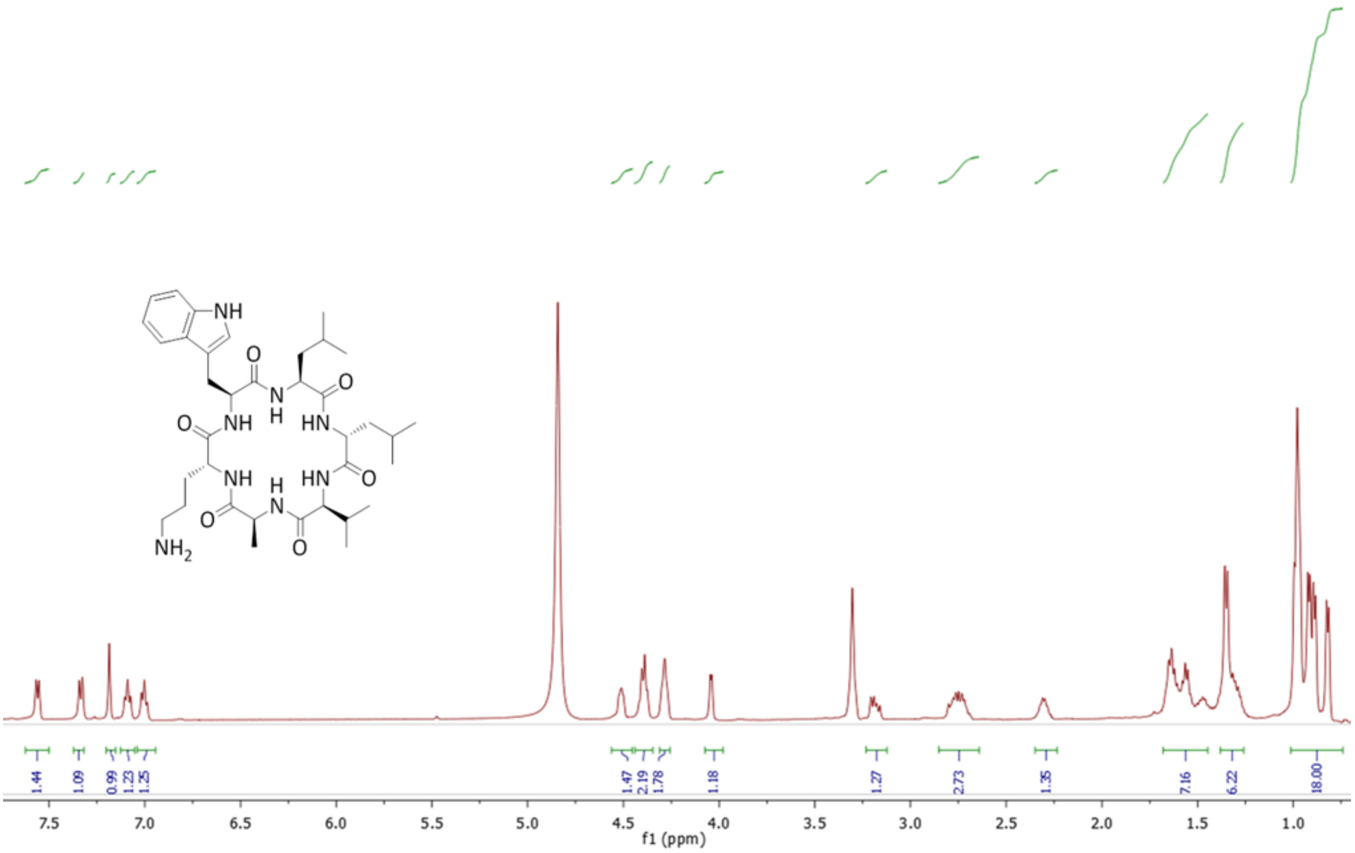

Fig C. ^{1}H NMR (500 MHz, CD_3OD) spectrum of **4c**

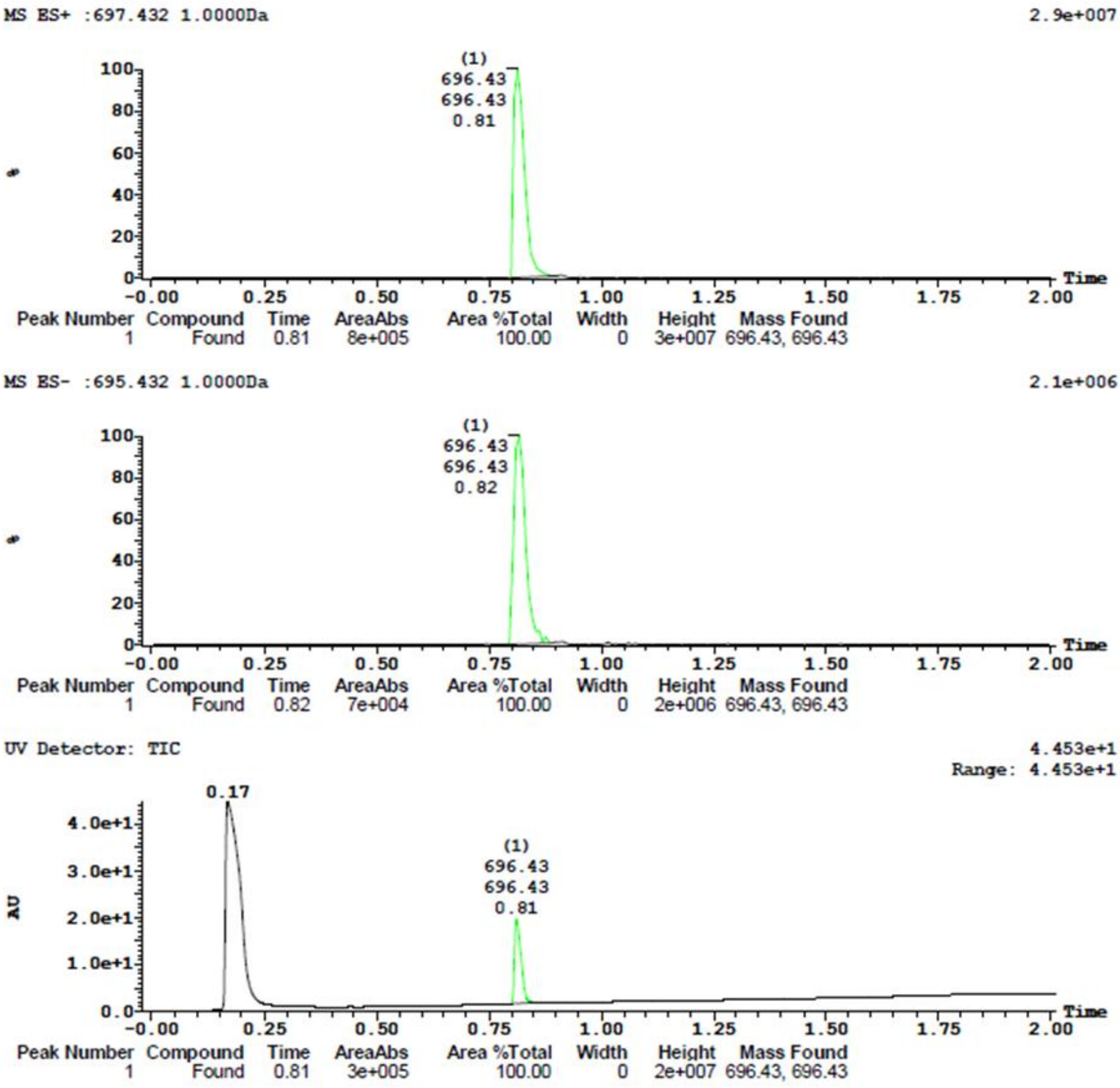

Fig D. The result of UPLC-MS quality checkup for **4c**

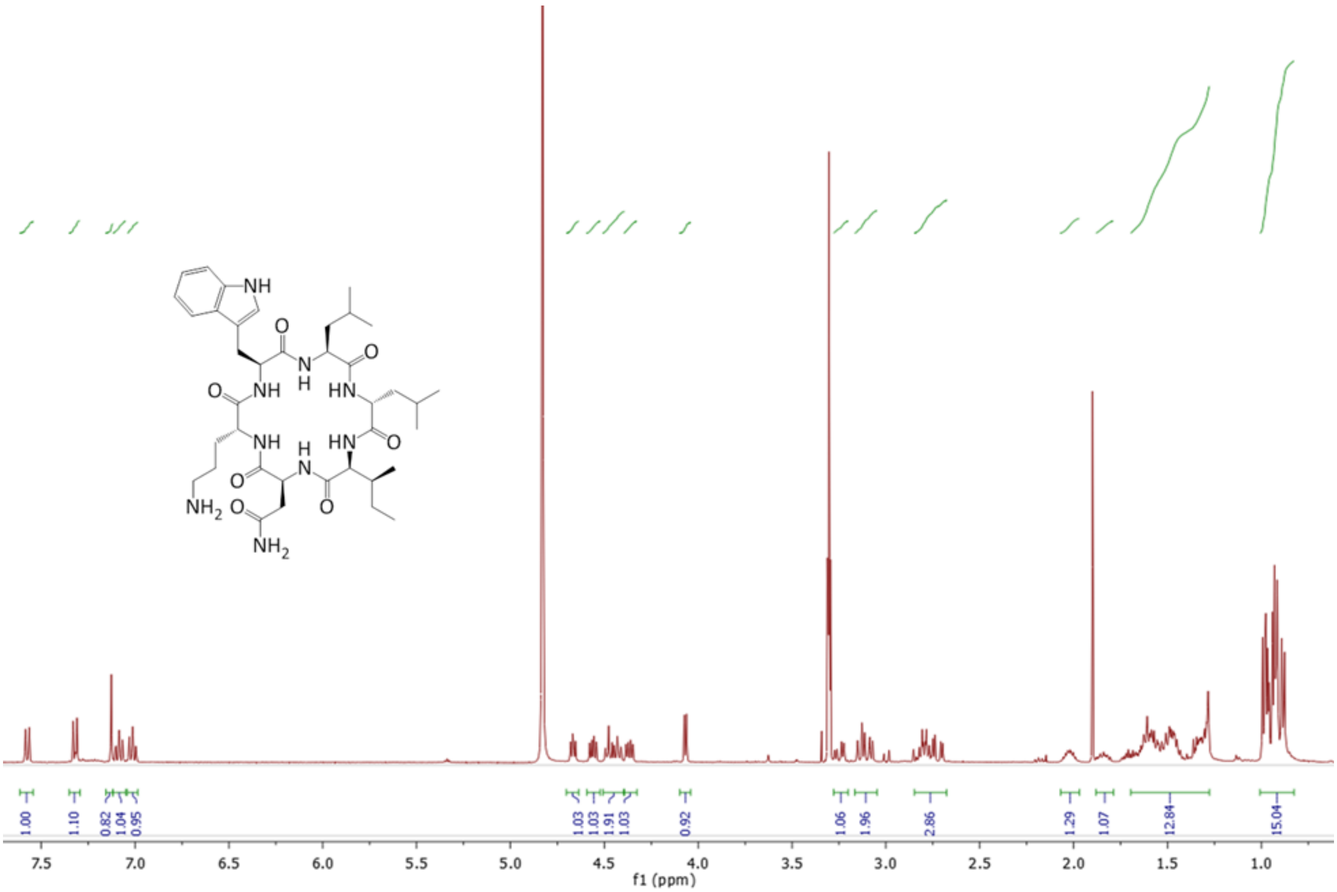

Fig E. ^{1}H NMR (400 MHz, CD_3OD) spectrum of **7c**

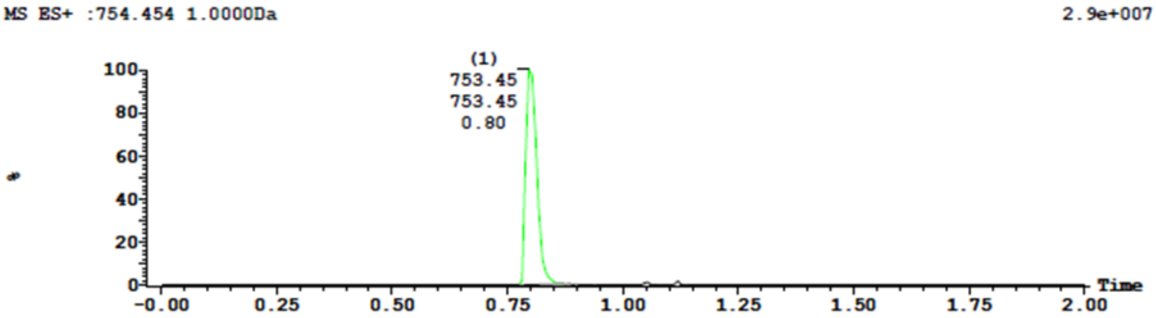

Peak Number	Compound	Time	AreaAbs	Area %Total	Width	Height	Mass Found
1	Found	0.80	8e+005	100.00	0	3e+007	753.45, 753.45

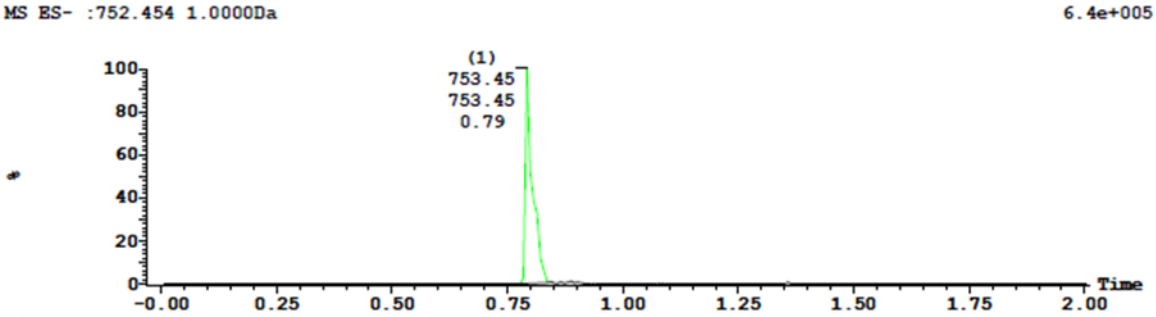

Peak Number	Compound	Time	AreaAbs	Area %Total	Width	Height	Mass Found
1	Found	0.79	1e+004	100.00	0	6e+005	753.45, 753.45

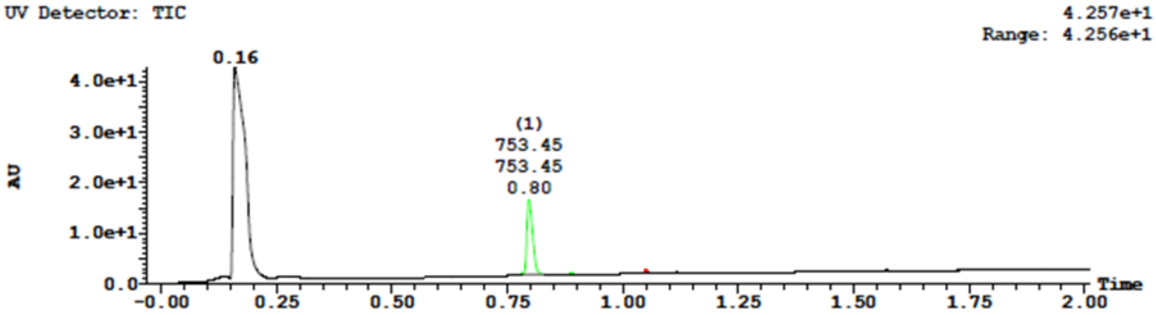

Peak Number	Compound	Time	AreaAbs	Area %Total	Width	Height	Mass Found
1	Found	0.80	2e+005	95.80	0	1e+007	753.45, 753.45
2	Tentative	0.89	2e+003	1.05	0	2e+005	753.45
3		1.05	7e+003	3.15	0	8e+005	

Fig F. The result of UPLC-MS quality checkup for **7c**

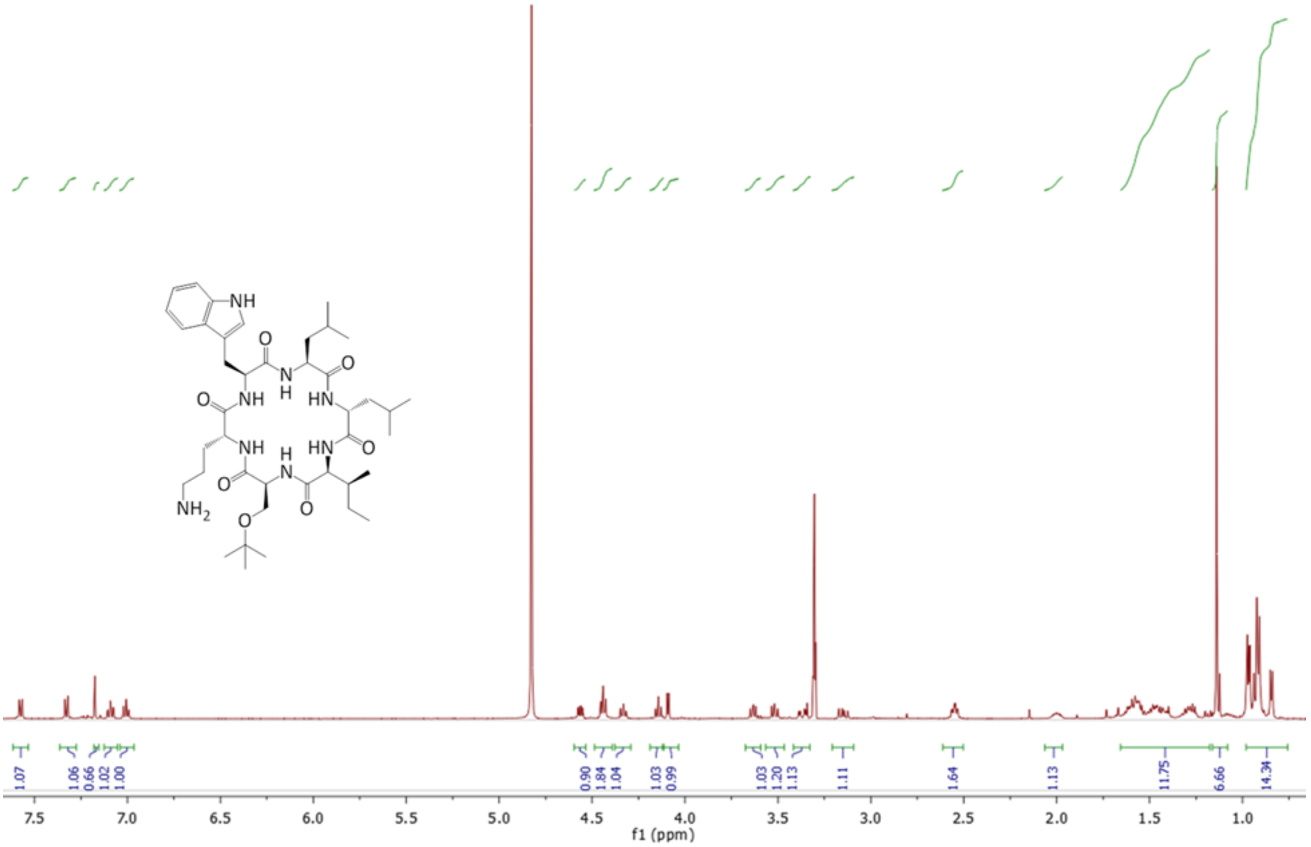

Fig G. ^{1}H NMR (500 MHz, CD_3OD) spectrum of **11c**

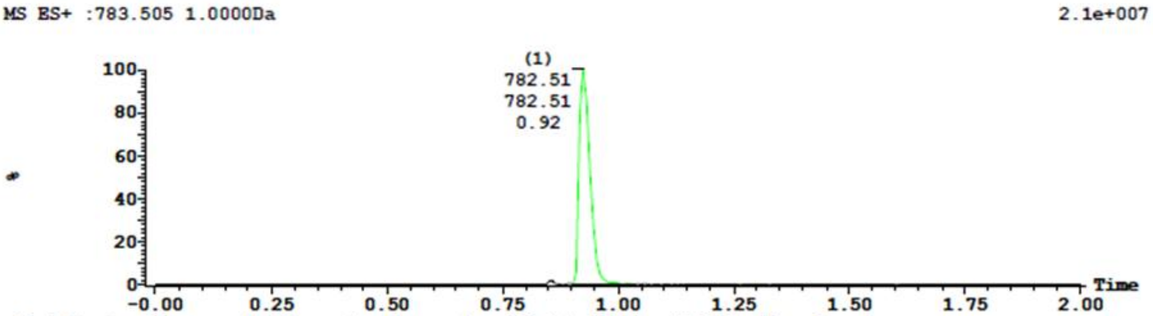

Peak Number	Compound	Time	AreaAbs	Area %Total	Width	Height	Mass Found
1	Found	0.92	6e+005	100.00	0	2e+007	782.51, 782.51

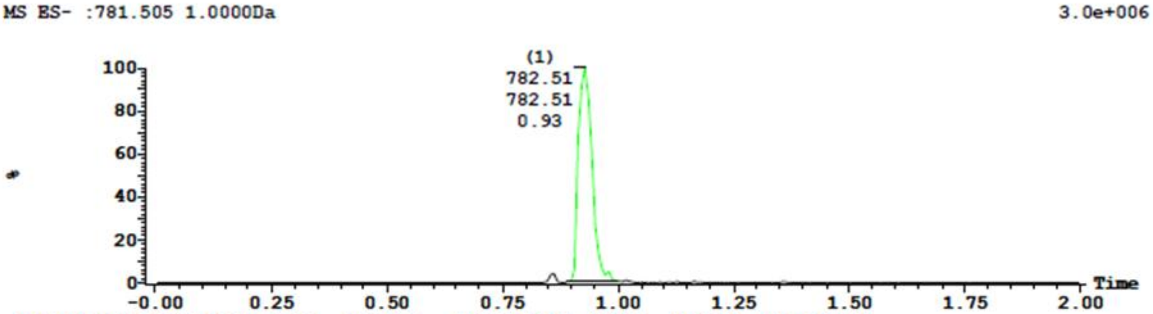

Peak Number	Compound	Time	AreaAbs	Area %Total	Width	Height	Mass Found
1	Found	0.93	1e+005	100.00	0	3e+006	782.51, 782.51

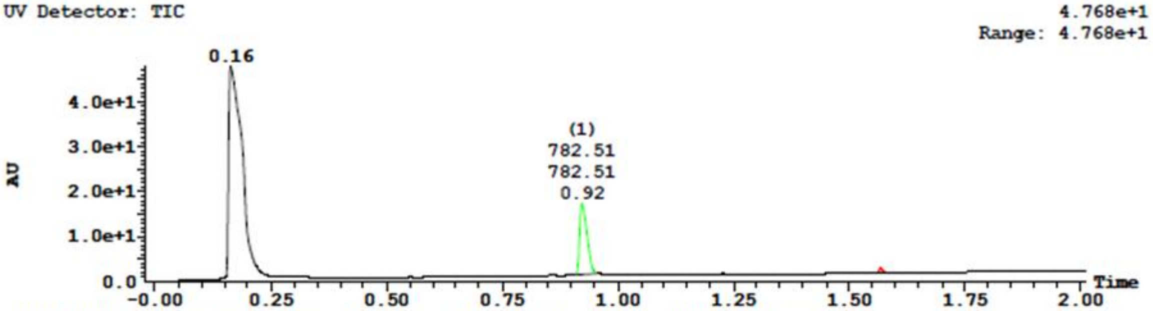

Peak Number	Compound	Time	AreaAbs	Area %Total	Width	Height	Mass Found
1	Found	0.92	3e+005	96.62	0	2e+007	782.51, 782.51
2		1.57	1e+004	3.38	0	1e+006	

Fig H. The result of UPLC-MS quality checkup for **11c**

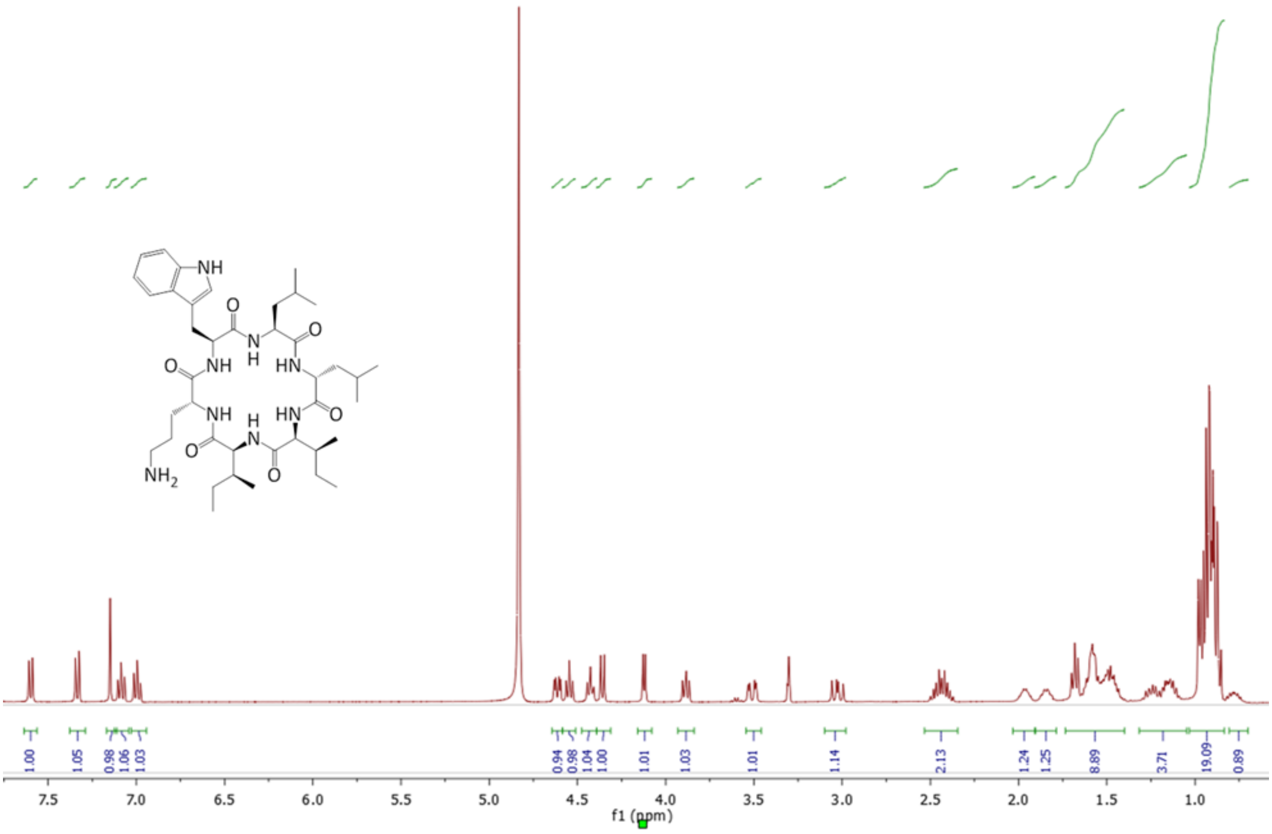

Fig I. ^{1}H NMR (400 MHz, CD_3OD) spectrum of **12c**

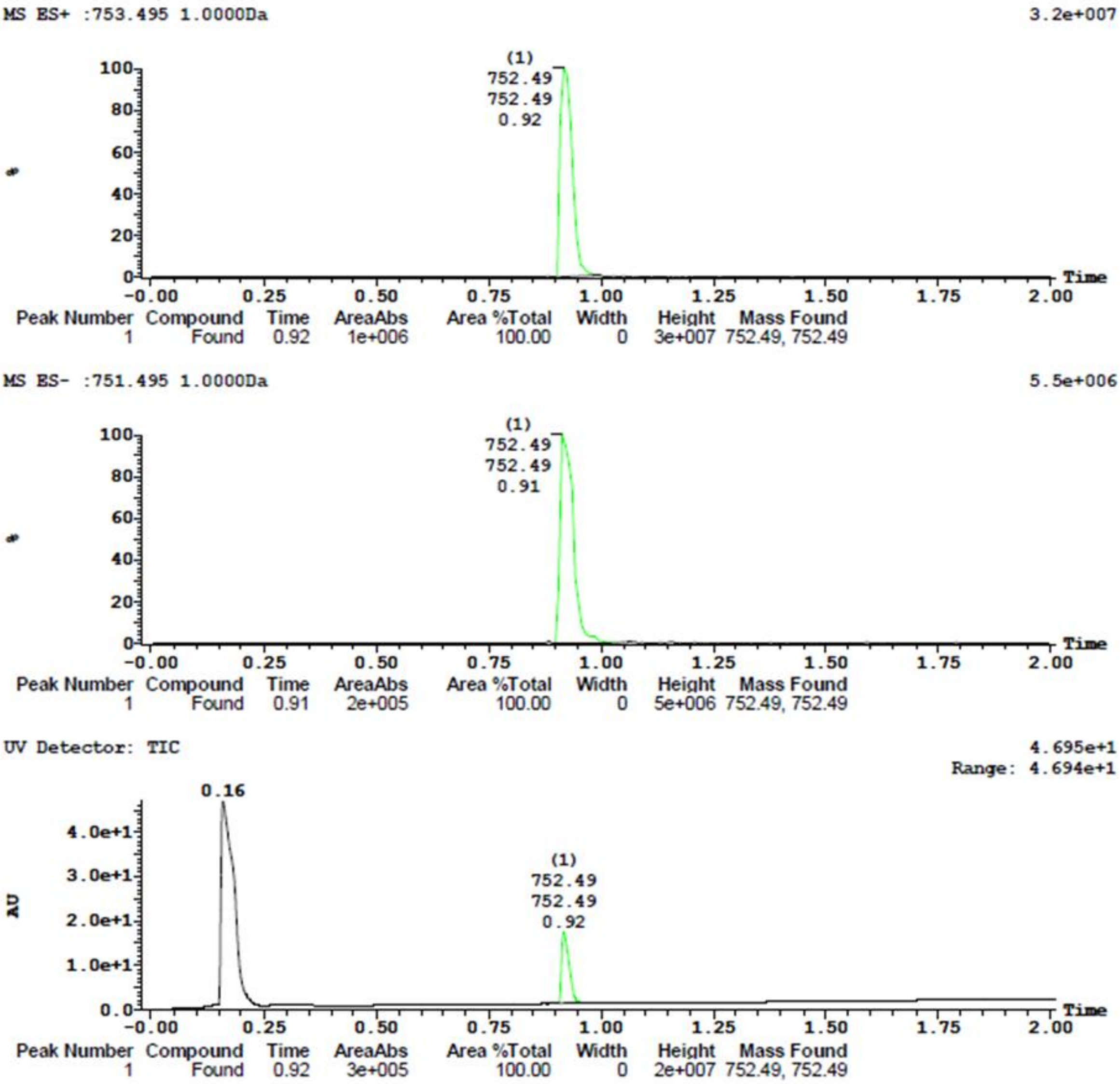

Fig J. The result of UPLC-MS quality checkup for **12c**

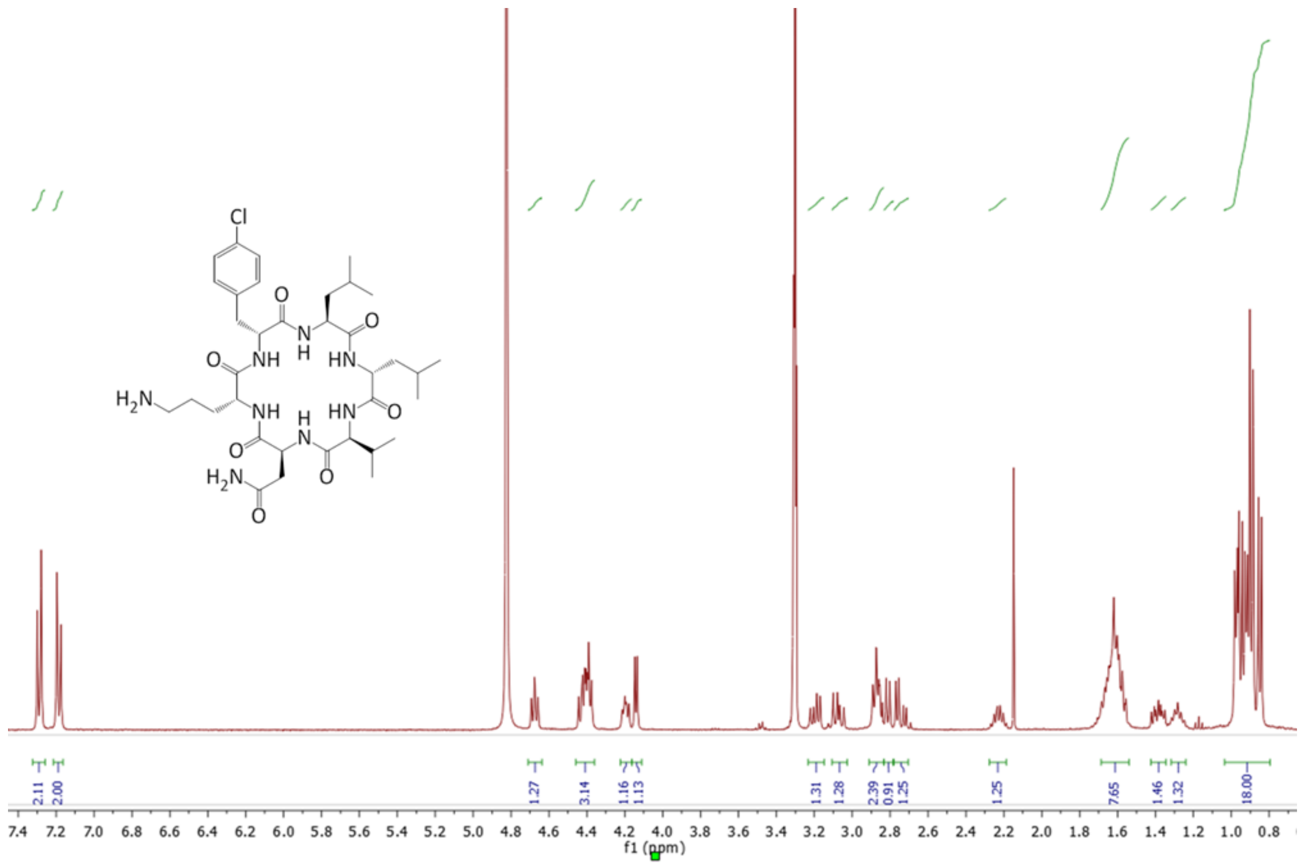

Fig K. ^{1}H NMR (400 MHz, CD_3OD) spectrum of **14c**

MS ES+ :735.388 1.0000Da 2.2e+007

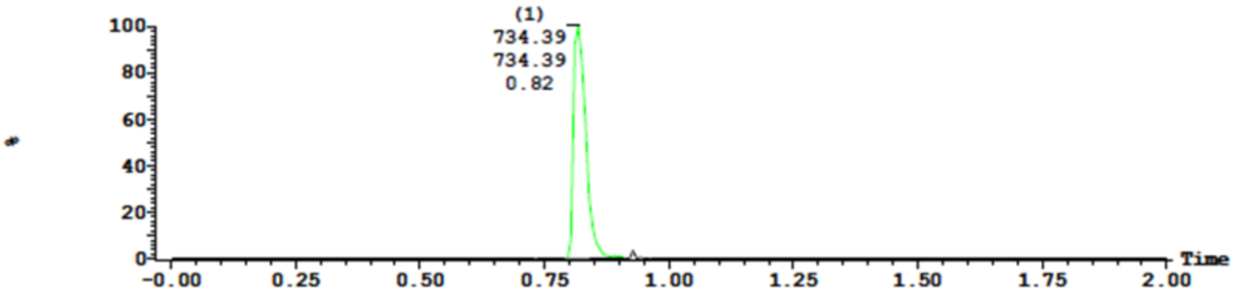

Peak Number	Compound	Time	AreaAbs	Area %Total	Width	Height	Mass Found
1	Found	0.82	6e+005	100.00	0	2e+007	734.39, 734.39

MS ES- :733.388 1.0000Da 7.1e+005

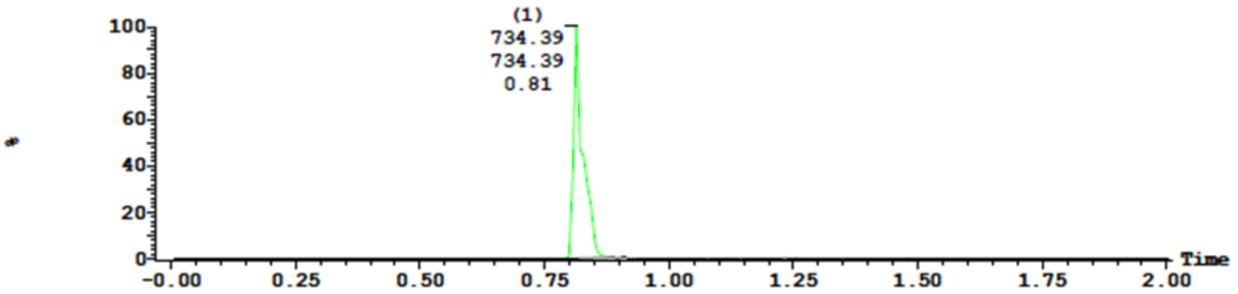

Peak Number	Compound	Time	AreaAbs	Area %Total	Width	Height	Mass Found
1	Found	0.81	2e+004	100.00	0	7e+005	734.39, 734.39

UV Detector: TIC 4.304e+1

Range: 4.304e+1

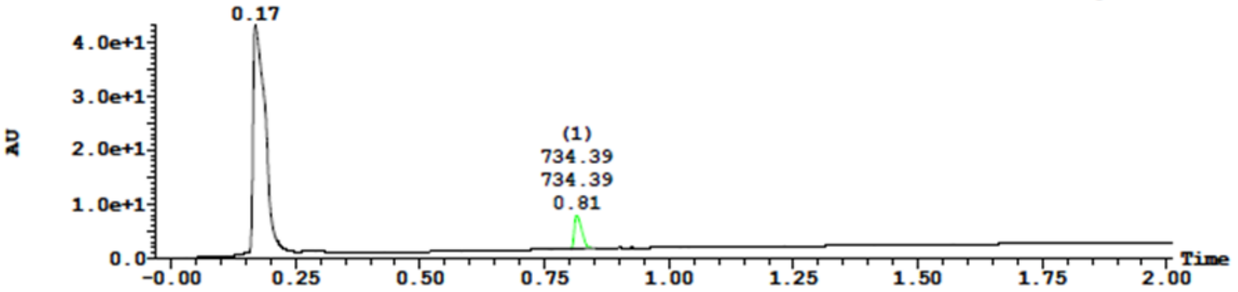

Peak Number	Compound	Time	AreaAbs	Area %Total	Width	Height	Mass Found
1	Found	0.81	1e+005	100.00	0	6e+006	734.39, 734.39

Fig L. The result of UPLC-MS quality checkup for **14c**
